# Investigation of *De Novo* Unique Differentially Expressed Genes Related to Evolution in Exercise Response during Domestication in Thoroughbred Race Horses

**DOI:** 10.1371/journal.pone.0091418

**Published:** 2014-03-21

**Authors:** Woncheoul Park, Jaemin Kim, Hyeon Jeong Kim, JaeYoung Choi, Jeong-Woong Park, Hyun-Woo Cho, Byeong-Woo Kim, Myung Hum Park, Teak-Soon Shin, Seong-Keun Cho, Jun-Kyu Park, Heebal Kim, Jae Yeon Hwang, Chang-Kyu Lee, Hak-Kyo Lee, Seoae Cho, Byung-Wook Cho

**Affiliations:** 1 Department of Agricultural Biotechnology and Research Institute for Agriculture and Life Sciences, Seoul National University, Seoul, Republic of Korea; 2 Interdisciplinary Program in Bioinformatics, Seoul National University, Seoul, Republic of Korea; 3 C&K genomics, Seoul National University, Seoul, Republic of Korea; 4 Department of Animal Science, College of Life Sciences, Pusan National University, Miryang, Republic of Korea; 5 TNT Research, Anyang, Republic of Korea; 6 Leaders in Industry-university Cooperation, Pusan National University, Miryang, Republic of Korea; 7 Genomic Informatics Center, Hankyong National University, Anseong, Republic of Korea; University of Lausanne, Switzerland

## Abstract

Previous studies of horse RNA-seq were performed by mapping sequence reads to the reference genome during transcriptome analysis. However in this study, we focused on two main ideas. First, differentially expressed genes (DEGs) were identified by *de novo*–based analysis (DBA) in RNA-seq data from six Thoroughbreds before and after exercise, here-after referred to as “*de novo* unique differentially expressed genes” (DUDEG). Second, by integrating both conventional DEGs and genes identified as being selected for during domestication of Thoroughbred and Jeju pony from whole genome re-sequencing (WGS) data, we give a new concept to the definition of DEG. We identified 1,034 and 567 DUDEGs in skeletal muscle and blood, respectively. DUDEGs in skeletal muscle were significantly related to exercise-induced stress biological process gene ontology (BP-GO) terms: ‘immune system process’; ‘response to stimulus’; and, ‘death’ and a KEGG pathways: ‘JAK-STAT signaling pathway’; ‘MAPK signaling pathway’; ‘regulation of actin cytoskeleton’; and, ‘p53 signaling pathway’. In addition, we found *TIMELESS*, *EIF4A3* and *ZNF592* in blood and *CHMP4C* and *FOXO3* in skeletal muscle, to be in common between DUDEGs and selected genes identified by evolutionary statistics such as F_ST_ and Cross Population Extended Haplotype Homozygosity (XP-EHH). Moreover, in Thoroughbreds, three out of five genes (*CHMP4C*, *EIF4A3* and *FOXO3*) related to exercise response showed relatively low nucleotide diversity compared to the Jeju pony. DUDEGs are not only conceptually new DEGs that cannot be attained from reference-based analysis (RBA) but also supports previous RBA results related to exercise in Thoroughbred. In summary, three exercise related genes which were selected for during domestication in the evolutionary history of Thoroughbred were identified as conceptually new DEGs in this study.

## Introduction

Since domestication, at around 3500 B.C.E, horses have mainly been used for riding and racing [1]. One domesticated breed of horses, the Thoroughbred, has been specifically bred for speed, endurance, and strength since the 18^th^ century. The extreme selection for these traits has resulted in a highly adapted athlete [2] with very high aerobic capacity [3], and high skeletal muscle mass [4], which comprises over 55% of total body mass [5]. The Thoroughbred is an excellent breed for competitive horse racing and by extension a valuable model for studying exercise response. A previous study has shown that exercise training in Thoroughbreds resulted in coordinated changes in the expression of genes related to metabolism, oxidative phosphorylation and muscle structure [6].

Domestication leads to gradual changes at the genetic level by a process of selection in a population of animals or plants. Most domestic animals were selectively bred for the goal of benefitting human beings. Due to the combined effect of natural selection and human-controlled selective breeding, phenotypic changes, which are related to genetic mutation, accompany the domestication process. Some genetic mutations with beneficial phenotypic effects have been either highly enriched or vanished by selective sweeps [7]. A selective sweep is the reduction of genetic diversity in the neighboring DNA of a fixed mutation. Selective sweep regions in the genome can potentially be identified by a genome scan, and the low variation interval surrounding the selected gene can be found by fine-scale mapping. Using such genome scans, selective sweeps have been identified in domestic and natural (wild progenitor) populations [8], [9].

Previous horse transcriptome studies using RNA-seq were carried out by mapping sequence reads to a reference genome. However, reference genome assembly has been known to have flaws including missing expressed genes [10], hundreds to thousands of miss-assemblies and large genomic deletions [11], and problems in trans-spliced genes [12]. Therefore, the results and success of reference transcriptome assembly depends on both the availability and quality of the reference genome. On the contrary, *de novo* transcript assembly has several advantages. First, it does not depend on a reference genome [13]. This is a key advantage as many organisms do not have a high-quality finished reference genome. For these organisms, *de novo* assembly becomes the first analysis step. Also, it does not depend on the correct alignment of reads to known splice sites [14] or the prediction of novel splicing sites, both of which are required by reference-based assemblers. Trans-spliced transcripts and similar transcripts originating from chromosomal rearrangements can be assembled using the *de novo* approach. In addition, *de novo* transcriptome assembly can help researchers investigate genes that are absent in the reference genome due to the incompleteness of reference sequences [10]. Lastly, it can identify new transcripts and new transcript structures [15], [16]. However, reconstruction of full-length transcripts from short reads with considerable sequencing error rates poses substantial computational challenges [17]. Still, *de novo* assembly in RNA-seq is an important approach for carrying out transcriptomic studies.

Recently, many *de novo* assembly software tools have been developed, most of which take the de Bruijn graph approach. This approach usually has two important parameters: k-mer length and coverage cutoff value [16]. Tools such as Trans-ABySS [15], Trinity [17], ABySS [18], Oases [19], Rnnotator [20], Multiple-k [21], SOAPdenovo [22] and Velvet [23] follow this approach. Considering these *de novo* assembly software tools, *Manfred G Grabherr, et.al* concluded that Trinity *de novo* assembly software tool is superior to others for a number of reasons. Specifically, Trinity fully reconstructs a large fraction of transcripts, including alternatively spliced isoforms and transcripts from recently duplicated genes. In addition, Trinity resolves ∼99% of the initial sequencing errors, determines splice isoforms, and distinguishes transcripts from recently duplicated and identified allelic variants [17].

In this study, we used *de novo*-based analysis (DBA) to identify differentially expressed genes (DEGs) that could not be identified with reference-based analysis (RBA). Furthermore, we integrate DEGs with genes identified as being selected for during domestication to reveal genes that are related to the evolution of exercise response during the domestication process of the Thoroughbred.

## Materials and Methods

### Ethics statement

This study was carried out in strict accordance with the recommendations in the Guide for the Care and Use of Laboratory Animals of Pusan National University. All experimental procedures used in this study were approved by the Institutional Animal Care and Use Committee of the Pusan National University (PNU-2013-0417). The owners of the Thoroughbred horses gave permission for their animals to be used in this study.

### Analysis of horses RNA-seq data

#### 1. RNA-seq data between before and after exercise

We generated RNA-seq data from six Thoroughbred horses before and after exercise as described in a previous study [24]. Samples of skeletal muscle and blood were taken from six Thoroughbreds before and after exercise. ‘Before exercise’ samples were collected from the triceps brachii of the right leg and from the jugular vein and carotid artery of each horse. After an adequate resting period of several hours, the horses were subjected to a 30-min trot. Then, immediately after this trot, the ‘after exercise’ samples were collected from the same tissues of each individual. Thoroughbreds usually canter for 17–18 min per day. For the purposes of this study, a 30-min trot was taken to be the equivalent to 17–18 min of cantering. Total RNA from the skeletal muscle and blood samples were isolated using TRIzol (Invitrogen) and the RNeasy RNA purification kit with DNase treatment (Qiagen). mRNA was isolated from the total RNA using oligo-dT beads, then reverse transcribed into double-stranded cDNA fragments. Construction and sequencing of an RNA sequence library for each sample was carried out based on Illumina HiSeq2000 protocols in order to generate 90 bp paired-end reads. Twenty-four sets of transcriptome data were generated from muscle and blood samples of six horses obtained before and after exercise.

The RNA-sequencing data from this study have been submitted to the NCBI Gene Expression Omnibus (GEO) (http://www.ncbi.nlm.nih.gov/geo/) under the accession number GSE37870.

#### 2. *De novo*-based analysis (DBA)

We used the Trinity *de novo* assembly software tool [17], [25] following the default settings, except for the following options: number of CPU and alignment method: bowtie. First, Trinity tools generated a reference for each obtained sample (a total of 24 samples) to detect DEGs. Second, Trinity tools generated a reference for each individual (a total of 4 samples) to compare with the SNPs from the whole genome sequence, RNA-seq (using reference transcriptome assembly) and RNA-seq (using *de novo* assembly).

The component ID was converted to known transcript ID (ftp://ftp.ensembl.org/pub/release-73/fasta/equus_caballus/cdna/) using Blastall [26], a user-friendly, free open source tool, which is suited for short read alignment. After conversion, we filtered the transcript ID by alignment length of higher than 80%, more detail about the *de novo* assembly method is given in the Supplementary Methods (File S1).

Reference-based analysis (RBA): Most of the RBA used in this study is described in Kim et al [27]. TopHat [28] (ver.1.4.1) was used to map the sequences to a horse reference genome and annotated using the EquCab2 database (http://hgdownload.cse.ucsc.edu/downloads.html#horse).

#### 3. DEG selection (*de novo* vs reference)

We examined the differential expression of replicated count data by applying a method based on negative binomial model as implemented in the R package EdgeR [29]. This package was used because RNA sequence data may exhibit more variability than expected in a Poisson distribution due to wide dispersal in the genome. The method implemented in the EdgeR package automatically takes all known sources of variation into account. Significant DEGs were detected with a cut-off value of FDR<0.01, based on a paired design between‘before exercise’ and ‘after exercise’.

#### 4. Genotype calling and SNP calling (*de novo* vs reference)

Three open source packages were used for downstream processing and variant calling: Picard Tools (http://picard.sourceforge.net), SAMtools [30] and the genome Analysis Toolkit (GATK [31]). Substitution calls were made with the GATK Unified Genotyper [32]. All calls with a Phred-scaled quality of less than 30 were filtered out and VCFtools [33] was used for handling the vcf file format.

#### 5. DAVID analysis (*de novo* vs reference)

One simple but extremely widely used systems biology technique for highlighting biological processes is gene category over-representation analysis. In order to perform this analysis, genes are grouped into categories by a common biological property and then tested to find categories that are over represented among the differentially expressed genes. Gene Ontology (GO) categories are commonly used in this technique and there are many tools available for performing GO and KEGG pathway analysis. We used DAVID [34] web tool to convert the equine Ensembl gene IDs to official gene symbols. This was carried out by cross-matching the equine Ensembl gene IDs to the human Ensembl gene IDs and the official gene symbols. The representation of functional groups in blood and skeletal muscle relative to the whole genome was investigated using the Expression Analysis Systematic Explorer (EASE) tool [35] within DAVID. The EASE tool is a modified Fisher's exact test used to measure enrichment of gene ontology (GO) terms [36]. To identify enriched GO terms, functionally clustered genes were filtered by an EASE value of <0.01. In addition, A KEGG pathway enrichment test was performed using EASE, with a cut-off value <0.1

#### 6. Quantitative real-time reverse transcript-PCR (qRT-PCR) validation

A blood sample was obtained from a Thoroughbred horse maintained at Ham-an Racing Horse Resort & Training Center before and after exercise. Exercise was performed as a 30-min trotting on a treadmill. Trizol reagent (Invitrogen) was used to extract total RNA from leukocytes after exercise, according to the Invitrogen manual. In order to prevent contamination of genomic DNA, RNase-free DNase kit (Qiagen) was used according to the manufacturer's operating manual. Total RNA quantification was performed by using NanoDrop® ND-1000 Spectrophotometer. cDNAs were synthesized in a reaction with oligo-dT primers, moloney-murine leukemia virus (MMLV) reverse transcriptase (Promega), RNase inhibitor (Promega) and RNase-free ddH_2_O, which was incubated at 37°C for 4 h.

To confirm the *de novo* unique differentially expressed genes revealed by RNA-Seq, seven DUDEGs were analyzed by RT-PCR amplification. The primers were designed using the PRIMER3 software (http://frodo.wi.mit.edu/primer3/) (Table S9 in File S1).

The RT-PCR conditions were as follows: an initial step of 94°C for 10 min, 35 cycles of 94°C for 30 sec, 60°C for 30 sec, 72°C for 30 sec, and final step of 72°C for 10 min. PCR bands were normalized with *glyceraldehyde-3-phosphate dehydrogenase* (GAPDH) band. RT-PCR products were visualized by gel electrophoresis on a 2.0% SeaKem LE agarose gel.

cDNA was analyzed by BioRad CFX-96. All samples were measured in triplicate to ensure reproducibility, and C_t_ value was calculated using 2^−ΔΔCt^ method [37].

### Analysis of horse whole genome re-sequencing data

#### 1. Whole genome re-sequencing data of Thoroughbred and Jeju domestic ponies

Whole-blood samples were collected from 18 Thoroughbred racing stallions of the Korean Racing Authority, and from four male and two female Jeju domestic ponies (*Equus caballus*) of the Jeju Provincial Livestock Institute, Korea. A 10 mL sample of blood was drawn from the carotid artery of each horse and was treated with heparin to prevent clotting. Genomic DNA was extracted and a quality check was carried out using fluorescence-based quantification on an agarose gel, a standard electrophoresis on a 0.6% agarose gel and, via a pulsed-field gel, using 200 ng of DNA. Manufacturers' instructions were followed to create a paired library of 500-bp fragments. This consisted of the following: purified genomic DNA fragments of less than 800 bp, fragments with blunt ends, fragments with 5′ phosphorylated ends, fragments with a 3′- dA overhang, some with adaptor-modified ends, purified ligation product, and a genomic DNA library. Following this, we generated sequence data using HiSeq 2000 (Illumina, Inc).

#### 2. Reference genome assembly

Using the Burrows-Wheeler Aligner [38] with the default setting, pair-end sequence reads were mapped to the reference horse genome (ftp://ftp.ensembl.org/pub/release-73/fasta/equus_caballus/dna/) (Table S8 in File S1). The DNA re-sequencing data from this study have been submitted to the NCBI Sequence Read Archive (SRA) database under the accession numbers SRA053569, SRA054885 and SRP017702.

#### 3. Genotype calling and SNP calling

We used the following open-source software packages; Picard Tools, SAMtools, and the Genome analysis toolkit, for downstream processing and variant calling. Substitution calls were made with GATK UnifiedGenotyper20 and all calls with a Phred-scaled quality of less than 30 were filtered out. For each chromosome, we simultaneously inferred the phased haplotype and inputed the missing alleles for the entire set of Thoroughbred populations using BEAGLE [39].

#### 4. Estimation of Nucleotide diversity, F_ST_ and Cross Population Extended Haplotype Homozygosity (XP-EHH) value

Nucleotide diversity and long run of homozygosity (LROH) of Thoroughbred and Jeju domestic ponies for each chromosome were calculated by VCFtools. Conventional F_ST_ [40] and Reynolds F_ST_ [41] values were calculated for genes using Arlequin 3.5 [42] based on pairwise differences between the haplotypes of Thoroughbred and Jeju domestic ponies. In order to calculate F_ST_, we used the horse genome to phase the haplotypes of the two populations. Also, to calculate F_ST_ by each gene region, we used the genomic information (Ensembl Genes71, EquCab2), namely the Ensembl reference annotated gene information. We selected the genes of the top 1% of the empirical distribution (empirical p-value<0.01) [43]. The method Cross Population Extended Haplotype Homozygosity (XP-EHH) was used to detect selective sweeps using the software xpehh [44] (http://hgdp.uchicago.edu/Software/). For XP-EHH analysis, we used haplotype information for all SNPs of the entire autosome, and we calculated Extended Haplotype Homozygosity (EHH) and the log-ratio integrated EHH (iHH) for the pairwise test of the Thoroughbred and Jeju domestic pony populations. The log ratios were standardized to have a mean of 0 and a variance of 1, and p-values were assigned assuming a normal distribution. We selected SNPs with p-values<0.01, which are considered to have strong selection signals. Then we apply a cutoff value of XP-EHH values<0 for finding adaptation in the Thoroughbred. We chose genes related with these SNPs by identifying genes located within a 10 kb [45] boundary of these SNPs. Since XP-EHH is not sensitive to allele frequencies, there is no need to stratify the data into frequency bins before determining significance. The p-values are empirical p-values; that is, a low p-value indicates that a locus is an outlier with respect to the rest of the genome. However, we note that loci detected as being under selection using this approach may be an under-representative sample of all truly selected loci; in particular, selection on standing variation and recessive loci are likely to be underrepresented [43].

## Results

### Differences in the results of reference-based and *de novo*-based assembly and analysis

**Figure 1:**
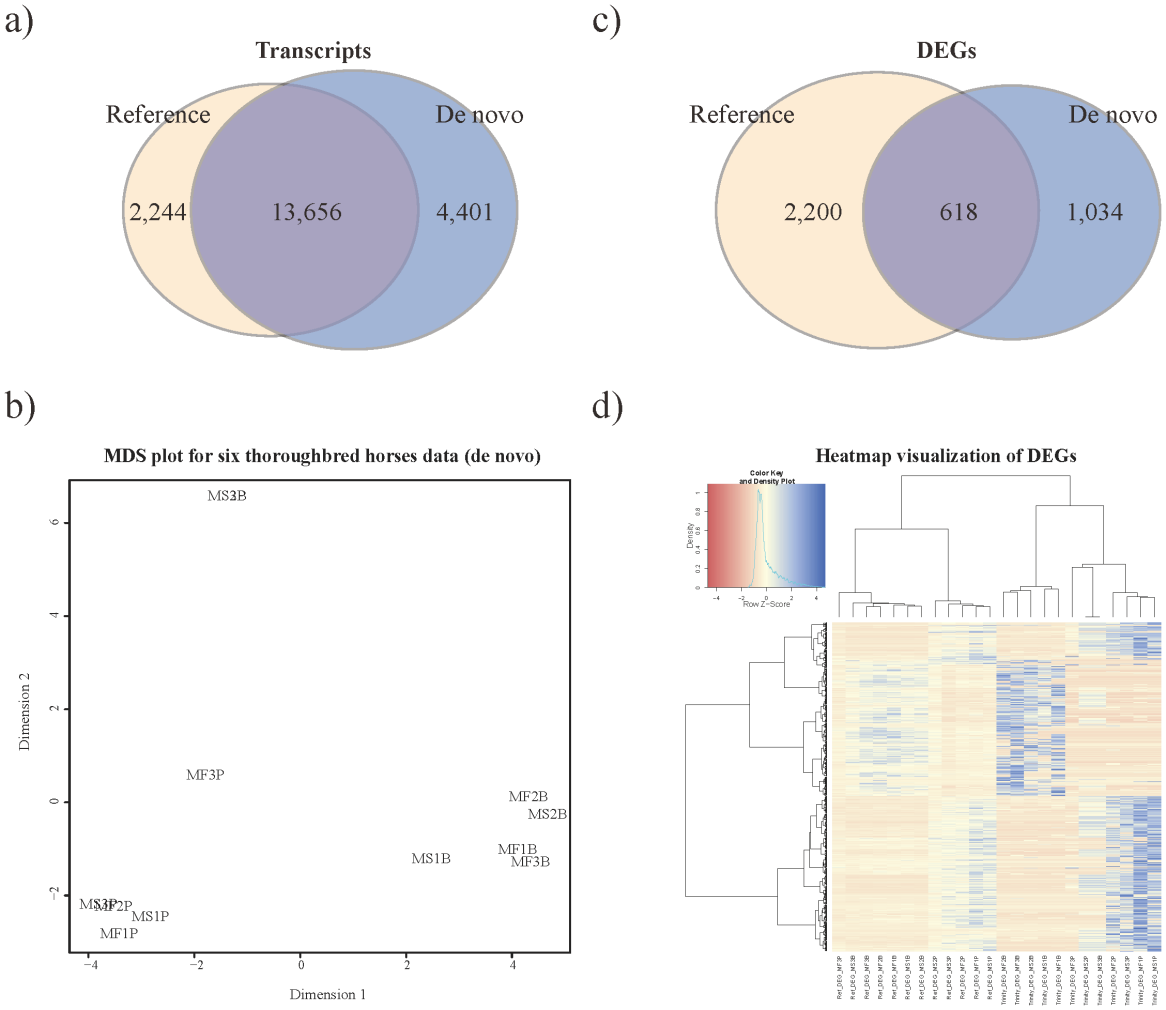
Summary of comparative analysis between *de novo* assembly and reference genome assembly from RNA-seq data of skeletal muscle from six Thoroughbreds before and after exercise (Total 12 samples). a) The number of transcripts in common between de novo assembly and reference genome assembly b) MDS plot of six Thoroughbreds before and after exercise using de novo assembly. c) The number of DEGs between de novo assembly and reference genome assembly. d) Heat-map visualization of common DEGs between de novo assembly and reference genome assembly: rows represent DEGs from skeletal muscle and columns represent assemble method from 6 horse samples (*First ‘B’ is for blood and ‘M’ is for muscle. ‘F1’, ‘F2’, ‘F3’ and ‘S3’ are horse samples. Last ‘B’ is for ‘before exercise’ and ‘P’ is for ‘after exercise’).

Transcriptome analysis results of reference-based analysis (RBA) and *de novo*-based analysis (DBA) showed a substantial difference in the number of transcript and differentially expressed genes (DEGs) identified. In RBA, for blood and skeletal muscle, 15,900 and 17,927 transcripts were found, respectively, among which 2,244 and 1,405 were unique transcripts. In DBA, the numbers of transcripts in skeletal muscle and blood were 18,057 and 19,413, respectively with 4,401 and 2,892 unique transcripts. The numbers transcripts in common between RBA and DBA were 13,656 for skeletal muscle tissue and 16,521 for blood (Figure 1a and Figure S1a in File S1). When the sample variance of RBA and DBA in skeletal muscle and blood were compared using multidimensional scaling (MDS) plot, the results for the two analyses were almost identical. The skeletal muscle samples were clustered into two subgroups: before and after exercise, but the blood samples did not show any clustering (Figure 1b, Figure S1b and Figure S2 in File S1). In RBA, the number of DEG in skeletal muscle and blood were 2,818 and 455, respectively with 2,200 and 427 DEGs being unique to RBA. In DBA, the number of DEG in skeletal muscle and blood were 1,652 and 595, respectively with 1,034 and 567 unique DEGs. The number of DEGs identified by both RBA and DBA were 618 and 28 in skeletal muscle and blood, respectively (Figure 1c and Figure S1c in File S1). These DEGs were compared using Heatmap visualization to examine their expression pattern in each analysis. The expression pattern was similar, however, the intensity of the expression was higher with DBA (Figure 1d and Figure S1d in File S1). Overall, in comparison to RBA, DBA identified a higher number of transcripts but a lower number of DEGs.

We detected SNPs from two different next-generation sequencing methods (WGS and RNA-seq) and two different assembly methods (RBA and DBA) for each Thoroughbred sample (F1, F2 and F3 = male, S3 = female). The number and rate of SNPs in DBA of RNA-seq was 108,158 (0.031%), 110,502 (0.031%), 105,920 (0.03%) and 101,887 (0.029%) in F1, F2, F3 and S3 respectively, and the number and rate of SNPs in from RBA of RNA-seq were 284,859 (0.012%), 287,286 (0.012%), 276,241 (0.011%) and 265,729 (0.011%) in F1, F2, F3 and S3, respectively (Table S2 in File S1).

### Identification of *de novo* unique differentially expressed genes (DUDEGs) before and after exercise

We identified DUDEGs from RNA-seq data using the expression profiles of genes in skeletal muscle and blood samples taken from six Thoroughbreds before and after exercise. There were a total of 1,034 significant DUDEGs (519 up-regulated, 515 down-regulated) in skeletal muscle and 567 (314 up-regulated, 253 down-regulated) in blood (FDR<0.01). Among them, 456 (61 up-regulated, 395 down-regulated) in skeletal muscle and 205 (93 up-regulated, 112 down-regulated) in blood were annotated (Table S1 in File S1).

### Validation of DUDEGs in horse RNA-seq data using RT-PCR

We performed RT-PCR to validate the DUDEGs detected in horse blood. The seven genes (*TIMELESS, EIF4A3, PGIW, ANK3, MSH3, SYNRG, ASGR2*: 2 up-regulated and 5 down-regulated) were randomly selected with conceptually new DEGs and logFC>2 in blood (Table S1, S4 and S5 in File S1). The expression levels of DUDEGs between RNAseq and RT-PCR were highly similar (Figure S8 in File S1). The results confirmed that DUDEGs identified in this study were reliable.

### Functional annotation of DUDEGs

**Figure 2:**
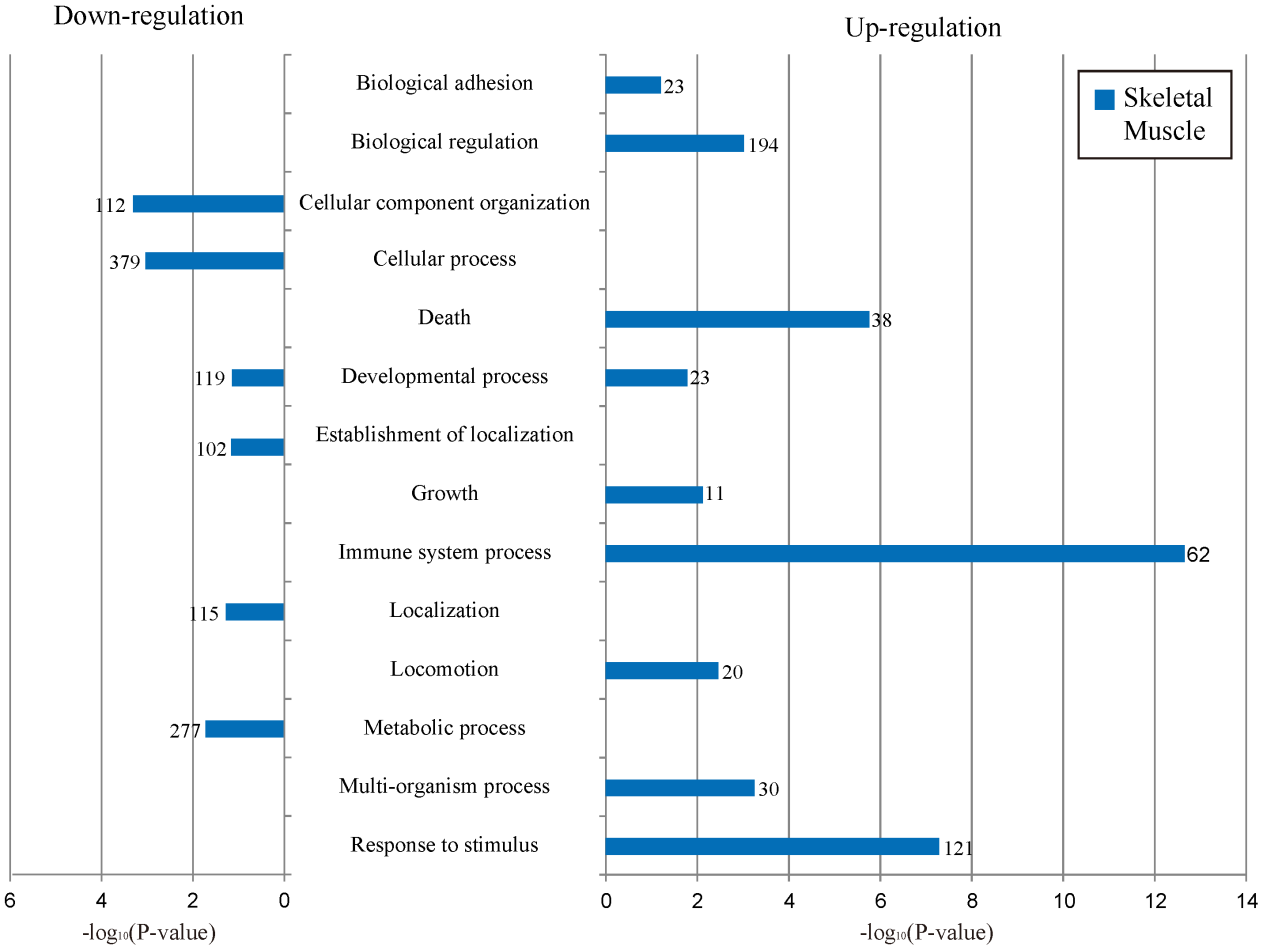
Biological process GO terms of tissues specific DEGs between before and after exercise in Thoroughbred. Up–regulated genes indicate higher activation after exercise than before and down-regulation genes indicate lower activation after exercise than before exercise.

We summarized the highest biological process gene ontology (BP-GO) of DUDEGs in skeletal muscle sample taken before and after exercise from six Thoroughbred RNA-seq data (Figure 2). The other BP-GO of DUDEGs were summarized separately (Figure S3, Figure S4, Figure S5 and Figure S6 in File S1). The most significantly enriched terms among up-regulated genes in skeletal muscle were ‘biological adhesion’, ‘biological regulation’, ‘death’, ‘growth’, ‘immune system process’, ‘locomotion’, ‘multi-organism process’, and ‘response to stimulus’ (P-value = 6.29E-02, P-value = 9.57E-04, P-value = 1.75E-06, P-value = 7.56E-03, P-value = 2.23E-13, P-value = 3.48E-03, P-value = 5.71E-04, and P-value = 5.16E-08, respectively). While, the most significantly enriched terms among down-regulated genes in skeletal muscle were ‘cellular component organization’, ‘cellular process’, ‘establishment of localization’, ‘localization’, and ‘metabolic process’ (P-value = 4.94E-04, P-value = 9.12E-04, P-value = 7.09E-02, P-value = 6.87E-02, P-value = 5.28E-02, and P-value = 1.89E-2, respectively). ‘Developmental process’ was the most significantly enriched term in skeletal muscle among both up and down-regulated genes. However, no terms were highlighted as being significantly enriched in blood. We summarized the cellular components and molecular function gene ontology of DUDEGs in RNA-seq data from skeletal muscle and blood of six Thoroughbreds before and after exercise (Table S3 in File S1).

Enriched KEGG pathways analysis using DUDEGs revealed that up-regulated genes in skeletal muscle and blood are associated with exercise-induced stress. The most significantly enriched terms in skeletal muscle were ‘p53 signaling pathway’, ‘regulation of actin cytoskeleton’, ‘JAK-STAT signaling pathway’, ‘MAPK signaling pathway’, ‘cell adhesion molecules’, ‘cytokine-cytokine receptor interaction’, ‘bladder cancer’, and ‘pathways in cancer’. In addition, the two terms ‘colorectal cancer’ and ‘biosynthesis of unsaturated fatty acids’ were significantly enriched in blood (Table 1).

**Table 1:** Enriched KEGG pathways associated with DEGs in two tissue such as skeletal muscle and blood.

Highest KEGG	Higher KEGG	KEGG	Blood		Muscle	
			UP	DOWN	UP	DOWN
Cellular Processes	Cell growth and death	Cell cycle				V
		p53 signaling pathway			V	
	Cell motility	Regulation of actin cytoskeleton			V	
Environmental Information Processing	Membrane transport	ABC transporters				V
	Signal transduction	Jak-STAT signaling pathway			V	
		MAPK signaling pathway			V	
		Notch signaling pathway				V
		Phosphatidylinositol signaling system				V
	Signaling molecules and interaction	Cell adhesion molecules (CAMs)			V	
		Cytokine-cytokine receptor interaction			V	
Genetic Information Processing	Folding, sorting and degradation	Ubiquitin mediated proteolysis				V
	Replication and repair	Non-homologous end-joining				V
Human Diseases	Cancers	Colorectal cancer	V			
		Bladder cancer		V	V	
		Pathways in cancer			V	
		Small cell lung cancer		V		
Metabolism	Carbohydrate metabolism	Butanoate metabolism				V
		Inositol phosphate metabolism				V
	Glycan biosynthesis and metabolism	Glycosylphosphatidylinositol(GPI)-anchor biosynthesis		V		V
	Lipid metabolism	Biosynthesis of unsaturated fatty acids	V			
Organismal Systems	Immune system	B cell receptor signaling pathway			V	
		Hematopoietic cell lineage			V	
		Natural killer cell mediated cytotoxicity			V	
		Toll-like receptor signaling pathway			V	
		T cell receptor signaling pathway			V	

For each set of up-regulated and down-regulated. DEG in skeletal muscle and blood, a KEGG pathway enrichment analysis was performed. Starting from the right, the table shows: tissue type, status of regulation, KEGG pathway terms, higher-level KEGG pathway terms, and the highest level of KEGG pathway terms.

### Integration of DUDEG and selected gene associated with nucleotide diversity, F_ST_ and Cross Population Extended Haplotype Homozygosity (XP-EHH)

The genes under selection were further investigated using nucleotide diversity, F_ST_ and XP-EHH from WGS of Thoroughbred and Jeju pony (skeletal muscle = 1,033 and blood = 567). We found 12 genes (*ZNF592, CD58, C1orf162, USP37, FOXM1, TIMELESS, TRMT1, CALR, ASNA1, EIF4A3, SYNRG* and *FADS1*) in blood and 14 genes (*HERC2, CHD9, DDX28, CAPZA1, TSEN15, CHMP4C, FOXO3, PLD2, ANKRD13D, UNKL, CBFA2T2, NECAB3, SLC25A29* and *FBLN1*) in skeletal muscle that were both identified as DUDEGs and implicated in F_ST_ analysis as a selected gene (Table S4 in File S1). The F_ST_ distribution histogram for the pair of horse breeds is shown in Figure S7 in File S1.

**Figure 3:**
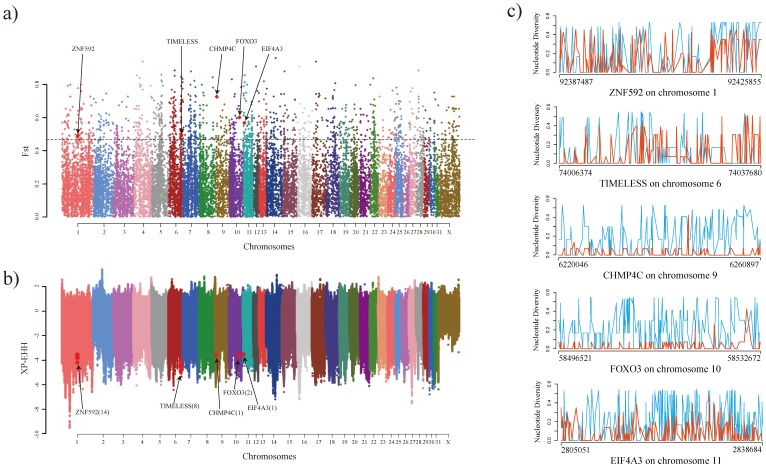
Signatures of correlation between DEGs from Thoroughbred RNA-seq and selected genes associated with nucleotide diversity, F_ST_ and XP-EHH from Thoroughbred and Jeju pony DNA sequence. a) Manhattan plot of F_ST_ (Dotted line = cut-off value of the top 5% with empirical p-values of <0.05. Red point = common genes between the DEGs, Thoroughbred selected genes associated with F_ST_ and XP-EHH). b) Manhattan plot of XP-EHH value (Red point = Common genes between the DEGs and Thoroughbred selected genes associated with F_ST_ and XP-EHH). c) Nucleotide diversity line plot of five common genes (sky blue color line = Jeju pony, orange color line = Thoroughbred).

Between DUDEGs and XP-EHH (selected genes in the Thoroughbred), we found 11 (*ANK3, ZNF592, TOR1AIP1, TIMELESS, INSR, MED15, ZNF567, EIF4A3, EXOC6B, PPP4R2* and *DYRK1A*) and 48 common genes (*URB2, NEO1, CORO2B, CPE, FUK, SYCE1L, TEC, RELL1, ZNF775, RGS4, FAM46C, PTGFRN, DENND2C, CTTNBP2NL, VAV1, BARX2, RAB31, VPS4B, CHMP4C, DEPTOR, ATG5, FOXO3, OSBPL7, PEX12, PIK3R5, PITX1, MARCH3, LMNB1, ST8SIA4, RASGRF2, ARSB, INPP4A, RNF144A, PPP4R2, FRMD4B, SPATA13, SLC7A1, CAB39L, B3GALT1, MECOM, PARP14, NPR3, TGM3, DHX35, AUH, C14orf102, COL27A1* and *HLCS*) in blood and skeletal muscle, respectively (Table S5 in File S1). Among them, three genes, *TIMELESS, EIF4A3* and *ZNF592*, in blood, and two genes, *CHMP4C* and *FOXO3*, in skeletal muscle were shown to be significant in all three analyses (DUDEG, F_ST_, and XP-EHH). In comparison to Jeju pony, the Thoroughbred showed relatively low levels of nucleotide diversity at three out of the five identified genes (Table 2 and Figure 3).

**Table 2:** Co-matching genes between the DEGs, selected genes associated with F_st_ (F_st_ cut-off value top 5% with empirical p-value<0.05) and Thoroughbred selected genes associated with XP-EHH (XP-EHH cut-off value empirical p-value<0.01 and XP-EHH value <−3.51551 significant SNPs).

Sample Tissue	Ens ID	CHR	Start	End	DEG logFC	DEG P-value	DEG FDR	Gene symbol	Reynolds	Fst	SNP region	XP-EHH	XP-EHH P-value
Blood	ENSECAG00000015925	11	2814986	2828951	−17.23	1.81E-34	2.94E-31	*EIF4A3* (eukaryotic translation initiation factor 4A3)	0.83481	0.56604	2821795	−3.52526	9.73E-03
	ENSECAG00000016283	1	92397477	92416004	−14.5988	2.23E-26	5.06E-24	*ZNF592* (zinc finger protein 592)	0.67059	0.48859	92387687	−3.55925	8.83E-03
											92397597	−3.78677	4.48E-03
											92398170	−3.74169	5.15E-03
											92398472	−3.78551	4.50E-03
											92398776	−3.83657	3.84E-03
											92398791	−4.18477	1.23E-03
											92398828	−3.56243	8.74E-03
											92398986	−3.5556	8.92E-03
											92399035	−3.76415	4.81E-03
											92402102	−3.60557	7.72E-03
											92402157	−3.58028	8.31E-03
											92402965	−3.58305	8.24E-03
											92403116	−3.61357	7.54E-03
											92403392	−3.55258	9.00E-03
	ENSECAG00000002892	6	74016170	74027809	9.80616	1.34E-12	6.75E-11	*TIMELESS* (timeless circadian clock)	0.67349	0.49007	74019099	−4.02647	2.09E-03
											74019114	−4.05026	1.93E-03
											74019135	−4.28442	8.68E-04
											74019141	−4.56193	3.15E-04
											74019149	−4.42328	5.27E-04
											74019152	−4.43649	5.02E-04
											74019153	−4.81799	1.16E-04
											74019241	−4.96438	6.40E-05
Muscle	ENSECAG00000022647	9	6229159	6251198	12.97629	5.96E-22	7.12E-20	*CHMP4C* (charged multivesicular body protein 4C)	1.29183	0.72523	6257522	−3.56699	8.63E-03
	ENSECAG00000024499	10	58506453	58523225	1.941151	0.001093	0.008744	*FOXO3* (forkhead box O3)	0.91477	0.59939	58521033	−3.5866	8.15E-03
											58521300	−3.67329	6.32E-03

## Discussion

### Differences in result between reference-based and *de novo*-based assembly and analysis

Up to now, many studies of RNA-seq analyses have used reference-based analysis (RBA) when a reference genome for the species is available [27], [46], [47]. When a species does not have a reference genome, RBA using the reference genome of a closely related species or *de novo*-based analysis (DBA) is used. Several studies of RNA-seq analyses have used the align-then-assemble combined method (align-then-assemble and assemble-then-align) [24]. DBA used in align-then-assemble method assembles the unmapped sequence reads after RBA, which supplements the inherent weakness of RBA. However, we suggest that even if a species has a reference genome, DBA used in assemble-then-align is necessary for assembly of the total sequence reads including the unmapped sequence reads (Table S6 and S7 in File S1). In this study, we found a significant difference in the number of transcript and differentially expressed genes (DEGs) identified by RBA and DBA. Also, a greater number of unique transcripts were identified by DBA than in RBA (Figure 1a and Figure S1a in File S1). This implies that the horse RNA-seq data in this study includes new transcripts and new transcript structures that are not included in the horse reference genome. Additionally, we found *de novo* unique differentially expressed genes (DUDEGs) which cannot be attained from RBA (Figure 1c and Figure S1c in File S1). Credibility of DBA in RNA-seq has been proven in numerous methods and protocol papers [17], [25], [48] and in this study. Both multidimensional scaling (MDS) plot (Figure 1b, Figure S1b and Figure S2 in File S1) and expression patterns of common DEGs did not show differences between the results of RBA and DBA. However, the intensity of the expression was different because the newly assembled transcriptome reference represents the individual transcriptome made using de Bruijn graph assemblers during DBA [48] (Figure 1d and Figure S1d in File S1).

RNA-Seq can reveal sequence variations such as SNP in genes [49] as is possible with whole genome sequencing (WGS). Transcribed SNPs in RNA-seq are needed for accurate measurement of allele-specific expression [50], [51] and detection of novel SNPs. [52]. Hence, we compared the number and rate of SNPs identified from the two NGS methods and found differences between the type of references and NGS methods. In summary, we detected more SNPs in *de novo* assembly of RNA-seq than in the reference genome assembly of cDNA (Table S2 in File S1).

### Identification and Functional annotation of unique DEGs identified by *de novo* base assembly

We identified DUDEGs to ascertain the important function of DEGs, which cannot be attained from RBA. In the highest biological process gene ontology (BP-GO) of DUDEG result, immune system process had the most significant P-value (P-value = 2.23E-13) in up-regulation of skeletal muscle (Figure 2). Response to stimulus had the second most significant P-value (P-value = 5.16E-08), which is related with immune response caused by exercise-induced stress [53]. Exercise-induced stress is closely related with the regulation of immune response [6], [27]. Over-exercise in horses has shown an increase in the expression of alpha-1-antitrypsin protein, which plays an important role in protection of cells from inflammatory enzymes released from neutrophils [54]. Exercise-induced reactive oxygen species was also related with the regulation of immune responses, and caused the inflammatory responses from muscle damage [55], [56]. In the KEGG pathways result (Table 1), the JAK-STAT signaling pathway and MAPK signaling pathway were also up-regulated in the skeletal muscle. The JAK-STAT signaling is a key pathway in myoblast proliferation [57] and plays a major role in inflammatory and immune responses [58]. The MAPK signaling pathway is implicated in inflammation and carbohydrate metabolism [59]. Death, Related with apoptosis of skeletal muscle caused by over-exercise also had a significant P-value (P-value = 1.75E-06). This was supported by the KEGG pathways results, which showed that the p53 signaling pathway and regulation of actin cytoskeleton were up-regulated. P53 protein has an important role in apoptosis of skeletal muscle and actin cytoskeleton, and is also a key pathway in regulation of apoptosis pathways [60], [61]. However, we did not find immune responses and apoptosis related with BP-GO and KEGG pathway in blood.

### Integration of conceptually new DEGs: DUDEGs and selected gene associated with nucleotide diversity, F_ST_ and Cross Population Extended Haplotype Homozygosity (XP-EHH)

In order to investigate the evolutionary history of domestication in relation to different experimental conditions, we approached the identification of DEGs with a new concept. The conceptually new DEGs were attained by screening for genes in common between DUDEGs from DBA in RNA-seq and selected genes identified by evolutionary statistics, such as nucleotide diversity, F_ST_ and XP-EHH from RBA in WGS.

This comparison highlighted three genes (*EIF4A3*, *ZNF592 and TIMELESS*) in blood and two genes (*CHMP4C*, and *FOXO3*) in skeletal muscle as being in common between DUDEGs, F_ST_ and XP-EHH. These five genes are not only DUDEGs in six Thoroughbreds, before and after exercise, but also selected genes (F_ST_ (empirical p-value<0.01) and XP-EHH (value<0 and p-value<0.01)). A pairwise test, XP-EHH, of the Thoroughbred and Jeju domestic pony populations was used to identify selective sweep regions between the two populations. As we are interested in locating selective sweep regions representing adaptation in Thoroughbreds, a cutoff of XP-EHH value<0 was used. If the XP-EHH value>0 is used, then the identified selective sweep region would correspond to the adaptations in Jeju domestic pony.

The five genes were conceptually new DEGs, and were related to the evolution of exercise response during the domestication process of Thoroughbred. Among them, three genes, *CHMP4C, EIF4A3* and *FOXO3*, showed relatively low levels of nucleotide diversity compared to that of the Jeju pony (Table 2 and Figure 3). This suggests that these three genes have been more strongly selected for in Thoroughbred than in Jeju pony. *EIF4A3* was mostly expressed in megakaryocytes, platelets and red blood cell. *EIF4A3*, an mRNA-localization protein in mammals, controls the synaptic strength, neuronal protein expression, and in megakaryocytes and platelets act as mRNA sorting machinery [62]–[64]. In a previous study, it was shown that over-exercise activates and increases platelets [65]. Although, it has an important role in blood post-exercise in Thoroughbreds, *EIF4A3* expression was up-regulated in our results. *CHMP4C* is a *p53*-regulated gene and plays an important role in exosome production [66]. The importance of p53 in apoptosis of skeletal muscle was implicated in a previous study, in which p53-null animals showed greater fatigability and less locomotory endurance than wild-type animals [60]. This suggests that p53 is closely related with exercise-induced stress in skeletal muscle. *CHMP4C* expression was down-regulated in our results implying the activation of p53 regulation in Thoroughbred skeletal muscle. *FOXO3*, also known as Forkhead box O3, has a role in triggering apoptosis by down-regulating the *FOXO3* gene. In addition, *FOXO3* causes a loss of muscle mass, and is closely related to PGC1α, ATG4b, ATG12, Beclin1, Gabarapl1, and LC3b. PGC1α, the transcription of atrophy-specific genes, inhibits the activity of the transcription factor *FOXO3*, with protects skeletal muscle from atrophy [67]. In human muscle after ultra-endurance exercise, the expression of several autophagy genes, ATG4b, ATG12, Beclin1, Gabarapl1 and LC3b, were increased [68], [69]. For this reason, *FOXO3* was also closely related to exercise in Thoroughbred skeletal muscle.

Based on these results, *EIF4A3, CHMP4C* and *FOXO3* are conceptually new DEGs involved in exercise response that have been selected for during the domestication history of the Thoroughbred that cannot be acquired by RBA.

## Supporting Information

File S1 Contains the following. Table S1, List of unique DEGs in skeletal muscle and blood in six Thoroughbred horses before and after exercise RNA-seq data by de novo assembly (FDR<0.01). Table S2, The number and rate of SNPs from different next-generation sequencing method (DNA and RNA sequencing) and different reference genome assembly in each Thoroughbred horse sample (F1, F2 and F3 = male, S3 = female). Table S3, GO terms of cellular components and molecular function of two tissues specific DEGs between before the exercise and after exercise in horses. Table S4, Common genes between DEGs and selected genes associated with F_ST_ (F_ST_ cut-off value top 5% with empirical p-value<0.05). Table S5, Common genes between DEGs and selected genes associated with XP-EHH: XP-EHH cut-off value empirical p-value<0.01 and XP-EHH value <−3.51551 significant SNPs in Thoroughbred were selected and >1.73481 significant SNPs in Jeju domestic pony were selected. Table S6, List of basic stats such as the number of transcripts, components, and contig N50 value in RNA-seq whole reads and unmapped reads by trinity *de novo* assembly. Table S7, Number of annotated transcripts from RNA-seq unmapped reads by trinity *de novo* assembly. The number in the parentheses is the number of transcripts that were not included in the results of the reference-based analysis. Table S8, Basic information of 4 horses re-sequencing data. Table S9, RT-PCR primer information such as the gene symbol, direction and sequence. Figure S1, Summary of comparative analysis between *de novo* assemble and reference genome assemble from blood in six Thoroughbred horses before and after exercise RNA-seq data (Total 12 samples). a) The number of common transcripts of 12 samples between de novo assemble and reference genome assemble b) MDS plot of six Thoroughbred horses before and after exercise using de novo assemble. c) The number of DEGs between de novo assemble and reference genome assemble. d) Heat-map visualization of common DEGs between de novo assemble and reference genome assemble: rows represent DEGs from blood and columns represent assemble method from 6 horse samples (*First ‘B’ is for Blood and ‘M’ is for muscle. ‘F1’, ‘F2’, ‘F3’ and ‘S3’ are horse samples. Last ‘B’ is for ‘before exercise’ and ‘P’ is for ‘after exercise’). Figure S2, MDS plot of six Thoroughbred horses before and after exercise using reference genome assemble in RNA-seq. a) MDS plot of blood tissue in six Thoroughbred horse before and after exercise. b) MDS plot of skeletal muscle tissue in six Thoroughbred horse before and after exercise. (*First ‘B’ is for Blood and ‘M’ is for muscle. ‘F1’, ‘F2’, ‘F3’ and ‘S3’ are horse samples. Last ‘B’ is for ‘before exercise’ and ‘P’ is for ‘after exercise’). Figure S3, Hierarchical clustering of biological process GO terms associated with up-regulated DEGs in blood. The gene list of each GO term clustered using DAVID was compared to calculate the distance between the GO terms. For a distance value >0.5, GO terms were re-clustered, and GO term groups are shown as light-blue graduated blocks. The number of genes associated with the re-clustered GO term group is shown on the left side of the block. Figure S4, Hierarchical clustering of biological process GO terms associated with down-regulated DEGs in blood. The gene list of each GO term clustered using DAVID was compared to calculate the distance between the GO terms. For a distance value >0.5, GO terms were re-clustered, and GO term groups are shown as light-blue graduated blocks. The number of genes associated with the re-clustered GO term group is shown on the left side of the block. Figure S5, Hierarchical clustering of biological process GO terms associated with up-regulated DEGs in muscle. The gene list of each GO term clustered using DAVID was compared to calculate the distance between the GO terms. For a distance value >0.5, GO terms were re-clustered, and GO term groups are shown as light-blue graduated blocks. The number of genes associated with the re-clustered GO term group is shown on the left side of the block. Figure S6, Hierarchical clustering of biological process GO terms associated with down-regulated DEGs in muscle. The gene list of each GO term clustered using DAVID was compared to calculate the distance between the GO terms. For a distance value >0.5, GO terms were re-clustered, and GO term groups are shown as light-blue graduated blocks. The number of genes associated with the re-clustered GO term group is shown on the left side of the block. Figure S7, Histogram of conventional F_ST_ frequency between Thoroughbred and jeju pony. x-axis is conventional F_ST_ value, y-axis is gene frequency. Figure S8, qRT-PCR validation of *de novo* unique differentially expressed genes (DUDEGs) identified from the RNA-seq data set of Thoroughbred horses before and after exercise: a) RT-PCR of six DUDEG in horses before exercise and after exercise. b) qRT-PCR results depicted as Ct value was calculated using 2-^ΔΔCt^ method. *: p-value<0.05. **: p-value<0.01. #: The expression patters of genes supported the result of our analysis.
(ZIP)
