# Supplementary material for: Investigation of De Novo Unique Differentially Expressed Genes Related to Evolution in Exercise Response during Domestication in Thoroughbred Race Horses
Source: PLoS One. 2014 Mar 21;9(3):e91418. doi: 10.1371/journal.pone.0091418 (PMC3962374; doi:10.1371/journal.pone.0091418)
Supplement: File S1 — Contains the following. Table S1, List of unique DEGs in skeletal muscle and blood in six Thoroughbred horses before and after exercise RNA-seq data by de novo assembly (FDR<0.01). Table S2, The number and rate of SNPs from different next-generation sequencing method (DNA and RNA sequencing) and different reference genome assembly in each Thoroughbred horse sample (F1, F2 and F3 = male, S3 = female). Table S3, GO terms of cellular components and molecular function of two tissues specific DEGs between before the exercise and after exercise in horses. Table S4, Common genes between DEGs and selected genes associated with FST (FST cut-off value top 5% with empirical p-value<0.05). Table S5, Common genes between DEGs and selected genes associated with XP-EHH: XP-EHH cut-off value empirical p-value<0.01 and XP-EHH value <−3.51551 significant SNPs in Thoroughbred were selected and >1.73481 significant SNPs in Jeju domestic pony were selected. Table S6, List of basic stats such as the number of transcripts, components, and contig N50 value in RNA-seq whole reads and unmapped reads by trinity de novo assembly. Table S7, Number of annotated transcripts from RNA-seq unmapped reads by trinity de novo assembly. The number in the parentheses is the number of transcripts that were not included in the results of the reference-based analysis. Table S8, Basic information of 4 horses re-sequencing data. Table S9, RT-PCR primer information such as the gene symbol, direction and sequence. Figure S1, Summary of comparative analysis between de novo assemble and reference genome assemble from blood in six Thoroughbred horses before and after exercise RNA-seq data (Total 12 samples). a) The number of common transcripts of 12 samples between de novo assemble and reference genome assemble b) MDS plot of six Thoroughbred horses before and after exercise using de novo assemble. c) The number of DEGs between de novo assemble and reference genome assemble. d) Heat-map visualization of common DEG [file pone.0091418.s001.zip › 3.SupportingInformation_FileS1.docx]

| **SUPPORTING INFOMATION** |
| --- |

**Investigation of *de novo* unique differential expressed genes relate to exercise domestication in Thoroughbred race horse**

Woncheoul Park^1^, Jaemin Kim^2^, Hyeonjeong Kim^3^, JaeYoung Choi^4^, Jeong-Woong Park^4^, Hyun-Woo Cho^4^, Byeong-Woo Kim^4^, Myung Hum Park^5^, Teak-Soon Shin^4^, Seong-Keun Cho^4^, Jun-Kyu Park^6^, Heebal Kim^1,3^, Jae Yeon Hwang^1^, Chang-Kyu Lee^1^, Hak-Kyo Lee^7^, Seoae Cho^3*^, Byung-Wook Cho^4*^

^1^Department of Agricultural Biotechnology and Research Institute for Agriculture and Life Sciences, Seoul National University, Seoul, Republic of Korea

^2^Interdisciplinary Program in Bioinformatics, Seoul National University, Seoul, Republic of Korea

^3^C&K genomics, Seoul National University, Seoul, Republic of Korea

^4^Department of Animal Science, College of Life Sciences, Pusan National University, Miryang, Republic of Korea

^5^TNT Research, Anyang, Republic of Korea

^6^Leaders in Industry-university Cooperation, Pusan National University, Miryang, Republic of Korea

^7^Genomic Informatics Center, Hankyong National University, Anseong, Republic of Korea

| **TABLE OF CONTENTS** |
| --- |

1. **Supplementary Methods…………………………………………………p.3**
2. **Supplementary Tables…………………………………………………p.6**
   1. **Table S1. ………………… ………………………………………p.6**
   2. **Table S2. …………………………………………………………p.48**
   3. **Table S3. …………………………………………………………p.49**
   4. **Table S4. …………………………………………………………p.56**
   5. **Table S5. …………………………………………………………p.58**
   6. **Table S6. …………………………………………………………p.62**
   7. **Table S7. …………………………………………………………p.64**
   8. **Table S8. …………………………………………………………p.65**
   9. **Table S9. …………………………………………………………p.66**
3. **Supplementary Figures…………………………………………………p.67**
   1. **Figure S1. …………………………………………………………p.67**
   2. **Figure S2. …………………………………………………………p.68**
   3. **Figure S3. …………………………………………………………p.69**
   4. **Figure S4. …………………………………………………………p.70**
   5. **Figure S5. …………………………………………………………p.71**
   6. **Figure S6. …………………………………………………………p.72**
   7. **Figure S7. …………………………………………………………p.72**
   8. **Figure S8. …………………………………………………………p.73**

***Supplementary Methods***

**Analysis of horses RNA-seq data**

1. *De novo*-base analysis

we used Trinity software package tool that is represents a novel method for the efficient and robust *de novo* reconstruction of transcriptomes from RNA-seq data and developed at the Broad Institute and the Hebrew University of Jerusalem. Moreover. Moreover, we used a different set of samples for the three different goals:

1. To detect DEG through RNA-seq *de novo*-base analysis: we obtained 24 reference fasta file (Trinity.fasta) provided by Trinity for each sample
2. To detect SNPs through RNA-seq *de novo* -base analysis: four reference fasta file per individual by combining the samples for each individual
3. To compare the performance of de novo-base analysis for the remaining unmapped reads after de novo-base analysis and reference base-analysis using the entire RNA-seq reads: same as number 1, 24 reference fasta file were obtained

After the Trinity assembly, we took the results of the trinity tools which are the components IDs and then used Blastall tool to change then into reference transcript ID. In this process,–e (z-value cutoff) 0.00001 and–p (Search a nucleotide database using a nucleotide query) blastn was used. After Blastall, to obtain more accurate transcripts ID, additional filtering steps were used:

- 1. Identity is the highest and E-value (<0.00001) is lowest
  2. 2. Alignment length coverage > 80%” (Supporting Information page 3, line 3)

2. Reference-base analysis

We processed 90 paired-end RNA-seq reads that carried out based on Illumina HiSeq2000 protocols. Twenty-four sets of transcriptome data was generated for muscle and blood from 6 horses both before and after exercise. Read were aligned to the reference genome (<http://hgdownload.cse.ucsc.edu/downloads.html#horse>) with TopHat (ver.1.4.1) using default setting

1. Genotype calling and SNP calling (*de-novo* vs reference)

We processed paired-end RNA-seq reads. Read were aligned to the reference genome (<ftp://ftp.ensembl.org/pub/release-73/fasta/equus_caballus/dna/>), transcriptome (<ftp://ftp.ensembl.org/pub/release-73/fasta/equus_caballus/cdna/>) and output of Trinity (such as Trinity.fasta) with Bowtie2 (ver.2.1.0) using default. Substitution calls were made with GATK UnifiedGenotyper and the variants were discarded if (1) quality score is less than 30, (2) SNPs existed in detecting InDels, (3) the number or proportion of reads which have mapping quality score 0 are bigger than 4 or 10% (4) Quality by Depth value (QD) value from GATK is less than 5, (5) Fisher strand bias value (FS) from GATK is bigger than 200, (6) the number of alternative allele was bigger than one (multi-allele type).

**Analysis of horses whole genome re-sequencing data**

1. Genotype calling and SNP calling

we processed paired-end sequence reads (~10X coverage of illumina’s HiSeq2000). Reads were aligned to the reference genome (<ftp://ftp.ensembl.org/pub/release-73/fasta/equus_caballus/dna/>) and transcriptome (<ftp://ftp.ensembl.org/pub/release-73/fasta/equus_caballus/cdna/>) with Burrows-Wheeler Aligner (BWA: ver.0.6.1) using default (Supplementary table 8). Substitution calls were made with GATK UnifiedGenotyper and the variants were discarded if (1) quality score is less than 30, (2) SNPs existed in detecting InDels, (3) the number or proportion of reads which have mapping quality score 0 are bigger than 4 or 10% (4) Quality by Depth value (QD) value from GATK is less than 5, (5) Fisher strand bias value (FS) from GATK is bigger than 200, (6) the number of alternative allele was bigger than one (multi-allele type). And we used default option in BEAGLE.

1. Estimation of Nucleotide diversity, F_ST_ and Cross Population Extended Haplotype Homozygosity (XP-EHH) value

Levels of nucleotide diversity were calculated by VCFtools using --site-pi option, because it does this on a per-site basis.

***Supplementary Tables***

**Table S1.** List of unique DEGs in skeletal muscle and blood in six Thoroughbred horses before and after exercise RNA-seq data by *de novo* assembly (FDR<0.01).

(a) Skeletal muscle

| Ens id | logFC | PValue | FDR |
| --- | --- | --- | --- |
| ENSECAG00000013712 | -18.4969 | 1.01E-37 | 2.6E-34 |
| ENSECAG00000008198 | -17.5986 | 9.58E-35 | 1.27E-31 |
| ENSECAG00000014980 | -17.5894 | 1.03E-34 | 1.27E-31 |
| ENSECAG00000024303 | -17.5875 | 1.08E-34 | 1.27E-31 |
| ENSECAG00000014745 | -17.5579 | 1.47E-34 | 1.51E-31 |
| ENSECAG00000013163 | -17.5533 | 1.52E-34 | 1.51E-31 |
| ENSECAG00000011147 | -16.0042 | 1.87E-30 | 1.42E-27 |
| ENSECAG00000022125 | -15.0884 | 8.21E-28 | 3.92E-25 |
| ENSECAG00000008837 | -14.6914 | 3.18E-27 | 1.32E-24 |
| ENSECAG00000020356 | -14.4215 | 5.31E-26 | 1.71E-23 |
| ENSECAG00000000477 | -14.2891 | 5.97E-25 | 1.37E-22 |
| ENSECAG00000010457 | -14.0586 | 4.46E-25 | 1.08E-22 |
| ENSECAG00000021743 | -13.9434 | 6.28E-24 | 1.23E-21 |
| ENSECAG00000022158 | -13.8808 | 5.79E-25 | 1.36E-22 |
| ENSECAG00000015568 | -13.7536 | 2.25E-23 | 3.67E-21 |
| ENSECAG00000021915 | -13.6996 | 2.64E-25 | 6.8E-23 |
| ENSECAG00000019475 | -13.6717 | 1.68E-23 | 2.81E-21 |
| ENSECAG00000009942 | -13.4972 | 3.22E-23 | 4.95E-21 |
| ENSECAG00000022769 | -13.4755 | 1.99E-23 | 3.3E-21 |
| ENSECAG00000005963 | -13.4546 | 9.97E-23 | 1.37E-20 |
| ENSECAG00000000610 | -13.4392 | 8.95E-23 | 1.26E-20 |
| ENSECAG00000000230 | -13.4189 | 3.97E-23 | 6.03E-21 |
| ENSECAG00000014581 | -13.4003 | 8.78E-23 | 1.25E-20 |
| ENSECAG00000008779 | -13.3525 | 1.37E-22 | 1.79E-20 |
| ENSECAG00000008314 | -13.3261 | 7.27E-24 | 1.33E-21 |
| ENSECAG00000014329 | -13.2564 | 9.41E-23 | 1.31E-20 |
| ENSECAG00000013183 | -13.2172 | 4.04E-22 | 5.01E-20 |
| ENSECAG00000021585 | -13.2068 | 2.59E-22 | 3.25E-20 |
| ENSECAG00000013827 | -13.1103 | 1.82E-21 | 2.02E-19 |
| ENSECAG00000002801 | -13.092 | 1.37E-22 | 1.79E-20 |
| ENSECAG00000017830 | -13.0758 | 1.48E-21 | 1.69E-19 |
| ENSECAG00000011954 | -13.0729 | 4.28E-22 | 5.26E-20 |
| ENSECAG00000009220 | -13.0167 | 1.56E-21 | 1.76E-19 |
| ENSECAG00000002450 | -12.929 | 2.07E-21 | 2.28E-19 |
| ENSECAG00000004033 | -12.9175 | 2.47E-21 | 2.7E-19 |
| ENSECAG00000011655 | -12.8665 | 1.28E-21 | 1.47E-19 |
| ENSECAG00000002126 | -12.7901 | 4.25E-21 | 4.49E-19 |
| ENSECAG00000021511 | -12.7721 | 9.57E-21 | 9.28E-19 |
| ENSECAG00000011989 | -12.7134 | 1.08E-21 | 1.26E-19 |
| ENSECAG00000013143 | -12.6504 | 2.3E-20 | 2.08E-18 |
| ENSECAG00000004823 | -12.5274 | 1.02E-20 | 9.79E-19 |
| ENSECAG00000013043 | -12.4954 | 4.44E-20 | 3.87E-18 |
| ENSECAG00000008496 | -12.3527 | 4.74E-20 | 4.1E-18 |
| ENSECAG00000018708 | -12.3047 | 1.83E-19 | 1.43E-17 |
| ENSECAG00000017097 | -12.2491 | 1.54E-19 | 1.21E-17 |
| ENSECAG00000013834 | -12.2486 | 3.75E-19 | 2.71E-17 |
| ENSECAG00000001052 | -12.1996 | 2.35E-19 | 1.75E-17 |
| ENSECAG00000017762 | -12.1415 | 1.36E-18 | 8.75E-17 |
| ENSECAG00000006911 | -12.1225 | 5.34E-19 | 3.65E-17 |
| ENSECAG00000016410 | -12.0774 | 1.61E-18 | 1.02E-16 |
| ENSECAG00000021941 | -12.05 | 1.22E-18 | 7.99E-17 |
| ENSECAG00000019949 | -12.0327 | 2.17E-18 | 1.35E-16 |
| ENSECAG00000002394 | -11.9995 | 2.27E-18 | 1.39E-16 |
| ENSECAG00000007787 | -11.9519 | 2.12E-19 | 1.6E-17 |
| ENSECAG00000012146 | -11.8921 | 6.23E-18 | 3.45E-16 |
| ENSECAG00000016723 | -11.8556 | 5.61E-18 | 3.16E-16 |
| ENSECAG00000023929 | -11.8512 | 2.41E-19 | 1.78E-17 |
| ENSECAG00000002713 | -11.8383 | 5.41E-18 | 3.08E-16 |
| ENSECAG00000026980 | -11.723 | 2.54E-19 | 1.86E-17 |
| ENSECAG00000005311 | -11.6964 | 8.95E-18 | 4.77E-16 |
| ENSECAG00000005452 | -11.6856 | 6.54E-18 | 3.6E-16 |
| ENSECAG00000022702 | -11.6452 | 4.35E-18 | 2.51E-16 |
| ENSECAG00000001707 | -11.4818 | 5.4E-17 | 2.6E-15 |
| ENSECAG00000003858 | -11.4671 | 1.37E-17 | 7.15E-16 |
| ENSECAG00000011802 | -11.378 | 2.08E-17 | 1.06E-15 |
| ENSECAG00000022775 | -11.3128 | 2.47E-18 | 1.5E-16 |
| ENSECAG00000017726 | -11.3 | 4.87E-17 | 2.36E-15 |
| ENSECAG00000014922 | -11.2877 | 4.73E-17 | 2.3E-15 |
| ENSECAG00000020083 | -11.1557 | 3.99E-16 | 1.7E-14 |
| ENSECAG00000003786 | -11.12 | 4.96E-16 | 2.06E-14 |
| ENSECAG00000018771 | -11.0834 | 1.58E-16 | 7.18E-15 |
| ENSECAG00000007104 | -11.0253 | 2.52E-16 | 1.12E-14 |
| ENSECAG00000014689 | -10.9847 | 2.73E-15 | 1.04E-13 |
| ENSECAG00000021401 | -10.974 | 1.25E-15 | 4.93E-14 |
| ENSECAG00000012626 | -10.9415 | 1.71E-15 | 6.59E-14 |
| ENSECAG00000024390 | -10.9303 | 4.08E-15 | 1.51E-13 |
| ENSECAG00000019815 | -10.7976 | 4.37E-15 | 1.61E-13 |
| ENSECAG00000001525 | -10.7901 | 2.75E-16 | 1.21E-14 |
| ENSECAG00000005457 | -10.7409 | 1.71E-15 | 6.59E-14 |
| ENSECAG00000016220 | -10.6046 | 1.21E-14 | 4.12E-13 |
| ENSECAG00000002375 | -10.5102 | 7.53E-15 | 2.68E-13 |
| ENSECAG00000004489 | -10.5059 | 3.85E-14 | 1.23E-12 |
| ENSECAG00000002032 | -10.4931 | 7.86E-14 | 2.4E-12 |
| ENSECAG00000020733 | -10.4584 | 3.75E-14 | 1.21E-12 |
| ENSECAG00000005834 | -10.3613 | 2.6E-14 | 8.58E-13 |
| ENSECAG00000018293 | -10.3583 | 9.39E-15 | 3.28E-13 |
| ENSECAG00000002440 | -10.3207 | 1.22E-13 | 3.67E-12 |
| ENSECAG00000006261 | -10.3151 | 1.07E-13 | 3.25E-12 |
| ENSECAG00000021875 | -10.2456 | 3.63E-13 | 1.03E-11 |
| ENSECAG00000009821 | -10.1779 | 5.43E-13 | 1.49E-11 |
| ENSECAG00000015949 | -10.1235 | 3.14E-13 | 9.06E-12 |
| ENSECAG00000020600 | -10.0784 | 2.27E-14 | 7.56E-13 |
| ENSECAG00000007014 | -10.0727 | 4.04E-13 | 1.15E-11 |
| ENSECAG00000008633 | -10.0684 | 4.51E-13 | 1.26E-11 |
| ENSECAG00000006209 | -10.0495 | 9.69E-13 | 2.59E-11 |
| ENSECAG00000010881 | -10.0141 | 1.58E-12 | 4.1E-11 |
| ENSECAG00000023900 | -9.98997 | 6.32E-13 | 1.72E-11 |
| ENSECAG00000006687 | -9.96543 | 1.11E-13 | 3.34E-12 |
| ENSECAG00000003735 | -9.78092 | 1.18E-12 | 3.11E-11 |
| ENSECAG00000017106 | -9.66382 | 4.34E-12 | 1.07E-10 |
| ENSECAG00000010973 | -9.54468 | 5.75E-12 | 1.39E-10 |
| ENSECAG00000008257 | -9.4148 | 1.93E-11 | 4.46E-10 |
| ENSECAG00000008989 | -9.40571 | 4.76E-12 | 1.16E-10 |
| ENSECAG00000005463 | -9.36877 | 4E-11 | 8.94E-10 |
| ENSECAG00000017070 | -9.2854 | 2.32E-12 | 5.91E-11 |
| ENSECAG00000012158 | -9.26203 | 5.69E-11 | 1.26E-09 |
| ENSECAG00000024528 | -9.14674 | 1.46E-10 | 3.08E-09 |
| ENSECAG00000006814 | -9.13985 | 1.37E-10 | 2.9E-09 |
| ENSECAG00000007009 | -9.10243 | 3.35E-10 | 6.94E-09 |
| ENSECAG00000003005 | -8.99745 | 1.67E-10 | 3.51E-09 |
| ENSECAG00000000165 | -8.92716 | 6.05E-10 | 1.22E-08 |
| ENSECAG00000021630 | -8.80347 | 1.58E-09 | 3.07E-08 |
| ENSECAG00000014382 | -8.65797 | 3.87E-10 | 7.94E-09 |
| ENSECAG00000000369 | -8.49612 | 1.23E-08 | 2.19E-07 |
| ENSECAG00000002660 | -7.28399 | 9.74E-16 | 3.87E-14 |
| ENSECAG00000012129 | -6.32515 | 4.6E-11 | 1.02E-09 |
| ENSECAG00000017920 | -6.1276 | 1.11E-12 | 2.93E-11 |
| ENSECAG00000004518 | -6.01009 | 4.09E-11 | 9.13E-10 |
| ENSECAG00000003186 | -5.90387 | 8.95E-13 | 2.41E-11 |
| ENSECAG00000010273 | -5.86795 | 2.33E-12 | 5.93E-11 |
| ENSECAG00000004728 | -5.73656 | 2.92E-12 | 7.35E-11 |
| ENSECAG00000006274 | -5.59771 | 2.03E-11 | 4.67E-10 |
| ENSECAG00000014547 | -5.4822 | 3.2E-12 | 8E-11 |
| ENSECAG00000004427 | -5.47179 | 1.33E-11 | 3.12E-10 |
| ENSECAG00000018346 | -5.42103 | 8.6E-11 | 1.86E-09 |
| ENSECAG00000011395 | -5.33809 | 3.19E-11 | 7.2E-10 |
| ENSECAG00000006502 | -5.31197 | 1.25E-09 | 2.44E-08 |
| ENSECAG00000004714 | -5.17503 | 6.8E-09 | 1.24E-07 |
| ENSECAG00000012737 | -5.13201 | 6.02E-10 | 1.22E-08 |
| ENSECAG00000016578 | -5.08585 | 3.37E-10 | 6.98E-09 |
| ENSECAG00000010021 | -5.08425 | 8.25E-10 | 1.65E-08 |
| ENSECAG00000007594 | -5.08122 | 1.12E-10 | 2.39E-09 |
| ENSECAG00000021287 | -5.07581 | 1.38E-10 | 2.91E-09 |
| ENSECAG00000019052 | -5.0631 | 8.8E-11 | 1.9E-09 |
| ENSECAG00000010581 | -4.99514 | 1.23E-09 | 2.41E-08 |
| ENSECAG00000003321 | -4.92977 | 2.01E-09 | 3.88E-08 |
| ENSECAG00000002837 | -4.87282 | 4.51E-10 | 9.22E-09 |
| ENSECAG00000008753 | -4.86216 | 3.15E-10 | 6.57E-09 |
| ENSECAG00000016659 | -4.85231 | 3.59E-09 | 6.7E-08 |
| ENSECAG00000000256 | -4.84785 | 3.69E-09 | 6.88E-08 |
| ENSECAG00000012201 | -4.82313 | 3.6E-10 | 7.42E-09 |
| ENSECAG00000018989 | -4.82258 | 3.35E-09 | 6.28E-08 |
| ENSECAG00000021911 | -4.81462 | 3.6E-10 | 7.42E-09 |
| ENSECAG00000017973 | -4.79991 | 4.61E-09 | 8.5E-08 |
| ENSECAG00000012798 | -4.77249 | 5.93E-09 | 1.08E-07 |
| ENSECAG00000010337 | -4.76922 | 1.03E-09 | 2.04E-08 |
| ENSECAG00000006794 | -4.74325 | 1.33E-09 | 2.61E-08 |
| ENSECAG00000014206 | -4.6576 | 1.22E-08 | 2.17E-07 |
| ENSECAG00000020135 | -4.64862 | 3.01E-09 | 5.66E-08 |
| ENSECAG00000014172 | -4.63851 | 2.32E-09 | 4.44E-08 |
| ENSECAG00000020492 | -4.62848 | 1.12E-08 | 2.01E-07 |
| ENSECAG00000003444 | -4.61767 | 5.8E-09 | 1.06E-07 |
| ENSECAG00000005449 | -4.60041 | 2.63E-09 | 5.03E-08 |
| ENSECAG00000012694 | -4.5966 | 1.53E-08 | 2.71E-07 |
| ENSECAG00000012281 | -4.56382 | 1.18E-08 | 2.11E-07 |
| ENSECAG00000013033 | -4.53597 | 2.56E-08 | 4.5E-07 |
| ENSECAG00000001452 | -4.53587 | 3.71E-09 | 6.91E-08 |
| ENSECAG00000007846 | -4.52792 | 9.13E-09 | 1.65E-07 |
| ENSECAG00000002902 | -4.48572 | 2.65E-08 | 4.65E-07 |
| ENSECAG00000008460 | -4.48221 | 6.5E-09 | 1.18E-07 |
| ENSECAG00000008547 | -4.44135 | 9.91E-09 | 1.79E-07 |
| ENSECAG00000014733 | -4.37036 | 7.15E-08 | 1.21E-06 |
| ENSECAG00000023906 | -4.36962 | 7.08E-09 | 1.28E-07 |
| ENSECAG00000000465 | -4.35131 | 1.04E-08 | 1.87E-07 |
| ENSECAG00000023328 | -4.33977 | 8.63E-09 | 1.56E-07 |
| ENSECAG00000007429 | -4.33237 | 3.95E-08 | 6.84E-07 |
| ENSECAG00000003548 | -4.33188 | 9.01E-09 | 1.63E-07 |
| ENSECAG00000002643 | -4.30721 | 1.92E-08 | 3.39E-07 |
| ENSECAG00000010709 | -4.2384 | 2.9E-08 | 5.08E-07 |
| ENSECAG00000019017 | -4.23711 | 4.14E-09 | 7.69E-08 |
| ENSECAG00000015639 | -4.21674 | 2.5E-08 | 4.4E-07 |
| ENSECAG00000007432 | -4.20836 | 2.88E-07 | 4.72E-06 |
| ENSECAG00000005584 | -4.18514 | 5.92E-08 | 1.01E-06 |
| ENSECAG00000002439 | -4.18397 | 3.24E-08 | 5.66E-07 |
| ENSECAG00000012084 | -4.18219 | 3.67E-08 | 6.39E-07 |
| ENSECAG00000004601 | -3.96993 | 4.73E-08 | 8.13E-07 |
| ENSECAG00000015751 | -3.9646 | 8.86E-06 | 0.000124 |
| ENSECAG00000003346 | -3.94819 | 3.02E-07 | 4.93E-06 |
| ENSECAG00000017736 | -3.91305 | 2.12E-07 | 3.49E-06 |
| ENSECAG00000004812 | -3.89874 | 1.92E-07 | 3.18E-06 |
| ENSECAG00000001465 | -3.89007 | 4.25E-07 | 6.87E-06 |
| ENSECAG00000024415 | -3.87162 | 1.34E-07 | 2.24E-06 |
| ENSECAG00000001771 | -3.86877 | 7.3E-08 | 1.24E-06 |
| ENSECAG00000009664 | -3.86213 | 9.84E-07 | 1.56E-05 |
| ENSECAG00000012763 | -3.8425 | 9.92E-07 | 1.57E-05 |
| ENSECAG00000015891 | -3.83885 | 2.89E-07 | 4.72E-06 |
| ENSECAG00000002773 | -3.79159 | 2.21E-06 | 3.36E-05 |
| ENSECAG00000002003 | -3.76569 | 8.25E-07 | 1.31E-05 |
| ENSECAG00000023661 | -3.76422 | 6.85E-07 | 1.1E-05 |
| ENSECAG00000007877 | -3.72842 | 1.86E-06 | 2.85E-05 |
| ENSECAG00000020019 | -3.7243 | 1.04E-06 | 1.64E-05 |
| ENSECAG00000023700 | -3.69472 | 2.05E-06 | 3.13E-05 |
| ENSECAG00000007585 | -3.64039 | 3.06E-07 | 4.98E-06 |
| ENSECAG00000024550 | -3.63914 | 1.04E-06 | 1.63E-05 |
| ENSECAG00000016901 | -3.63208 | 2.15E-06 | 3.26E-05 |
| ENSECAG00000006220 | -3.62795 | 1.29E-06 | 2.01E-05 |
| ENSECAG00000006231 | -3.62718 | 1.78E-06 | 2.74E-05 |
| ENSECAG00000011000 | -3.61943 | 1.37E-06 | 2.13E-05 |
| ENSECAG00000018036 | -3.61717 | 1.75E-07 | 2.91E-06 |
| ENSECAG00000014729 | -3.57283 | 3.86E-06 | 5.66E-05 |
| ENSECAG00000009391 | -3.5677 | 2.72E-06 | 4.07E-05 |
| ENSECAG00000003850 | -3.54498 | 4.66E-07 | 7.51E-06 |
| ENSECAG00000003279 | -3.54175 | 2.92E-06 | 4.36E-05 |
| ENSECAG00000013866 | -3.53954 | 1.08E-06 | 1.69E-05 |
| ENSECAG00000013012 | -3.53562 | 2.49E-06 | 3.74E-05 |
| ENSECAG00000009083 | -3.52977 | 1.92E-06 | 2.94E-05 |
| ENSECAG00000000051 | -3.52927 | 1.01E-06 | 1.59E-05 |
| ENSECAG00000004813 | -3.51261 | 4.24E-07 | 6.86E-06 |
| ENSECAG00000017981 | -3.49569 | 1.06E-05 | 0.000147 |
| ENSECAG00000016365 | -3.48482 | 4.14E-06 | 6.05E-05 |
| ENSECAG00000004934 | -3.47638 | 2.73E-06 | 4.07E-05 |
| ENSECAG00000012461 | -3.47408 | 2.14E-06 | 3.25E-05 |
| ENSECAG00000012754 | -3.47086 | 1.7E-06 | 2.63E-05 |
| ENSECAG00000001976 | -3.46822 | 2.49E-06 | 3.74E-05 |
| ENSECAG00000018718 | -3.45907 | 1.67E-06 | 2.58E-05 |
| ENSECAG00000002330 | -3.44971 | 1.76E-06 | 2.72E-05 |
| ENSECAG00000003812 | -3.44757 | 2.72E-06 | 4.07E-05 |
| ENSECAG00000006433 | -3.43483 | 3.37E-07 | 5.48E-06 |
| ENSECAG00000023462 | -3.43044 | 3.38E-06 | 5E-05 |
| ENSECAG00000017060 | -3.41472 | 5.57E-06 | 8E-05 |
| ENSECAG00000010509 | -3.40505 | 7.26E-06 | 0.000103 |
| ENSECAG00000013203 | -3.40457 | 2.28E-06 | 3.45E-05 |
| ENSECAG00000001483 | -3.40119 | 5.39E-06 | 7.76E-05 |
| ENSECAG00000023804 | -3.37867 | 7.15E-06 | 0.000102 |
| ENSECAG00000022452 | -3.37515 | 5.63E-06 | 8.08E-05 |
| ENSECAG00000001424 | -3.36886 | 8.33E-06 | 0.000117 |
| ENSECAG00000004563 | -3.35966 | 3.46E-06 | 5.1E-05 |
| ENSECAG00000012385 | -3.32793 | 6.01E-06 | 8.58E-05 |
| ENSECAG00000016748 | -3.31738 | 1.57E-05 | 0.000212 |
| ENSECAG00000009532 | -3.30994 | 1.04E-05 | 0.000144 |
| ENSECAG00000005746 | -3.30127 | 1.11E-05 | 0.000153 |
| ENSECAG00000003096 | -3.26837 | 0.000103 | 0.001144 |
| ENSECAG00000007398 | -3.25383 | 4.5E-06 | 6.56E-05 |
| ENSECAG00000023475 | -3.25113 | 1.33E-05 | 0.000183 |
| ENSECAG00000008244 | -3.24637 | 1.18E-05 | 0.000162 |
| ENSECAG00000025156 | -3.24449 | 9.21E-06 | 0.000128 |
| ENSECAG00000010405 | -3.2214 | 1.57E-05 | 0.000213 |
| ENSECAG00000008446 | -3.21818 | 1.14E-05 | 0.000157 |
| ENSECAG00000005788 | -3.21085 | 2.23E-06 | 3.38E-05 |
| ENSECAG00000001907 | -3.20916 | 2.84E-06 | 4.24E-05 |
| ENSECAG00000002066 | -3.19712 | 1.84E-05 | 0.000245 |
| ENSECAG00000000563 | -3.19092 | 5.44E-06 | 7.83E-05 |
| ENSECAG00000022779 | -3.18078 | 1.84E-05 | 0.000245 |
| ENSECAG00000004122 | -3.17707 | 2.18E-05 | 0.000286 |
| ENSECAG00000010017 | -3.17642 | 1.51E-05 | 0.000205 |
| ENSECAG00000005375 | -3.17199 | 3E-05 | 0.000379 |
| ENSECAG00000011441 | -3.17084 | 1.49E-05 | 0.000202 |
| ENSECAG00000005621 | -3.16553 | 1.01E-05 | 0.00014 |
| ENSECAG00000010443 | -3.14502 | 2.5E-05 | 0.000323 |
| ENSECAG00000017936 | -3.1443 | 7.28E-06 | 0.000103 |
| ENSECAG00000004977 | -3.13723 | 1.82E-05 | 0.000243 |
| ENSECAG00000005531 | -3.12717 | 1.95E-05 | 0.000258 |
| ENSECAG00000022907 | -3.12218 | 2.82E-05 | 0.000359 |
| ENSECAG00000024145 | -3.11815 | 3.57E-05 | 0.000445 |
| ENSECAG00000017374 | -3.10316 | 3.39E-05 | 0.000424 |
| ENSECAG00000000807 | -3.09401 | 2.14E-05 | 0.000281 |
| ENSECAG00000003411 | -3.08994 | 7.24E-06 | 0.000103 |
| ENSECAG00000021221 | -3.08663 | 3.18E-05 | 0.0004 |
| ENSECAG00000002148 | -3.07577 | 4.9E-05 | 0.000588 |
| ENSECAG00000001631 | -3.07377 | 2.38E-05 | 0.000309 |
| ENSECAG00000001095 | -3.07099 | 2.2E-05 | 0.000287 |
| ENSECAG00000014714 | -3.06501 | 5.7E-06 | 8.15E-05 |
| ENSECAG00000005920 | -3.06317 | 3.33E-05 | 0.000418 |
| ENSECAG00000024254 | -3.06082 | 1.38E-05 | 0.000189 |
| ENSECAG00000005005 | -3.06048 | 2.73E-05 | 0.00035 |
| ENSECAG00000001812 | -3.05847 | 3.11E-05 | 0.000392 |
| ENSECAG00000004659 | -3.05282 | 1.19E-05 | 0.000164 |
| ENSECAG00000022084 | -3.05223 | 2.15E-05 | 0.000282 |
| ENSECAG00000011800 | -3.03652 | 9.24E-06 | 0.000129 |
| ENSECAG00000023006 | -3.03597 | 0.000148 | 0.001577 |
| ENSECAG00000022661 | -3.02718 | 4.71E-05 | 0.000568 |
| ENSECAG00000013060 | -3.02401 | 4.38E-05 | 0.000535 |
| ENSECAG00000015870 | -3.01946 | 4.59E-05 | 0.000555 |
| ENSECAG00000022087 | -3.01828 | 4.09E-05 | 0.000503 |
| ENSECAG00000025025 | -3.01149 | 2.06E-05 | 0.000272 |
| ENSECAG00000013704 | -3.00871 | 5.45E-05 | 0.000648 |
| ENSECAG00000021608 | -3.00633 | 4.79E-05 | 0.000577 |
| ENSECAG00000005036 | -2.99572 | 0.000122 | 0.001332 |
| ENSECAG00000006446 | -2.99281 | 4.87E-05 | 0.000586 |
| ENSECAG00000010843 | -2.98602 | 7.17E-05 | 0.000825 |
| ENSECAG00000019770 | -2.98395 | 5.03E-05 | 0.000603 |
| ENSECAG00000021175 | -2.97827 | 2.98E-05 | 0.000378 |
| ENSECAG00000013001 | -2.96496 | 0.000188 | 0.001934 |
| ENSECAG00000009056 | -2.95976 | 3.34E-05 | 0.000418 |
| ENSECAG00000001922 | -2.95663 | 6.72E-05 | 0.000778 |
| ENSECAG00000023541 | -2.95567 | 0.000107 | 0.001175 |
| ENSECAG00000001520 | -2.95099 | 2.47E-05 | 0.00032 |
| ENSECAG00000018700 | -2.95013 | 6.55E-05 | 0.000761 |
| ENSECAG00000001112 | -2.94976 | 6.08E-05 | 0.000713 |
| ENSECAG00000007209 | -2.94722 | 7.77E-05 | 0.000883 |
| ENSECAG00000022818 | -2.94344 | 4.26E-05 | 0.00052 |
| ENSECAG00000003784 | -2.93207 | 0.000106 | 0.001171 |
| ENSECAG00000018760 | -2.92905 | 8.07E-05 | 0.000913 |
| ENSECAG00000006070 | -2.91771 | 8.84E-05 | 0.000995 |
| ENSECAG00000004943 | -2.91674 | 0.000102 | 0.001128 |
| ENSECAG00000017809 | -2.91418 | 9.49E-05 | 0.00106 |
| ENSECAG00000013438 | -2.90103 | 0.000118 | 0.001287 |
| ENSECAG00000013013 | -2.88839 | 1.48E-05 | 0.000201 |
| ENSECAG00000013559 | -2.88564 | 0.000114 | 0.001257 |
| ENSECAG00000018748 | -2.87496 | 9.83E-05 | 0.001094 |
| ENSECAG00000000978 | -2.8749 | 8.76E-05 | 0.000986 |
| ENSECAG00000018761 | -2.87336 | 6.72E-05 | 0.000778 |
| ENSECAG00000004939 | -2.86174 | 5.52E-05 | 0.000655 |
| ENSECAG00000006367 | -2.86171 | 2.56E-05 | 0.00033 |
| ENSECAG00000020836 | -2.86023 | 1.39E-05 | 0.000189 |
| ENSECAG00000014823 | -2.85237 | 0.000126 | 0.001368 |
| ENSECAG00000008667 | -2.8481 | 3.4E-05 | 0.000425 |
| ENSECAG00000023781 | -2.84656 | 7.16E-05 | 0.000825 |
| ENSECAG00000005907 | -2.84563 | 7.7E-05 | 0.000877 |
| ENSECAG00000019330 | -2.8428 | 0.000183 | 0.001894 |
| ENSECAG00000025112 | -2.83664 | 7.77E-05 | 0.000883 |
| ENSECAG00000013472 | -2.82914 | 0.000149 | 0.001588 |
| ENSECAG00000002776 | -2.82696 | 7.47E-05 | 0.000853 |
| ENSECAG00000011477 | -2.82461 | 9.44E-05 | 0.001057 |
| ENSECAG00000002886 | -2.81076 | 5.61E-05 | 0.000665 |
| ENSECAG00000008263 | -2.80968 | 0.000114 | 0.001255 |
| ENSECAG00000001505 | -2.80951 | 0.000117 | 0.001286 |
| ENSECAG00000023821 | -2.80838 | 9.38E-05 | 0.001051 |
| ENSECAG00000001529 | -2.80094 | 9.91E-05 | 0.001101 |
| ENSECAG00000022053 | -2.79964 | 0.000131 | 0.00142 |
| ENSECAG00000003624 | -2.79547 | 3.56E-05 | 0.000444 |
| ENSECAG00000001717 | -2.79365 | 7.41E-05 | 0.000848 |
| ENSECAG00000006645 | -2.78713 | 0.000277 | 0.002718 |
| ENSECAG00000013902 | -2.78586 | 0.000222 | 0.002236 |
| ENSECAG00000004692 | -2.77861 | 5.93E-05 | 0.0007 |
| ENSECAG00000006264 | -2.77717 | 4.17E-05 | 0.000511 |
| ENSECAG00000017535 | -2.76886 | 0.000211 | 0.002147 |
| ENSECAG00000012949 | -2.76538 | 6.97E-05 | 0.000804 |
| ENSECAG00000023107 | -2.75129 | 0.000195 | 0.001999 |
| ENSECAG00000023283 | -2.74367 | 0.000215 | 0.002178 |
| ENSECAG00000001062 | -2.74339 | 0.000177 | 0.001834 |
| ENSECAG00000023511 | -2.73385 | 0.000324 | 0.003114 |
| ENSECAG00000010797 | -2.73303 | 2.95E-05 | 0.000375 |
| ENSECAG00000004631 | -2.72517 | 0.000211 | 0.002147 |
| ENSECAG00000013293 | -2.71604 | 4.5E-05 | 0.000547 |
| ENSECAG00000001982 | -2.70977 | 0.000758 | 0.006397 |
| ENSECAG00000001936 | -2.70633 | 0.000137 | 0.001476 |
| ENSECAG00000011481 | -2.70484 | 5.47E-05 | 0.000649 |
| ENSECAG00000018822 | -2.70423 | 0.000279 | 0.002733 |
| ENSECAG00000015933 | -2.6902 | 0.000247 | 0.002458 |
| ENSECAG00000022795 | -2.6895 | 5.22E-05 | 0.000622 |
| ENSECAG00000017320 | -2.68372 | 0.000101 | 0.00112 |
| ENSECAG00000005040 | -2.68129 | 0.000296 | 0.002881 |
| ENSECAG00000018877 | -2.67753 | 0.0003 | 0.002912 |
| ENSECAG00000022711 | -2.67547 | 0.000134 | 0.001443 |
| ENSECAG00000010180 | -2.67085 | 0.00017 | 0.001773 |
| ENSECAG00000024466 | -2.66091 | 0.000161 | 0.001708 |
| ENSECAG00000002175 | -2.65988 | 0.000168 | 0.001766 |
| ENSECAG00000002349 | -2.65988 | 0.000168 | 0.001766 |
| ENSECAG00000004787 | -2.65988 | 0.000168 | 0.001766 |
| ENSECAG00000011999 | -2.65988 | 0.000168 | 0.001766 |
| ENSECAG00000017032 | -2.65905 | 0.000167 | 0.001758 |
| ENSECAG00000005350 | -2.65813 | 0.00017 | 0.001773 |
| ENSECAG00000002269 | -2.65706 | 5.3E-05 | 0.000631 |
| ENSECAG00000001381 | -2.65679 | 0.000716 | 0.006109 |
| ENSECAG00000004307 | -2.65514 | 0.000269 | 0.002643 |
| ENSECAG00000001635 | -2.65496 | 0.000337 | 0.003222 |
| ENSECAG00000003792 | -2.65025 | 0.000177 | 0.001836 |
| ENSECAG00000020520 | -2.64502 | 0.000167 | 0.001761 |
| ENSECAG00000012015 | -2.62917 | 0.000244 | 0.002434 |
| ENSECAG00000022792 | -2.61916 | 9.49E-05 | 0.00106 |
| ENSECAG00000003708 | -2.61694 | 9.93E-05 | 0.001102 |
| ENSECAG00000005818 | -2.61617 | 0.000322 | 0.003103 |
| ENSECAG00000001709 | -2.61095 | 0.000537 | 0.004784 |
| ENSECAG00000019759 | -2.60951 | 0.000147 | 0.001569 |
| ENSECAG00000022903 | -2.59523 | 0.000435 | 0.004005 |
| ENSECAG00000006809 | -2.59314 | 0.000333 | 0.003189 |
| ENSECAG00000005057 | -2.5931 | 0.00035 | 0.003329 |
| ENSECAG00000005276 | -2.59059 | 0.000201 | 0.00206 |
| ENSECAG00000022746 | -2.58081 | 0.000149 | 0.001584 |
| ENSECAG00000024725 | -2.57815 | 0.000241 | 0.002407 |
| ENSECAG00000004550 | -2.57156 | 0.000243 | 0.00243 |
| ENSECAG00000020371 | -2.56771 | 0.000142 | 0.00152 |
| ENSECAG00000004402 | -2.5646 | 0.000357 | 0.003373 |
| ENSECAG00000012971 | -2.56364 | 0.000478 | 0.004331 |
| ENSECAG00000022535 | -2.55912 | 0.000169 | 0.001766 |
| ENSECAG00000021739 | -2.55849 | 0.000441 | 0.004055 |
| ENSECAG00000000394 | -2.55717 | 0.000257 | 0.002542 |
| ENSECAG00000017069 | -2.55671 | 0.000266 | 0.002613 |
| ENSECAG00000008589 | -2.55622 | 0.000849 | 0.007016 |
| ENSECAG00000007178 | -2.55547 | 0.000563 | 0.004994 |
| ENSECAG00000010040 | -2.55425 | 0.000272 | 0.002666 |
| ENSECAG00000006412 | -2.54901 | 0.000354 | 0.00336 |
| ENSECAG00000006639 | -2.54763 | 0.000519 | 0.004649 |
| ENSECAG00000003936 | -2.54677 | 0.00017 | 0.001773 |
| ENSECAG00000006154 | -2.54651 | 0.000302 | 0.002925 |
| ENSECAG00000010217 | -2.54034 | 0.000358 | 0.003374 |
| ENSECAG00000005900 | -2.53732 | 0.000649 | 0.005638 |
| ENSECAG00000006514 | -2.53655 | 0.000606 | 0.005328 |
| ENSECAG00000004466 | -2.53625 | 0.000458 | 0.004182 |
| ENSECAG00000003696 | -2.53157 | 0.000638 | 0.005566 |
| ENSECAG00000024366 | -2.5287 | 0.000645 | 0.005615 |
| ENSECAG00000005777 | -2.52645 | 0.000644 | 0.005615 |
| ENSECAG00000021536 | -2.52191 | 0.000325 | 0.003115 |
| ENSECAG00000011796 | -2.52178 | 0.000342 | 0.003269 |
| ENSECAG00000006956 | -2.51657 | 0.000248 | 0.002473 |
| ENSECAG00000004237 | -2.51501 | 0.000348 | 0.003308 |
| ENSECAG00000014419 | -2.51087 | 0.000344 | 0.003285 |
| ENSECAG00000007110 | -2.50801 | 0.0003 | 0.002912 |
| ENSECAG00000004410 | -2.50314 | 0.000731 | 0.006207 |
| ENSECAG00000001536 | -2.50287 | 0.000323 | 0.003107 |
| ENSECAG00000004285 | -2.50072 | 0.000123 | 0.001338 |
| ENSECAG00000020177 | -2.49945 | 0.000347 | 0.003302 |
| ENSECAG00000004840 | -2.49785 | 0.000773 | 0.006499 |
| ENSECAG00000008008 | -2.49414 | 0.000109 | 0.001205 |
| ENSECAG00000001933 | -2.48692 | 0.00078 | 0.006546 |
| ENSECAG00000003860 | -2.48692 | 0.00078 | 0.006546 |
| ENSECAG00000002844 | -2.4865 | 0.00046 | 0.004196 |
| ENSECAG00000017029 | -2.48596 | 0.000155 | 0.001645 |
| ENSECAG00000021575 | -2.48571 | 0.000417 | 0.003858 |
| ENSECAG00000002637 | -2.48539 | 0.000357 | 0.003373 |
| ENSECAG00000006762 | -2.48404 | 0.000368 | 0.003462 |
| ENSECAG00000015111 | -2.48144 | 0.00036 | 0.003387 |
| ENSECAG00000003333 | -2.48046 | 0.000396 | 0.003685 |
| ENSECAG00000021197 | -2.47963 | 0.000807 | 0.006716 |
| ENSECAG00000010994 | -2.47947 | 0.000439 | 0.004033 |
| ENSECAG00000013204 | -2.47902 | 0.00045 | 0.004124 |
| ENSECAG00000003969 | -2.47689 | 0.00072 | 0.006129 |
| ENSECAG00000005484 | -2.47546 | 0.000419 | 0.003871 |
| ENSECAG00000008031 | -2.47522 | 0.000117 | 0.001286 |
| ENSECAG00000004320 | -2.47494 | 0.000356 | 0.003373 |
| ENSECAG00000007544 | -2.47133 | 3.86E-05 | 0.000478 |
| ENSECAG00000002881 | -2.47099 | 0.000758 | 0.006397 |
| ENSECAG00000014277 | -2.47082 | 0.000402 | 0.003734 |
| ENSECAG00000024572 | -2.46988 | 0.000527 | 0.004713 |
| ENSECAG00000003679 | -2.46968 | 0.000808 | 0.006722 |
| ENSECAG00000004656 | -2.46653 | 0.000374 | 0.003509 |
| ENSECAG00000001991 | -2.46619 | 0.000871 | 0.007158 |
| ENSECAG00000004511 | -2.46602 | 0.00067 | 0.005774 |
| ENSECAG00000003327 | -2.46601 | 0.000807 | 0.006716 |
| ENSECAG00000022444 | -2.45788 | 1.61E-05 | 0.000218 |
| ENSECAG00000002214 | -2.45697 | 0.000678 | 0.005831 |
| ENSECAG00000002351 | -2.45425 | 0.001006 | 0.00814 |
| ENSECAG00000004895 | -2.4483 | 0.000544 | 0.004838 |
| ENSECAG00000019464 | -2.44666 | 0.000817 | 0.006784 |
| ENSECAG00000019924 | -2.44357 | 0.000474 | 0.004306 |
| ENSECAG00000002774 | -2.44169 | 0.000645 | 0.005615 |
| ENSECAG00000023435 | -2.43497 | 0.000212 | 0.002154 |
| ENSECAG00000000211 | -2.42498 | 0.000477 | 0.00433 |
| ENSECAG00000004451 | -2.42265 | 0.000563 | 0.004994 |
| ENSECAG00000003627 | -2.42197 | 0.001057 | 0.008502 |
| ENSECAG00000005274 | -2.4201 | 0.000507 | 0.004565 |
| ENSECAG00000004654 | -2.41882 | 0.000449 | 0.004124 |
| ENSECAG00000021183 | -2.41828 | 0.000654 | 0.005669 |
| ENSECAG00000022226 | -2.41803 | 0.000462 | 0.004206 |
| ENSECAG00000002248 | -2.41798 | 0.000645 | 0.005615 |
| ENSECAG00000022170 | -2.4154 | 0.000565 | 0.005006 |
| ENSECAG00000012088 | -2.40959 | 0.000901 | 0.007387 |
| ENSECAG00000016760 | -2.40893 | 0.000371 | 0.003482 |
| ENSECAG00000020837 | -2.40608 | 0.000844 | 0.006986 |
| ENSECAG00000003063 | -2.40508 | 0.000668 | 0.005764 |
| ENSECAG00000006598 | -2.4037 | 0.000604 | 0.005313 |
| ENSECAG00000002202 | -2.40213 | 0.000493 | 0.004461 |
| ENSECAG00000011740 | -2.40045 | 0.000585 | 0.00517 |
| ENSECAG00000001917 | -2.39891 | 0.001134 | 0.00901 |
| ENSECAG00000001590 | -2.39816 | 0.001014 | 0.008186 |
| ENSECAG00000021293 | -2.39643 | 0.00035 | 0.003329 |
| ENSECAG00000003212 | -2.3929 | 0.001261 | 0.009888 |
| ENSECAG00000005245 | -2.39202 | 0.000509 | 0.004573 |
| ENSECAG00000004537 | -2.38394 | 0.001125 | 0.008952 |
| ENSECAG00000001132 | -2.37982 | 0.001122 | 0.008932 |
| ENSECAG00000005931 | -2.37883 | 0.000654 | 0.005669 |
| ENSECAG00000011937 | -2.37479 | 0.000345 | 0.003285 |
| ENSECAG00000002071 | -2.36879 | 0.000741 | 0.006273 |
| ENSECAG00000018494 | -2.36552 | 0.000787 | 0.006585 |
| ENSECAG00000001876 | -2.36246 | 0.000651 | 0.005649 |
| ENSECAG00000005206 | -2.3571 | 0.00067 | 0.005774 |
| ENSECAG00000021779 | -2.35535 | 0.000735 | 0.006231 |
| ENSECAG00000006900 | -2.35112 | 0.000625 | 0.005475 |
| ENSECAG00000002872 | -2.35089 | 0.000758 | 0.006397 |
| ENSECAG00000010952 | -2.35007 | 0.000616 | 0.005401 |
| ENSECAG00000003196 | -2.34531 | 0.001009 | 0.008157 |
| ENSECAG00000002120 | -2.34048 | 0.000869 | 0.007147 |
| ENSECAG00000005488 | -2.34026 | 0.00081 | 0.006731 |
| ENSECAG00000002279 | -2.34017 | 0.00033 | 0.003168 |
| ENSECAG00000021860 | -2.33684 | 0.000804 | 0.006702 |
| ENSECAG00000018717 | -2.33643 | 2.63E-05 | 0.000338 |
| ENSECAG00000002646 | -2.32957 | 0.000404 | 0.003749 |
| ENSECAG00000019595 | -2.32944 | 0.000871 | 0.00716 |
| ENSECAG00000007430 | -2.32009 | 0.000771 | 0.006495 |
| ENSECAG00000015085 | -2.31637 | 0.000707 | 0.006035 |
| ENSECAG00000002034 | -2.3076 | 0.000855 | 0.007051 |
| ENSECAG00000006531 | -2.30543 | 0.000661 | 0.00572 |
| ENSECAG00000007893 | -2.30003 | 0.000908 | 0.007441 |
| ENSECAG00000005859 | -2.29615 | 0.000785 | 0.006571 |
| ENSECAG00000005441 | -2.29357 | 0.000828 | 0.006863 |
| ENSECAG00000004896 | -2.29147 | 0.000833 | 0.006902 |
| ENSECAG00000014634 | -2.28973 | 0.001166 | 0.009235 |
| ENSECAG00000024037 | -2.28658 | 0.000863 | 0.007108 |
| ENSECAG00000009592 | -2.27873 | 0.000425 | 0.003913 |
| ENSECAG00000014748 | -2.27738 | 0.001082 | 0.008689 |
| ENSECAG00000001539 | -2.27657 | 0.000944 | 0.007684 |
| ENSECAG00000007485 | -2.27387 | 0.000324 | 0.003115 |
| ENSECAG00000017236 | -2.27109 | 0.001124 | 0.00895 |
| ENSECAG00000001847 | -2.26607 | 0.001182 | 0.009351 |
| ENSECAG00000025077 | -2.26589 | 0.001073 | 0.008617 |
| ENSECAG00000003932 | -2.26506 | 0.001197 | 0.009449 |
| ENSECAG00000019548 | -2.26378 | 0.001164 | 0.009224 |
| ENSECAG00000017652 | -2.26235 | 0.001208 | 0.00953 |
| ENSECAG00000005189 | -2.24164 | 0.00096 | 0.007795 |
| ENSECAG00000022904 | -2.23064 | 0.001015 | 0.008189 |
| ENSECAG00000002940 | -2.2279 | 0.000627 | 0.005488 |
| ENSECAG00000024735 | -2.22247 | 0.001262 | 0.00989 |
| ENSECAG00000014060 | -2.22203 | 0.00118 | 0.009342 |
| ENSECAG00000001766 | -2.21905 | 0.000679 | 0.005837 |
| ENSECAG00000024524 | -2.21265 | 0.0011 | 0.008791 |
| ENSECAG00000006232 | -2.21242 | 0.001183 | 0.009351 |
| ENSECAG00000001874 | -2.19284 | 0.001041 | 0.008382 |
| ENSECAG00000006394 | -2.19036 | 0.001223 | 0.009627 |
| ENSECAG00000022148 | -2.18813 | 0.000948 | 0.007712 |
| ENSECAG00000008890 | -2.15207 | 0.000775 | 0.006509 |
| ENSECAG00000005965 | -2.15052 | 0.000707 | 0.006035 |
| ENSECAG00000000448 | -2.13762 | 0.001145 | 0.00909 |
| ENSECAG00000004754 | -2.1293 | 0.000746 | 0.006312 |
| ENSECAG00000004394 | -2.09808 | 0.001103 | 0.008807 |
| ENSECAG00000021873 | -2.06624 | 0.000796 | 0.006647 |
| ENSECAG00000021894 | -2.00431 | 0.001217 | 0.009583 |
| ENSECAG00000001363 | -1.99071 | 0.00059 | 0.00521 |
| ENSECAG00000000461 | -1.92196 | 0.000168 | 0.001766 |
| ENSECAG00000001992 | 1.921061 | 0.001191 | 0.00941 |
| ENSECAG00000002714 | 1.928338 | 0.001031 | 0.008313 |
| ENSECAG00000024499 | 1.941151 | 0.001093 | 0.008744 |
| ENSECAG00000010583 | 1.976727 | 0.001276 | 0.009969 |
| ENSECAG00000020393 | 2.018123 | 0.001069 | 0.008592 |
| ENSECAG00000020949 | 2.020096 | 0.000988 | 0.008 |
| ENSECAG00000009315 | 2.024996 | 0.001274 | 0.00996 |
| ENSECAG00000014276 | 2.044958 | 0.000729 | 0.006193 |
| ENSECAG00000015319 | 2.048768 | 0.001258 | 0.009873 |
| ENSECAG00000016313 | 2.054008 | 0.000913 | 0.007463 |
| ENSECAG00000021334 | 2.062467 | 0.000853 | 0.007044 |
| ENSECAG00000004306 | 2.069337 | 0.000452 | 0.004139 |
| ENSECAG00000024301 | 2.071411 | 0.000664 | 0.005731 |
| ENSECAG00000005886 | 2.07584 | 0.000912 | 0.007453 |
| ENSECAG00000000018 | 2.076078 | 0.000845 | 0.006986 |
| ENSECAG00000017817 | 2.076083 | 0.000928 | 0.00757 |
| ENSECAG00000008348 | 2.076131 | 0.00096 | 0.007795 |
| ENSECAG00000021003 | 2.079747 | 0.000646 | 0.005619 |
| ENSECAG00000023647 | 2.084544 | 0.000681 | 0.00585 |
| ENSECAG00000002088 | 2.084721 | 0.000533 | 0.004765 |
| ENSECAG00000005558 | 2.086668 | 0.001132 | 0.008998 |
| ENSECAG00000009788 | 2.0878 | 0.0008 | 0.006682 |
| ENSECAG00000018535 | 2.09055 | 0.000412 | 0.003826 |
| ENSECAG00000023324 | 2.091682 | 0.000835 | 0.006916 |
| ENSECAG00000008230 | 2.097046 | 0.001273 | 0.00996 |
| ENSECAG00000010436 | 2.103243 | 0.000909 | 0.007441 |
| ENSECAG00000001130 | 2.103588 | 0.000663 | 0.005726 |
| ENSECAG00000008521 | 2.106601 | 0.000419 | 0.003869 |
| ENSECAG00000024136 | 2.107115 | 0.000509 | 0.004577 |
| ENSECAG00000019201 | 2.113295 | 0.000728 | 0.006188 |
| ENSECAG00000018790 | 2.119307 | 0.000879 | 0.007221 |
| ENSECAG00000009216 | 2.12711 | 0.000478 | 0.004331 |
| ENSECAG00000000132 | 2.135286 | 0.000684 | 0.005868 |
| ENSECAG00000014430 | 2.135784 | 0.001273 | 0.00996 |
| ENSECAG00000020833 | 2.136739 | 0.000513 | 0.004599 |
| ENSECAG00000016746 | 2.140796 | 0.000818 | 0.006792 |
| ENSECAG00000014368 | 2.144021 | 0.000647 | 0.005621 |
| ENSECAG00000011996 | 2.14595 | 0.001013 | 0.008181 |
| ENSECAG00000013760 | 2.149864 | 0.000592 | 0.005221 |
| ENSECAG00000015855 | 2.150507 | 0.000806 | 0.006715 |
| ENSECAG00000018988 | 2.161543 | 0.000534 | 0.00477 |
| ENSECAG00000015448 | 2.167334 | 0.001156 | 0.009172 |
| ENSECAG00000015585 | 2.172863 | 0.000453 | 0.004149 |
| ENSECAG00000000227 | 2.173958 | 0.000784 | 0.006571 |
| ENSECAG00000003770 | 2.175651 | 0.00068 | 0.005845 |
| ENSECAG00000014603 | 2.178825 | 0.001052 | 0.008466 |
| ENSECAG00000005552 | 2.181875 | 0.001158 | 0.009182 |
| ENSECAG00000021273 | 2.186588 | 0.000508 | 0.00457 |
| ENSECAG00000017416 | 2.200365 | 0.000597 | 0.005258 |
| ENSECAG00000020164 | 2.204869 | 0.000512 | 0.004598 |
| ENSECAG00000023479 | 2.208551 | 0.000554 | 0.004915 |
| ENSECAG00000016968 | 2.213091 | 0.00027 | 0.002654 |
| ENSECAG00000022081 | 2.213456 | 0.000383 | 0.003586 |
| ENSECAG00000024743 | 2.216988 | 0.000535 | 0.004777 |
| ENSECAG00000022551 | 2.231193 | 0.000396 | 0.003685 |
| ENSECAG00000009985 | 2.232977 | 0.000518 | 0.004645 |
| ENSECAG00000013170 | 2.235489 | 0.001093 | 0.008744 |
| ENSECAG00000004305 | 2.246481 | 0.001093 | 0.008744 |
| ENSECAG00000014055 | 2.247428 | 0.000863 | 0.007107 |
| ENSECAG00000024356 | 2.251318 | 0.000881 | 0.007231 |
| ENSECAG00000011316 | 2.252747 | 0.000553 | 0.004915 |
| ENSECAG00000014185 | 2.257729 | 0.000379 | 0.00355 |
| ENSECAG00000010932 | 2.264318 | 0.000542 | 0.004822 |
| ENSECAG00000007052 | 2.264544 | 0.000297 | 0.002887 |
| ENSECAG00000001316 | 2.269301 | 0.000265 | 0.002606 |
| ENSECAG00000014459 | 2.271856 | 0.000579 | 0.00512 |
| ENSECAG00000011276 | 2.276277 | 0.000327 | 0.003139 |
| ENSECAG00000007385 | 2.281877 | 0.000345 | 0.003285 |
| ENSECAG00000022532 | 2.314396 | 0.000228 | 0.002287 |
| ENSECAG00000003211 | 2.31798 | 0.000463 | 0.004214 |
| ENSECAG00000004061 | 2.319174 | 0.000677 | 0.00583 |
| ENSECAG00000015051 | 2.32664 | 0.000718 | 0.006117 |
| ENSECAG00000023940 | 2.348415 | 0.000258 | 0.002547 |
| ENSECAG00000000983 | 2.349484 | 0.000245 | 0.002439 |
| ENSECAG00000006525 | 2.354763 | 0.000295 | 0.002877 |
| ENSECAG00000003946 | 2.364861 | 0.000681 | 0.00585 |
| ENSECAG00000001999 | 2.368254 | 0.00051 | 0.004582 |
| ENSECAG00000016814 | 2.369879 | 0.000785 | 0.006571 |
| ENSECAG00000011407 | 2.370547 | 0.000175 | 0.001816 |
| ENSECAG00000012770 | 2.371572 | 0.000217 | 0.002191 |
| ENSECAG00000001996 | 2.381 | 0.000249 | 0.002479 |
| ENSECAG00000018283 | 2.391277 | 0.000226 | 0.002276 |
| ENSECAG00000020891 | 2.399978 | 0.000184 | 0.001901 |
| ENSECAG00000003414 | 2.413813 | 0.000129 | 0.001404 |
| ENSECAG00000020320 | 2.418881 | 0.000761 | 0.006416 |
| ENSECAG00000010668 | 2.421681 | 0.000116 | 0.001276 |
| ENSECAG00000005128 | 2.431729 | 0.001104 | 0.008813 |
| ENSECAG00000011084 | 2.432874 | 0.000164 | 0.001729 |
| ENSECAG00000000112 | 2.433706 | 0.001039 | 0.008375 |
| ENSECAG00000009871 | 2.435858 | 0.000177 | 0.001836 |
| ENSECAG00000016509 | 2.437788 | 0.000304 | 0.002948 |
| ENSECAG00000005891 | 2.452909 | 0.00015 | 0.001596 |
| ENSECAG00000005827 | 2.462497 | 0.000136 | 0.001464 |
| ENSECAG00000020269 | 2.465423 | 0.000576 | 0.005099 |
| ENSECAG00000011712 | 2.468142 | 0.000216 | 0.002183 |
| ENSECAG00000004652 | 2.472083 | 0.000312 | 0.003016 |
| ENSECAG00000022321 | 2.480803 | 0.000134 | 0.001448 |
| ENSECAG00000005126 | 2.483071 | 0.000211 | 0.00215 |
| ENSECAG00000004688 | 2.487532 | 0.00029 | 0.002827 |
| ENSECAG00000015799 | 2.487605 | 0.000606 | 0.005328 |
| ENSECAG00000009644 | 2.488849 | 0.000132 | 0.001427 |
| ENSECAG00000018646 | 2.49483 | 0.000239 | 0.002395 |
| ENSECAG00000024341 | 2.500951 | 0.000312 | 0.003015 |
| ENSECAG00000021885 | 2.505853 | 0.000464 | 0.004219 |
| ENSECAG00000022797 | 2.50927 | 0.000163 | 0.001727 |
| ENSECAG00000013881 | 2.514713 | 0.000179 | 0.001853 |
| ENSECAG00000022834 | 2.516404 | 7.98E-05 | 0.000904 |
| ENSECAG00000020847 | 2.519297 | 0.000189 | 0.001948 |
| ENSECAG00000017544 | 2.519333 | 0.000415 | 0.003845 |
| ENSECAG00000005461 | 2.522403 | 0.000256 | 0.002538 |
| ENSECAG00000008571 | 2.527403 | 0.000146 | 0.001562 |
| ENSECAG00000003146 | 2.547919 | 0.000104 | 0.001155 |
| ENSECAG00000007229 | 2.554922 | 0.000106 | 0.001172 |
| ENSECAG00000014242 | 2.565414 | 0.000206 | 0.002105 |
| ENSECAG00000019309 | 2.574576 | 4.5E-05 | 0.000547 |
| ENSECAG00000015912 | 2.578182 | 4.87E-05 | 0.000586 |
| ENSECAG00000015971 | 2.583182 | 0.000438 | 0.004031 |
| ENSECAG00000015840 | 2.6013 | 0.000506 | 0.004562 |
| ENSECAG00000023338 | 2.604958 | 0.000114 | 0.001258 |
| ENSECAG00000022184 | 2.61313 | 0.001275 | 0.00996 |
| ENSECAG00000007661 | 2.619589 | 0.0002 | 0.002053 |
| ENSECAG00000008729 | 2.626398 | 9.7E-05 | 0.001081 |
| ENSECAG00000009320 | 2.627558 | 0.0002 | 0.002053 |
| ENSECAG00000020183 | 2.639431 | 0.000854 | 0.007044 |
| ENSECAG00000009316 | 2.64422 | 0.000207 | 0.002111 |
| ENSECAG00000011914 | 2.64497 | 0.00046 | 0.004193 |
| ENSECAG00000023259 | 2.647585 | 5.97E-05 | 0.000704 |
| ENSECAG00000007006 | 2.650526 | 5.16E-05 | 0.000617 |
| ENSECAG00000026992 | 2.651388 | 6.22E-05 | 0.000729 |
| ENSECAG00000019441 | 2.651458 | 0.000212 | 0.002154 |
| ENSECAG00000003530 | 2.655634 | 0.00045 | 0.004124 |
| ENSECAG00000018222 | 2.656439 | 4.45E-05 | 0.000543 |
| ENSECAG00000008635 | 2.661699 | 4E-05 | 0.000492 |
| ENSECAG00000014955 | 2.673829 | 6.02E-05 | 0.000708 |
| ENSECAG00000023789 | 2.692011 | 3.67E-05 | 0.000456 |
| ENSECAG00000010596 | 2.709966 | 8.73E-05 | 0.000983 |
| ENSECAG00000016298 | 2.711381 | 0.000173 | 0.001797 |
| ENSECAG00000006302 | 2.730814 | 3.77E-05 | 0.000467 |
| ENSECAG00000026951 | 2.743243 | 3.39E-05 | 0.000424 |
| ENSECAG00000012086 | 2.743281 | 7.61E-05 | 0.000868 |
| ENSECAG00000009279 | 2.764254 | 3.4E-05 | 0.000425 |
| ENSECAG00000010447 | 2.769281 | 2.57E-05 | 0.000331 |
| ENSECAG00000015276 | 2.769519 | 8.61E-05 | 0.000971 |
| ENSECAG00000012792 | 2.785922 | 0.000106 | 0.001172 |
| ENSECAG00000013493 | 2.791259 | 2.48E-05 | 0.000321 |
| ENSECAG00000017565 | 2.821102 | 2.97E-05 | 0.000377 |
| ENSECAG00000013320 | 2.821399 | 1.82E-05 | 0.000243 |
| ENSECAG00000014810 | 2.84142 | 3.94E-05 | 0.000487 |
| ENSECAG00000023840 | 2.846927 | 0.000176 | 0.001828 |
| ENSECAG00000016338 | 2.862969 | 3.29E-05 | 0.000413 |
| ENSECAG00000015900 | 2.863153 | 0.000127 | 0.00138 |
| ENSECAG00000000844 | 2.866911 | 0.000911 | 0.007453 |
| ENSECAG00000022147 | 2.870702 | 1.42E-05 | 0.000194 |
| ENSECAG00000016764 | 2.871783 | 2.96E-05 | 0.000375 |
| ENSECAG00000015282 | 2.886113 | 4.75E-05 | 0.000573 |
| ENSECAG00000012594 | 2.89754 | 2.82E-05 | 0.000359 |
| ENSECAG00000006546 | 2.903442 | 1.09E-05 | 0.00015 |
| ENSECAG00000018084 | 2.904929 | 3.64E-05 | 0.000453 |
| ENSECAG00000012866 | 2.909809 | 0.000177 | 0.001836 |
| ENSECAG00000009833 | 2.928229 | 1.99E-05 | 0.000263 |
| ENSECAG00000009870 | 2.941908 | 8.3E-06 | 0.000117 |
| ENSECAG00000006276 | 2.948172 | 3.99E-05 | 0.000492 |
| ENSECAG00000013194 | 2.964515 | 0.000254 | 0.002525 |
| ENSECAG00000020314 | 2.97125 | 1.37E-05 | 0.000187 |
| ENSECAG00000023717 | 3.00214 | 8.31E-06 | 0.000117 |
| ENSECAG00000020498 | 3.038198 | 6.32E-06 | 9E-05 |
| ENSECAG00000018362 | 3.041635 | 6.54E-06 | 9.3E-05 |
| ENSECAG00000013591 | 3.081491 | 2.1E-05 | 0.000276 |
| ENSECAG00000003160 | 3.083102 | 0.000635 | 0.005551 |
| ENSECAG00000015945 | 3.097777 | 8.92E-06 | 0.000125 |
| ENSECAG00000005415 | 3.147662 | 1.93E-05 | 0.000256 |
| ENSECAG00000002630 | 3.152636 | 7.35E-05 | 0.000842 |
| ENSECAG00000013381 | 3.154865 | 9.85E-06 | 0.000137 |
| ENSECAG00000007374 | 3.164748 | 3.28E-05 | 0.000413 |
| ENSECAG00000017200 | 3.186252 | 1.82E-05 | 0.000243 |
| ENSECAG00000000244 | 3.18674 | 3.53E-06 | 5.19E-05 |
| ENSECAG00000004388 | 3.203798 | 6.3E-05 | 0.000737 |
| ENSECAG00000008468 | 3.210022 | 4.71E-06 | 6.84E-05 |
| ENSECAG00000016039 | 3.213581 | 0.000388 | 0.003625 |
| ENSECAG00000009490 | 3.223516 | 1.8E-06 | 2.77E-05 |
| ENSECAG00000004398 | 3.223876 | 2.03E-05 | 0.000268 |
| ENSECAG00000015561 | 3.236128 | 2.05E-06 | 3.13E-05 |
| ENSECAG00000024759 | 3.240732 | 0.000129 | 0.001399 |
| ENSECAG00000019985 | 3.250308 | 6.43E-06 | 9.16E-05 |
| ENSECAG00000003249 | 3.254749 | 7.49E-06 | 0.000106 |
| ENSECAG00000022885 | 3.256896 | 2.11E-05 | 0.000278 |
| ENSECAG00000010687 | 3.257048 | 2.79E-05 | 0.000356 |
| ENSECAG00000005256 | 3.271935 | 1.11E-05 | 0.000153 |
| ENSECAG00000020772 | 3.301727 | 4.56E-06 | 6.63E-05 |
| ENSECAG00000004302 | 3.306926 | 5.66E-06 | 8.11E-05 |
| ENSECAG00000001120 | 3.317366 | 2.76E-05 | 0.000353 |
| ENSECAG00000008143 | 3.319066 | 1.78E-06 | 2.74E-05 |
| ENSECAG00000016234 | 3.347437 | 3.91E-06 | 5.72E-05 |
| ENSECAG00000021522 | 3.353034 | 0.000354 | 0.00336 |
| ENSECAG00000020093 | 3.353725 | 8.86E-05 | 0.000995 |
| ENSECAG00000007601 | 3.368882 | 3.43E-06 | 5.06E-05 |
| ENSECAG00000007303 | 3.388938 | 0.000311 | 0.00301 |
| ENSECAG00000008303 | 3.405699 | 7.81E-07 | 1.24E-05 |
| ENSECAG00000006079 | 3.429564 | 3.52E-07 | 5.71E-06 |
| ENSECAG00000010618 | 3.451516 | 4.52E-07 | 7.29E-06 |
| ENSECAG00000012473 | 3.500856 | 3.15E-06 | 4.69E-05 |
| ENSECAG00000018711 | 3.505126 | 7.63E-06 | 0.000108 |
| ENSECAG00000009840 | 3.540063 | 1.67E-06 | 2.59E-05 |
| ENSECAG00000021514 | 3.598852 | 1.05E-06 | 1.65E-05 |
| ENSECAG00000009782 | 3.614859 | 3.77E-05 | 0.000467 |
| ENSECAG00000008740 | 3.655378 | 6.43E-05 | 0.00075 |
| ENSECAG00000011367 | 3.659833 | 6.19E-07 | 9.93E-06 |
| ENSECAG00000010104 | 3.719766 | 3.86E-06 | 5.66E-05 |
| ENSECAG00000016932 | 3.773497 | 6.28E-07 | 1.01E-05 |
| ENSECAG00000018817 | 3.817544 | 9.89E-08 | 1.67E-06 |
| ENSECAG00000015776 | 3.821057 | 9.41E-07 | 1.49E-05 |
| ENSECAG00000017459 | 3.852195 | 1.22E-07 | 2.04E-06 |
| ENSECAG00000009629 | 3.905059 | 3.89E-08 | 6.75E-07 |
| ENSECAG00000017025 | 3.961564 | 3.83E-05 | 0.000474 |
| ENSECAG00000014189 | 3.964914 | 9.58E-08 | 1.62E-06 |
| ENSECAG00000003179 | 3.975922 | 2.92E-07 | 4.76E-06 |
| ENSECAG00000001602 | 3.997431 | 6.35E-08 | 1.08E-06 |
| ENSECAG00000014919 | 4.135561 | 4.01E-08 | 6.92E-07 |
| ENSECAG00000023307 | 4.135561 | 4.01E-08 | 6.92E-07 |
| ENSECAG00000018945 | 4.193153 | 1.65E-05 | 0.000222 |
| ENSECAG00000016400 | 4.213932 | 3.8E-08 | 6.6E-07 |
| ENSECAG00000020604 | 4.42198 | 9.41E-08 | 1.59E-06 |
| ENSECAG00000021363 | 4.499673 | 2.56E-09 | 4.89E-08 |
| ENSECAG00000002265 | 4.512732 | 1.02E-07 | 1.72E-06 |
| ENSECAG00000019971 | 4.569559 | 6.02E-08 | 1.03E-06 |
| ENSECAG00000000886 | 4.659697 | 2.78E-09 | 5.27E-08 |
| ENSECAG00000000911 | 4.659697 | 2.78E-09 | 5.27E-08 |
| ENSECAG00000008339 | 4.659697 | 2.78E-09 | 5.27E-08 |
| ENSECAG00000009289 | 5.023452 | 9.92E-10 | 1.98E-08 |
| ENSECAG00000013808 | 5.25877 | 3.27E-10 | 6.8E-09 |
| ENSECAG00000001332 | 5.277661 | 1.48E-09 | 2.87E-08 |
| ENSECAG00000011469 | 5.46266 | 2.01E-09 | 3.88E-08 |
| ENSECAG00000021620 | 5.919877 | 1.24E-12 | 3.24E-11 |
| ENSECAG00000020202 | 6.084745 | 2.39E-11 | 5.48E-10 |
| ENSECAG00000014965 | 8.386707 | 1E-08 | 1.8E-07 |
| ENSECAG00000023823 | 8.402779 | 1.88E-08 | 3.31E-07 |
| ENSECAG00000023153 | 8.434395 | 1.69E-08 | 2.98E-07 |
| ENSECAG00000020750 | 8.449947 | 2.9E-09 | 5.47E-08 |
| ENSECAG00000017767 | 8.449947 | 7.42E-09 | 1.34E-07 |
| ENSECAG00000015368 | 8.465334 | 1E-08 | 1.8E-07 |
| ENSECAG00000005394 | 8.480557 | 5.05E-09 | 9.27E-08 |
| ENSECAG00000015503 | 8.58285 | 3.81E-09 | 7.09E-08 |
| ENSECAG00000005529 | 8.596889 | 3.48E-09 | 6.51E-08 |
| ENSECAG00000018321 | 8.610792 | 4.18E-09 | 7.73E-08 |
| ENSECAG00000026996 | 8.656818 | 1.14E-09 | 2.24E-08 |
| ENSECAG00000016631 | 8.665103 | 1.34E-09 | 2.62E-08 |
| ENSECAG00000000067 | 8.678367 | 2.04E-09 | 3.94E-08 |
| ENSECAG00000014788 | 8.678367 | 4.18E-09 | 7.73E-08 |
| ENSECAG00000019118 | 8.678367 | 4.18E-09 | 7.73E-08 |
| ENSECAG00000021336 | 8.697517 | 1.05E-09 | 2.07E-08 |
| ENSECAG00000008071 | 8.704535 | 2.43E-09 | 4.66E-08 |
| ENSECAG00000000916 | 8.717444 | 2.23E-09 | 4.29E-08 |
| ENSECAG00000007212 | 8.742918 | 2.9E-09 | 5.47E-08 |
| ENSECAG00000016063 | 8.755489 | 1.05E-09 | 2.07E-08 |
| ENSECAG00000005880 | 8.804705 | 8.25E-10 | 1.65E-08 |
| ENSECAG00000022333 | 8.804705 | 8.25E-10 | 1.65E-08 |
| ENSECAG00000022782 | 8.804705 | 1.05E-09 | 2.07E-08 |
| ENSECAG00000022892 | 8.816751 | 9.67E-10 | 1.93E-08 |
| ENSECAG00000001072 | 8.825123 | 1.46E-09 | 2.83E-08 |
| ENSECAG00000024177 | 8.828697 | 1.46E-09 | 2.83E-08 |
| ENSECAG00000022785 | 8.828697 | 1.05E-09 | 2.07E-08 |
| ENSECAG00000007737 | 8.875516 | 4.83E-10 | 9.82E-09 |
| ENSECAG00000009494 | 8.875516 | 7.63E-10 | 1.53E-08 |
| ENSECAG00000011432 | 8.886988 | 1.14E-09 | 2.24E-08 |
| ENSECAG00000023010 | 8.898368 | 5.61E-10 | 1.13E-08 |
| ENSECAG00000006901 | 8.90966 | 4.83E-10 | 9.82E-09 |
| ENSECAG00000025139 | 8.943014 | 3.87E-10 | 7.94E-09 |
| ENSECAG00000012886 | 8.953963 | 3.12E-10 | 6.51E-09 |
| ENSECAG00000023164 | 9.007495 | 3.6E-10 | 7.42E-09 |
| ENSECAG00000020558 | 9.028364 | 4.83E-10 | 9.82E-09 |
| ENSECAG00000015076 | 9.048935 | 8.75E-11 | 1.89E-09 |
| ENSECAG00000019103 | 9.048935 | 3.87E-10 | 7.94E-09 |
| ENSECAG00000022409 | 9.048935 | 5.2E-10 | 1.06E-08 |
| ENSECAG00000022756 | 9.089217 | 1.46E-10 | 3.08E-09 |
| ENSECAG00000001308 | 9.099115 | 7.72E-11 | 1.69E-09 |
| ENSECAG00000001410 | 9.099115 | 7.72E-11 | 1.69E-09 |
| ENSECAG00000004289 | 9.128406 | 4.49E-11 | 9.99E-10 |
| ENSECAG00000001580 | 9.128406 | 8.22E-11 | 1.79E-09 |
| ENSECAG00000018064 | 9.166558 | 8.22E-11 | 1.79E-09 |
| ENSECAG00000022990 | 9.166558 | 9.32E-11 | 2E-09 |
| ENSECAG00000003748 | 9.17594 | 1.2E-10 | 2.56E-09 |
| ENSECAG00000014508 | 9.17594 | 6.05E-11 | 1.33E-09 |
| ENSECAG00000014497 | 9.212962 | 2.69E-11 | 6.11E-10 |
| ENSECAG00000009133 | 9.257744 | 2.84E-11 | 6.44E-10 |
| ENSECAG00000008644 | 9.257744 | 1.13E-10 | 2.41E-09 |
| ENSECAG00000020155 | 9.266553 | 6.05E-11 | 1.33E-09 |
| ENSECAG00000006080 | 9.284013 | 2.4E-11 | 5.5E-10 |
| ENSECAG00000019874 | 9.284013 | 3.77E-11 | 8.48E-10 |
| ENSECAG00000024665 | 9.292664 | 4E-11 | 8.94E-10 |
| ENSECAG00000024767 | 9.292664 | 5.36E-11 | 1.19E-09 |
| ENSECAG00000002414 | 9.309812 | 1.93E-11 | 4.46E-10 |
| ENSECAG00000014289 | 9.309812 | 3.56E-11 | 8.02E-10 |
| ENSECAG00000009394 | 9.309812 | 4.49E-11 | 9.99E-10 |
| ENSECAG00000003862 | 9.35181 | 3E-11 | 6.79E-10 |
| ENSECAG00000019917 | 9.368273 | 4E-11 | 8.94E-10 |
| ENSECAG00000007205 | 9.416564 | 2.84E-11 | 6.44E-10 |
| ENSECAG00000008020 | 9.440351 | 1.41E-11 | 3.28E-10 |
| ENSECAG00000021396 | 9.463291 | 8.9E-12 | 2.12E-10 |
| ENSECAG00000022601 | 9.470934 | 9.36E-12 | 2.22E-10 |
| ENSECAG00000017301 | 9.478536 | 2.54E-11 | 5.79E-10 |
| ENSECAG00000020761 | 9.493622 | 9.84E-12 | 2.33E-10 |
| ENSECAG00000004119 | 9.515959 | 1.15E-11 | 2.68E-10 |
| ENSECAG00000007032 | 9.515959 | 8.06E-12 | 1.93E-10 |
| ENSECAG00000012066 | 9.530661 | 1.03E-11 | 2.44E-10 |
| ENSECAG00000024823 | 9.532999 | 1.03E-11 | 2.44E-10 |
| ENSECAG00000002519 | 9.537956 | 8.06E-12 | 1.93E-10 |
| ENSECAG00000013277 | 9.545214 | 8.47E-12 | 2.02E-10 |
| ENSECAG00000021846 | 9.545214 | 8.9E-12 | 2.12E-10 |
| ENSECAG00000010514 | 9.552436 | 6.96E-12 | 1.68E-10 |
| ENSECAG00000017510 | 9.566772 | 3.3E-12 | 8.2E-11 |
| ENSECAG00000015772 | 9.580967 | 1.09E-11 | 2.56E-10 |
| ENSECAG00000003314 | 9.602002 | 4.54E-12 | 1.12E-10 |
| ENSECAG00000015525 | 9.622734 | 4.76E-12 | 1.16E-10 |
| ENSECAG00000015905 | 9.643172 | 1.03E-11 | 2.44E-10 |
| ENSECAG00000020627 | 9.656639 | 4.76E-12 | 1.16E-10 |
| ENSECAG00000023915 | 9.66998 | 6.03E-12 | 1.46E-10 |
| ENSECAG00000009401 | 9.683134 | 2.22E-12 | 5.67E-11 |
| ENSECAG00000014542 | 9.689765 | 3.78E-12 | 9.36E-11 |
| ENSECAG00000007856 | 9.702805 | 2.22E-12 | 5.67E-11 |
| ENSECAG00000006861 | 9.715729 | 3.3E-12 | 8.2E-11 |
| ENSECAG00000022160 | 9.722147 | 2.76E-12 | 6.98E-11 |
| ENSECAG00000013285 | 9.766296 | 5.75E-12 | 1.39E-10 |
| ENSECAG00000009893 | 9.778665 | 2.42E-12 | 6.13E-11 |
| ENSECAG00000004874 | 9.79093 | 1.14E-12 | 3E-11 |
| ENSECAG00000017327 | 9.802727 | 2.88E-12 | 7.28E-11 |
| ENSECAG00000010426 | 9.827109 | 2.12E-12 | 5.45E-11 |
| ENSECAG00000024253 | 9.830559 | 1.09E-12 | 2.89E-11 |
| ENSECAG00000008825 | 9.833052 | 3.3E-12 | 8.2E-11 |
| ENSECAG00000014688 | 9.862404 | 8.95E-13 | 2.41E-11 |
| ENSECAG00000024628 | 9.86664 | 1.01E-12 | 2.69E-11 |
| ENSECAG00000011251 | 9.87398 | 1.87E-12 | 4.8E-11 |
| ENSECAG00000010641 | 9.883918 | 4.35E-13 | 1.23E-11 |
| ENSECAG00000011727 | 9.885463 | 9.69E-13 | 2.59E-11 |
| ENSECAG00000024346 | 9.885463 | 1.09E-12 | 2.89E-11 |
| ENSECAG00000002441 | 9.88552 | 4.51E-13 | 1.26E-11 |
| ENSECAG00000009343 | 9.891171 | 2.42E-12 | 6.13E-11 |
| ENSECAG00000005538 | 9.891171 | 1.45E-12 | 3.78E-11 |
| ENSECAG00000006451 | 9.902519 | 5.43E-13 | 1.49E-11 |
| ENSECAG00000012861 | 9.913778 | 1.72E-12 | 4.44E-11 |
| ENSECAG00000019674 | 9.930504 | 3.9E-13 | 1.11E-11 |
| ENSECAG00000020471 | 9.930504 | 1.79E-12 | 4.61E-11 |
| ENSECAG00000013578 | 9.936037 | 1.18E-12 | 3.11E-11 |
| ENSECAG00000001128 | 9.952508 | 6.32E-13 | 1.72E-11 |
| ENSECAG00000016690 | 9.979549 | 5.64E-13 | 1.54E-11 |
| ENSECAG00000013246 | 9.995534 | 4.86E-13 | 1.35E-11 |
| ENSECAG00000001856 | 10.00609 | 8.95E-13 | 2.41E-11 |
| ENSECAG00000005098 | 10.01134 | 5.04E-13 | 1.4E-11 |
| ENSECAG00000011164 | 10.01532 | 3.5E-13 | 1E-11 |
| ENSECAG00000024958 | 10.02174 | 3.63E-13 | 1.03E-11 |
| ENSECAG00000022977 | 10.02698 | 4.86E-13 | 1.35E-11 |
| ENSECAG00000008471 | 10.03731 | 3.14E-13 | 9.06E-12 |
| ENSECAG00000006890 | 10.05776 | 1.53E-13 | 4.55E-12 |
| ENSECAG00000012167 | 10.13339 | 1.69E-13 | 4.99E-12 |
| ENSECAG00000010332 | 10.15585 | 1.53E-13 | 4.55E-12 |
| ENSECAG00000014174 | 10.20709 | 2.3E-13 | 6.75E-12 |
| ENSECAG00000011497 | 10.21166 | 3.26E-13 | 9.36E-12 |
| ENSECAG00000014342 | 10.24769 | 5.31E-14 | 1.68E-12 |
| ENSECAG00000022752 | 10.24769 | 1.53E-13 | 4.55E-12 |
| ENSECAG00000019577 | 10.25657 | 7.86E-14 | 2.4E-12 |
| ENSECAG00000018327 | 10.26098 | 7.39E-14 | 2.27E-12 |
| ENSECAG00000023177 | 10.26977 | 1.64E-13 | 4.84E-12 |
| ENSECAG00000013971 | 10.3044 | 8.11E-14 | 2.47E-12 |
| ENSECAG00000011148 | 10.30765 | 6.96E-14 | 2.15E-12 |
| ENSECAG00000008499 | 10.32141 | 1.44E-13 | 4.3E-12 |
| ENSECAG00000018279 | 10.32984 | 5.8E-14 | 1.83E-12 |
| ENSECAG00000020788 | 10.36718 | 3.75E-14 | 1.21E-12 |
| ENSECAG00000023862 | 10.37127 | 2.27E-14 | 7.56E-13 |
| ENSECAG00000000616 | 10.37942 | 4.58E-14 | 1.46E-12 |
| ENSECAG00000022294 | 10.39155 | 2.75E-14 | 9.05E-13 |
| ENSECAG00000025052 | 10.39155 | 4.33E-14 | 1.38E-12 |
| ENSECAG00000021914 | 10.39557 | 5.15E-14 | 1.63E-12 |
| ENSECAG00000015600 | 10.39958 | 5.98E-14 | 1.88E-12 |
| ENSECAG00000002748 | 10.39958 | 6.35E-14 | 1.98E-12 |
| ENSECAG00000023097 | 10.40577 | 3.54E-14 | 1.15E-12 |
| ENSECAG00000023651 | 10.40757 | 2.99E-14 | 9.81E-13 |
| ENSECAG00000022151 | 10.41551 | 3.75E-14 | 1.21E-12 |
| ENSECAG00000020347 | 10.45843 | 3.97E-14 | 1.27E-12 |
| ENSECAG00000023132 | 10.53779 | 2.33E-14 | 7.71E-13 |
| ENSECAG00000008692 | 10.55147 | 1.06E-14 | 3.68E-13 |
| ENSECAG00000022737 | 10.56582 | 1.12E-14 | 3.85E-13 |
| ENSECAG00000003237 | 10.57294 | 7.53E-15 | 2.68E-13 |
| ENSECAG00000016332 | 10.58003 | 9.63E-15 | 3.36E-13 |
| ENSECAG00000022070 | 10.59059 | 8.1E-15 | 2.87E-13 |
| ENSECAG00000008310 | 10.65239 | 9.39E-15 | 3.28E-13 |
| ENSECAG00000012050 | 10.65909 | 5.26E-15 | 1.91E-13 |
| ENSECAG00000007880 | 10.67241 | 1.15E-14 | 3.93E-13 |
| ENSECAG00000008301 | 10.68232 | 9.39E-15 | 3.28E-13 |
| ENSECAG00000020921 | 10.69947 | 2.35E-15 | 8.95E-14 |
| ENSECAG00000000690 | 10.71165 | 4.18E-15 | 1.54E-13 |
| ENSECAG00000017101 | 10.72769 | 4.91E-15 | 1.79E-13 |
| ENSECAG00000007911 | 10.73278 | 5.26E-15 | 1.91E-13 |
| ENSECAG00000024063 | 10.74039 | 3.26E-15 | 1.22E-13 |
| ENSECAG00000018310 | 10.75627 | 1.33E-15 | 5.21E-14 |
| ENSECAG00000022163 | 10.77167 | 1.86E-15 | 7.14E-14 |
| ENSECAG00000021005 | 10.77476 | 1.33E-15 | 5.21E-14 |
| ENSECAG00000009635 | 10.78706 | 1.45E-15 | 5.62E-14 |
| ENSECAG00000021840 | 10.78706 | 1.45E-15 | 5.62E-14 |
| ENSECAG00000017737 | 10.79621 | 2.25E-15 | 8.6E-14 |
| ENSECAG00000000555 | 10.81732 | 2.61E-15 | 9.95E-14 |
| ENSECAG00000003959 | 10.87897 | 2.06E-15 | 7.92E-14 |
| ENSECAG00000000689 | 10.9353 | 8.94E-16 | 3.59E-14 |
| ENSECAG00000013242 | 10.95451 | 9.11E-16 | 3.65E-14 |
| ENSECAG00000009021 | 10.95584 | 2.85E-16 | 1.25E-14 |
| ENSECAG00000008875 | 10.96653 | 6.95E-16 | 2.83E-14 |
| ENSECAG00000013735 | 10.98419 | 3.99E-16 | 1.7E-14 |
| ENSECAG00000010557 | 10.98952 | 3.39E-16 | 1.46E-14 |
| ENSECAG00000015739 | 11.01849 | 7.09E-16 | 2.87E-14 |
| ENSECAG00000001415 | 11.02109 | 7.5E-16 | 3.04E-14 |
| ENSECAG00000002264 | 11.02888 | 5.24E-16 | 2.16E-14 |
| ENSECAG00000003990 | 11.03405 | 5.15E-16 | 2.13E-14 |
| ENSECAG00000023039 | 11.06432 | 6.2E-16 | 2.54E-14 |
| ENSECAG00000005811 | 11.10453 | 3E-16 | 1.3E-14 |
| ENSECAG00000021172 | 11.10943 | 4.36E-16 | 1.85E-14 |
| ENSECAG00000005263 | 11.12196 | 4.96E-16 | 2.06E-14 |
| ENSECAG00000024306 | 11.12889 | 2.9E-16 | 1.27E-14 |
| ENSECAG00000016767 | 11.17407 | 1.74E-16 | 7.85E-15 |
| ENSECAG00000017871 | 11.17972 | 1.55E-16 | 7.09E-15 |
| ENSECAG00000010657 | 11.21105 | 1.48E-16 | 6.78E-15 |
| ENSECAG00000012556 | 11.21333 | 1.2E-16 | 5.6E-15 |
| ENSECAG00000002002 | 11.25154 | 1.74E-16 | 7.85E-15 |
| ENSECAG00000017053 | 11.32271 | 9.75E-17 | 4.61E-15 |
| ENSECAG00000016580 | 11.33138 | 6.26E-17 | 3E-15 |
| ENSECAG00000011502 | 11.4675 | 1.02E-16 | 4.81E-15 |
| ENSECAG00000014069 | 11.49212 | 1.27E-17 | 6.65E-16 |
| ENSECAG00000013538 | 11.54558 | 5.4E-17 | 2.6E-15 |
| ENSECAG00000019720 | 11.59087 | 8.21E-18 | 4.41E-16 |
| ENSECAG00000002707 | 11.60066 | 2.51E-17 | 1.27E-15 |
| ENSECAG00000008307 | 11.60929 | 5.81E-18 | 3.26E-16 |
| ENSECAG00000024130 | 11.6265 | 9.06E-18 | 4.81E-16 |
| ENSECAG00000009095 | 11.66034 | 2.76E-18 | 1.63E-16 |
| ENSECAG00000005459 | 11.66532 | 5.95E-18 | 3.32E-16 |
| ENSECAG00000011742 | 11.67031 | 5.23E-18 | 2.99E-16 |
| ENSECAG00000008807 | 11.67694 | 7.45E-18 | 4.06E-16 |
| ENSECAG00000023620 | 11.71766 | 1.52E-17 | 7.83E-16 |
| ENSECAG00000012953 | 11.74155 | 3.67E-18 | 2.13E-16 |
| ENSECAG00000007605 | 11.76505 | 5.17E-18 | 2.96E-16 |
| ENSECAG00000016022 | 11.77589 | 3.07E-18 | 1.8E-16 |
| ENSECAG00000010540 | 11.79885 | 1.53E-18 | 9.69E-17 |
| ENSECAG00000015502 | 11.92662 | 2.22E-18 | 1.37E-16 |
| ENSECAG00000022735 | 11.93631 | 1.46E-18 | 9.35E-17 |
| ENSECAG00000003830 | 11.95387 | 5.01E-19 | 3.44E-17 |
| ENSECAG00000017980 | 11.9612 | 4.74E-19 | 3.27E-17 |
| ENSECAG00000011751 | 11.96366 | 3.07E-18 | 1.8E-16 |
| ENSECAG00000014125 | 11.97982 | 2.42E-18 | 1.47E-16 |
| ENSECAG00000009719 | 12.01422 | 3.89E-19 | 2.79E-17 |
| ENSECAG00000008317 | 12.02854 | 8.32E-19 | 5.53E-17 |
| ENSECAG00000022117 | 12.03489 | 5.39E-19 | 3.66E-17 |
| ENSECAG00000015317 | 12.10193 | 3.15E-19 | 2.29E-17 |
| ENSECAG00000010369 | 12.10529 | 2.04E-19 | 1.57E-17 |
| ENSECAG00000018101 | 12.14179 | 3.38E-19 | 2.45E-17 |
| ENSECAG00000021004 | 12.23674 | 1.05E-19 | 8.62E-18 |
| ENSECAG00000015820 | 12.25014 | 2.09E-19 | 1.58E-17 |
| ENSECAG00000022396 | 12.31951 | 2.41E-19 | 1.78E-17 |
| ENSECAG00000008800 | 12.36123 | 8.39E-20 | 6.98E-18 |
| ENSECAG00000017351 | 12.36123 | 4.81E-20 | 4.13E-18 |
| ENSECAG00000022273 | 12.36739 | 8.45E-20 | 6.99E-18 |
| ENSECAG00000023274 | 12.40378 | 8.32E-20 | 6.97E-18 |
| ENSECAG00000004096 | 12.40975 | 1.33E-19 | 1.07E-17 |
| ENSECAG00000008445 | 12.41274 | 2.01E-20 | 1.84E-18 |
| ENSECAG00000004830 | 12.43342 | 6.49E-20 | 5.51E-18 |
| ENSECAG00000023779 | 12.44607 | 3.44E-20 | 3.06E-18 |
| ENSECAG00000017678 | 12.4815 | 1.39E-20 | 1.31E-18 |
| ENSECAG00000016583 | 12.53228 | 1.6E-20 | 1.5E-18 |
| ENSECAG00000014725 | 12.63797 | 9E-21 | 8.86E-19 |
| ENSECAG00000013839 | 12.65508 | 7.33E-21 | 7.39E-19 |
| ENSECAG00000016082 | 12.70551 | 1.85E-20 | 1.7E-18 |
| ENSECAG00000013823 | 12.70777 | 1.16E-20 | 1.11E-18 |
| ENSECAG00000010266 | 12.76173 | 3.36E-21 | 3.64E-19 |
| ENSECAG00000009035 | 12.76745 | 5.79E-21 | 6.03E-19 |
| ENSECAG00000002097 | 12.79484 | 6.1E-21 | 6.29E-19 |
| ENSECAG00000022647 | 12.97629 | 5.96E-22 | 7.12E-20 |
| ENSECAG00000014848 | 12.98231 | 6.02E-22 | 7.12E-20 |
| ENSECAG00000023663 | 12.98898 | 5.96E-22 | 7.12E-20 |
| ENSECAG00000013579 | 13.03433 | 8.01E-22 | 9.4E-20 |
| ENSECAG00000019482 | 13.21911 | 1.74E-22 | 2.24E-20 |
| ENSECAG00000008121 | 13.39871 | 4.64E-23 | 6.96E-21 |
| ENSECAG00000015474 | 13.41621 | 1.06E-22 | 1.44E-20 |
| ENSECAG00000012712 | 13.42075 | 6.16E-23 | 9.03E-21 |
| ENSECAG00000017677 | 13.61197 | 1.19E-23 | 2.08E-21 |
| ENSECAG00000012580 | 13.67491 | 1.24E-23 | 2.13E-21 |
| ENSECAG00000014042 | 13.74962 | 2.6E-24 | 5.25E-22 |
| ENSECAG00000012692 | 13.80347 | 6.8E-24 | 1.31E-21 |
| ENSECAG00000012230 | 13.82189 | 9.88E-24 | 1.75E-21 |
| ENSECAG00000013024 | 13.83592 | 7.4E-24 | 1.33E-21 |
| ENSECAG00000021056 | 13.89961 | 7.15E-24 | 1.33E-21 |
| ENSECAG00000002843 | 13.93189 | 1.37E-24 | 2.81E-22 |
| ENSECAG00000000575 | 13.99523 | 1.01E-24 | 2.1E-22 |
| ENSECAG00000017946 | 14.01432 | 3.73E-25 | 9.45E-23 |
| ENSECAG00000004412 | 14.01932 | 5.26E-25 | 1.26E-22 |
| ENSECAG00000004780 | 14.0645 | 6.39E-25 | 1.45E-22 |
| ENSECAG00000003722 | 14.13602 | 7.05E-25 | 1.54E-22 |
| ENSECAG00000006230 | 14.22223 | 4.28E-25 | 1.06E-22 |
| ENSECAG00000020670 | 14.30971 | 1.82E-25 | 4.99E-23 |
| ENSECAG00000000562 | 14.34222 | 8.97E-26 | 2.57E-23 |
| ENSECAG00000014448 | 14.35707 | 8.17E-26 | 2.45E-23 |
| ENSECAG00000024012 | 14.41477 | 5.45E-26 | 1.72E-23 |
| ENSECAG00000016471 | 14.45311 | 3.9E-26 | 1.29E-23 |
| ENSECAG00000023567 | 14.55389 | 1.13E-26 | 4.16E-24 |
| ENSECAG00000016038 | 14.68425 | 1.59E-26 | 5.69E-24 |
| ENSECAG00000016405 | 14.80328 | 1.98E-27 | 8.79E-25 |
| ENSECAG00000020596 | 14.90318 | 5.19E-27 | 1.97E-24 |
| ENSECAG00000003007 | 15.01846 | 1.57E-27 | 7.25E-25 |
| ENSECAG00000016786 | 15.14211 | 1.8E-28 | 9.28E-26 |
| ENSECAG00000002814 | 15.14271 | 3.16E-28 | 1.57E-25 |
| ENSECAG00000024844 | 15.36983 | 4.29E-29 | 2.41E-26 |
| ENSECAG00000016995 | 15.38217 | 1.21E-28 | 6.53E-26 |
| ENSECAG00000016362 | 15.38692 | 3.02E-29 | 1.77E-26 |
| ENSECAG00000022274 | 15.66579 | 4.2E-30 | 3.01E-27 |
| ENSECAG00000021009 | 15.84649 | 4.94E-30 | 3.36E-27 |
| ENSECAG00000015339 | 16.62777 | 1.58E-32 | 1.36E-29 |
| ENSECAG00000015968 | 17.07079 | 1.9E-33 | 1.75E-30 |
| ENSECAG00000011505 | 17.52678 | 1.84E-35 | 2.96E-32 |
| ENSECAG00000017499 | 18.15507 | 5.46E-37 | 1.17E-33 |
| ENSECAG00000009390 | 19.39039 | 1.6E-40 | 5.16E-37 |
| ENSECAG00000014949 | 21.87683 | 3.29E-48 | 1.41E-44 |

(b) Blood

| Ens id | logFC | PValue | FDR |
| --- | --- | --- | --- |
| ENSECAG00000012811 | -18.0787 | 1.33E-36 | 1.71E-32 |
| ENSECAG00000022681 | -17.8301 | 2.35E-36 | 1.71E-32 |
| ENSECAG00000015492 | 17.81983 | 5.16E-36 | 2.5E-32 |
| ENSECAG00000008134 | -17.6252 | 8.55E-36 | 3.1E-32 |
| ENSECAG00000019222 | 17.66888 | 1.07E-35 | 3.12E-32 |
| ENSECAG00000008849 | -17.3299 | 5.22E-35 | 1.26E-31 |
| ENSECAG00000012763 | -17.3557 | 1.18E-34 | 2.45E-31 |
| ENSECAG00000015925 | -17.23 | 1.81E-34 | 2.94E-31 |
| ENSECAG00000020056 | 17.27839 | 1.82E-34 | 2.94E-31 |
| ENSECAG00000004535 | -16.9023 | 1.69E-33 | 2.45E-30 |
| ENSECAG00000012737 | 16.84539 | 3.45E-33 | 4.55E-30 |
| ENSECAG00000008475 | -16.724 | 1.37E-32 | 1.65E-29 |
| ENSECAG00000012941 | -16.7123 | 1.56E-32 | 1.74E-29 |
| ENSECAG00000003971 | -16.5801 | 1.97E-32 | 2.04E-29 |
| ENSECAG00000013587 | 16.5166 | 3.31E-32 | 3.21E-29 |
| ENSECAG00000002754 | 16.50583 | 3.55E-32 | 3.22E-29 |
| ENSECAG00000005188 | 16.45611 | 5.22E-32 | 4.46E-29 |
| ENSECAG00000009905 | -16.3297 | 7.62E-32 | 5.27E-29 |
| ENSECAG00000020637 | -16.3107 | 7.55E-32 | 5.27E-29 |
| ENSECAG00000018947 | 16.38298 | 7.26E-32 | 5.27E-29 |
| ENSECAG00000017599 | 16.37556 | 9.76E-32 | 6.44E-29 |
| ENSECAG00000011439 | 16.2255 | 2.72E-31 | 1.72E-28 |
| ENSECAG00000008164 | -16.153 | 4.48E-31 | 2.71E-28 |
| ENSECAG00000010702 | -16.06 | 7.87E-31 | 4.58E-28 |
| ENSECAG00000007374 | 16.03314 | 8.44E-31 | 4.71E-28 |
| ENSECAG00000017539 | -15.9671 | 2.22E-30 | 1.19E-27 |
| ENSECAG00000000040 | 15.88099 | 2.66E-30 | 1.38E-27 |
| ENSECAG00000020559 | 15.74404 | 5.87E-30 | 2.94E-27 |
| ENSECAG00000005802 | -15.6987 | 6.78E-30 | 3.28E-27 |
| ENSECAG00000009318 | -15.7832 | 7.11E-30 | 3.33E-27 |
| ENSECAG00000014933 | 15.7038 | 7.7E-30 | 3.5E-27 |
| ENSECAG00000014823 | 15.72048 | 8.16E-30 | 3.59E-27 |
| ENSECAG00000015323 | -15.7219 | 9.8E-30 | 4.19E-27 |
| ENSECAG00000012597 | -15.7503 | 1.12E-29 | 4.52E-27 |
| ENSECAG00000013101 | -15.7445 | 1.11E-29 | 4.52E-27 |
| ENSECAG00000021002 | 15.6373 | 1.46E-29 | 5.59E-27 |
| ENSECAG00000020855 | 15.59078 | 1.86E-29 | 6.92E-27 |
| ENSECAG00000014302 | 15.51146 | 2.96E-29 | 1.08E-26 |
| ENSECAG00000025008 | 15.5202 | 3.24E-29 | 1.15E-26 |
| ENSECAG00000013024 | 15.49255 | 4.01E-29 | 1.39E-26 |
| ENSECAG00000013058 | -15.4072 | 4.2E-29 | 1.42E-26 |
| ENSECAG00000021996 | -15.4493 | 5.05E-29 | 1.67E-26 |
| ENSECAG00000012874 | 15.43339 | 5.39E-29 | 1.74E-26 |
| ENSECAG00000024965 | -15.2732 | 8.89E-29 | 2.81E-26 |
| ENSECAG00000011954 | 15.2507 | 1.75E-28 | 5.4E-26 |
| ENSECAG00000009648 | -15.2415 | 2.45E-28 | 7.41E-26 |
| ENSECAG00000004308 | -15.2539 | 2.51E-28 | 7.45E-26 |
| ENSECAG00000010964 | -15.2879 | 2.65E-28 | 7.7E-26 |
| ENSECAG00000008028 | 15.17052 | 3.09E-28 | 8.8E-26 |
| ENSECAG00000012735 | -15.0038 | 6.08E-28 | 1.67E-25 |
| ENSECAG00000014568 | -15.0454 | 6.4E-28 | 1.72E-25 |
| ENSECAG00000002694 | -15.0967 | 7.27E-28 | 1.92E-25 |
| ENSECAG00000018229 | 15.06013 | 7.52E-28 | 1.95E-25 |
| ENSECAG00000021273 | 14.99624 | 1.08E-27 | 2.76E-25 |
| ENSECAG00000011962 | 14.95283 | 1.57E-27 | 3.93E-25 |
| ENSECAG00000015075 | 14.74025 | 6.67E-27 | 1.64E-24 |
| ENSECAG00000009609 | 14.71534 | 7.32E-27 | 1.77E-24 |
| ENSECAG00000022807 | -14.7189 | 8.94E-27 | 2.09E-24 |
| ENSECAG00000003615 | -14.6929 | 9.83E-27 | 2.27E-24 |
| ENSECAG00000016283 | -14.5988 | 2.23E-26 | 5.06E-24 |
| ENSECAG00000015067 | 14.53292 | 2.68E-26 | 5.98E-24 |
| ENSECAG00000003796 | -14.5219 | 2.99E-26 | 6.58E-24 |
| ENSECAG00000018789 | -14.4497 | 3.41E-26 | 7.4E-24 |
| ENSECAG00000004539 | -14.4277 | 5.38E-26 | 1.15E-23 |
| ENSECAG00000001102 | -14.4182 | 5.81E-26 | 1.22E-23 |
| ENSECAG00000012357 | -14.4134 | 6.3E-26 | 1.29E-23 |
| ENSECAG00000013921 | -14.3988 | 6.27E-26 | 1.29E-23 |
| ENSECAG00000002341 | -14.4001 | 6.83E-26 | 1.38E-23 |
| ENSECAG00000002641 | -14.3886 | 7.4E-26 | 1.47E-23 |
| ENSECAG00000023594 | -14.3383 | 1.1E-25 | 2.16E-23 |
| ENSECAG00000001477 | -14.319 | 1.18E-25 | 2.29E-23 |
| ENSECAG00000001707 | -14.269 | 1.35E-25 | 2.54E-23 |
| ENSECAG00000007950 | 14.26299 | 1.34E-25 | 2.54E-23 |
| ENSECAG00000000872 | -14.2221 | 1.38E-25 | 2.57E-23 |
| ENSECAG00000019878 | -14.3445 | 1.42E-25 | 2.61E-23 |
| ENSECAG00000017002 | 14.29898 | 1.44E-25 | 2.61E-23 |
| ENSECAG00000019538 | -14.2578 | 1.5E-25 | 2.68E-23 |
| ENSECAG00000000983 | -14.2783 | 1.57E-25 | 2.77E-23 |
| ENSECAG00000003294 | -14.2491 | 1.68E-25 | 2.95E-23 |
| ENSECAG00000005704 | -14.2532 | 2.01E-25 | 3.43E-23 |
| ENSECAG00000017276 | -14.2493 | 1.99E-25 | 3.43E-23 |
| ENSECAG00000001974 | -14.1696 | 3.07E-25 | 5.19E-23 |
| ENSECAG00000002880 | -14.149 | 3.31E-25 | 5.53E-23 |
| ENSECAG00000004206 | 14.0931 | 4.19E-25 | 6.91E-23 |
| ENSECAG00000005578 | 14.04977 | 5.65E-25 | 9.22E-23 |
| ENSECAG00000005370 | 14.04679 | 5.75E-25 | 9.28E-23 |
| ENSECAG00000012010 | -14.1033 | 5.94E-25 | 9.38E-23 |
| ENSECAG00000007768 | -13.9544 | 5.9E-25 | 9.38E-23 |
| ENSECAG00000000771 | 14.02658 | 6.48E-25 | 1.01E-22 |
| ENSECAG00000003712 | -14.0696 | 7.16E-25 | 1.1E-22 |
| ENSECAG00000005008 | 14.01394 | 7.18E-25 | 1.1E-22 |
| ENSECAG00000007515 | -14.0543 | 7.39E-25 | 1.11E-22 |
| ENSECAG00000004533 | 14.02686 | 7.39E-25 | 1.11E-22 |
| ENSECAG00000019992 | -13.992 | 9.46E-25 | 1.4E-22 |
| ENSECAG00000000175 | -14.0605 | 9.92E-25 | 1.46E-22 |
| ENSECAG00000016379 | 13.94331 | 1.31E-24 | 1.9E-22 |
| ENSECAG00000024280 | 13.89827 | 1.41E-24 | 2.03E-22 |
| ENSECAG00000021610 | -13.9333 | 2.21E-24 | 3.15E-22 |
| ENSECAG00000000867 | 13.86299 | 2.32E-24 | 3.28E-22 |
| ENSECAG00000024834 | 13.84409 | 2.56E-24 | 3.57E-22 |
| ENSECAG00000001571 | -13.8527 | 2.66E-24 | 3.68E-22 |
| ENSECAG00000016841 | -13.9264 | 2.75E-24 | 3.77E-22 |
| ENSECAG00000003744 | 13.78314 | 3.45E-24 | 4.68E-22 |
| ENSECAG00000009314 | -13.8285 | 3.71E-24 | 5E-22 |
| ENSECAG00000001107 | -13.7585 | 4.93E-24 | 6.57E-22 |
| ENSECAG00000000849 | -13.7468 | 5.31E-24 | 7.01E-22 |
| ENSECAG00000018585 | 13.73641 | 5.74E-24 | 7.51E-22 |
| ENSECAG00000015107 | 13.71344 | 6.08E-24 | 7.89E-22 |
| ENSECAG00000002367 | 13.67836 | 7E-24 | 8.92E-22 |
| ENSECAG00000002670 | 13.67836 | 7E-24 | 8.92E-22 |
| ENSECAG00000002957 | 13.65072 | 8.47E-24 | 1.07E-21 |
| ENSECAG00000005853 | -13.6807 | 1.27E-23 | 1.59E-21 |
| ENSECAG00000012409 | 13.60168 | 1.45E-23 | 1.8E-21 |
| ENSECAG00000019775 | 13.58772 | 1.6E-23 | 1.97E-21 |
| ENSECAG00000014722 | -13.589 | 1.63E-23 | 1.99E-21 |
| ENSECAG00000024142 | -13.5152 | 1.86E-23 | 2.25E-21 |
| ENSECAG00000006127 | 13.56188 | 1.88E-23 | 2.26E-21 |
| ENSECAG00000004301 | 13.52349 | 2.19E-23 | 2.61E-21 |
| ENSECAG00000023922 | 13.49924 | 2.35E-23 | 2.78E-21 |
| ENSECAG00000002275 | -13.5537 | 2.44E-23 | 2.86E-21 |
| ENSECAG00000007106 | -13.537 | 2.53E-23 | 2.94E-21 |
| ENSECAG00000023057 | 13.4852 | 2.95E-23 | 3.4E-21 |
| ENSECAG00000013203 | -13.5348 | 3.16E-23 | 3.61E-21 |
| ENSECAG00000008489 | 13.48276 | 3.21E-23 | 3.65E-21 |
| ENSECAG00000001637 | 13.45132 | 3.27E-23 | 3.68E-21 |
| ENSECAG00000001576 | 13.44831 | 3.34E-23 | 3.73E-21 |
| ENSECAG00000001364 | 13.42809 | 3.83E-23 | 4.22E-21 |
| ENSECAG00000003149 | 13.45824 | 3.82E-23 | 4.22E-21 |
| ENSECAG00000014256 | 13.44783 | 3.97E-23 | 4.34E-21 |
| ENSECAG00000024691 | -13.4448 | 5E-23 | 5.42E-21 |
| ENSECAG00000006089 | 13.3742 | 6.18E-23 | 6.6E-21 |
| ENSECAG00000015271 | 13.31967 | 1.05E-22 | 1.11E-20 |
| ENSECAG00000000700 | -13.2185 | 1.14E-22 | 1.2E-20 |
| ENSECAG00000005191 | 13.27971 | 1.21E-22 | 1.27E-20 |
| ENSECAG00000001143 | -13.2407 | 2.57E-22 | 2.63E-20 |
| ENSECAG00000003206 | -13.1965 | 2.56E-22 | 2.63E-20 |
| ENSECAG00000015293 | -13.1317 | 2.83E-22 | 2.88E-20 |
| ENSECAG00000008976 | 13.13539 | 3.17E-22 | 3.2E-20 |
| ENSECAG00000019424 | 13.13288 | 3.3E-22 | 3.3E-20 |
| ENSECAG00000011014 | -13.0543 | 4.87E-22 | 4.85E-20 |
| ENSECAG00000020996 | 13.05634 | 6.16E-22 | 6.09E-20 |
| ENSECAG00000012129 | -13.1118 | 6.63E-22 | 6.51E-20 |
| ENSECAG00000023631 | 12.98368 | 8.13E-22 | 7.93E-20 |
| ENSECAG00000011655 | -12.8641 | 8.45E-22 | 8.18E-20 |
| ENSECAG00000000689 | -12.9826 | 8.86E-22 | 8.53E-20 |
| ENSECAG00000023952 | -12.9812 | 1.14E-21 | 1.09E-19 |
| ENSECAG00000010206 | -12.8442 | 1.29E-21 | 1.22E-19 |
| ENSECAG00000001534 | 12.88833 | 1.81E-21 | 1.71E-19 |
| ENSECAG00000010679 | -12.8203 | 1.98E-21 | 1.85E-19 |
| ENSECAG00000009962 | -12.8729 | 2.08E-21 | 1.94E-19 |
| ENSECAG00000003907 | -12.9266 | 2.17E-21 | 2.01E-19 |
| ENSECAG00000009830 | -12.7753 | 2.56E-21 | 2.36E-19 |
| ENSECAG00000001344 | 12.80629 | 2.6E-21 | 2.38E-19 |
| ENSECAG00000023779 | -12.766 | 3.13E-21 | 2.84E-19 |
| ENSECAG00000000871 | 12.81789 | 3.33E-21 | 3E-19 |
| ENSECAG00000022720 | 8.674148 | 3.9E-21 | 3.49E-19 |
| ENSECAG00000022068 | -12.7006 | 4.23E-21 | 3.77E-19 |
| ENSECAG00000015880 | -12.7032 | 4.32E-21 | 3.83E-19 |
| ENSECAG00000004573 | 12.7488 | 4.73E-21 | 4.17E-19 |
| ENSECAG00000013736 | -12.7754 | 4.81E-21 | 4.21E-19 |
| ENSECAG00000013857 | 12.73981 | 5.22E-21 | 4.54E-19 |
| ENSECAG00000024126 | -12.6525 | 5.96E-21 | 5.16E-19 |
| ENSECAG00000007717 | -12.5879 | 6.55E-21 | 5.63E-19 |
| ENSECAG00000024391 | -12.7633 | 6.63E-21 | 5.66E-19 |
| ENSECAG00000003164 | -12.735 | 6.71E-21 | 5.7E-19 |
| ENSECAG00000019358 | -12.7131 | 8.89E-21 | 7.51E-19 |
| ENSECAG00000019597 | -12.6132 | 1.02E-20 | 8.54E-19 |
| ENSECAG00000014022 | -12.4995 | 1.27E-20 | 1.06E-18 |
| ENSECAG00000016597 | 12.56903 | 1.47E-20 | 1.22E-18 |
| ENSECAG00000021155 | 12.42183 | 3.88E-20 | 3.2E-18 |
| ENSECAG00000010147 | -12.3838 | 5.98E-20 | 4.91E-18 |
| ENSECAG00000010127 | 12.37929 | 6.12E-20 | 4.99E-18 |
| ENSECAG00000023673 | -12.3722 | 6.3E-20 | 5.11E-18 |
| ENSECAG00000003982 | 12.28683 | 8.32E-20 | 6.72E-18 |
| ENSECAG00000001342 | 12.31373 | 8.52E-20 | 6.84E-18 |
| ENSECAG00000001040 | 12.28269 | 9.13E-20 | 7.29E-18 |
| ENSECAG00000000641 | 12.29138 | 9.94E-20 | 7.89E-18 |
| ENSECAG00000009937 | -12.2372 | 1.29E-19 | 1.02E-17 |
| ENSECAG00000006508 | -12.2971 | 1.78E-19 | 1.39E-17 |
| ENSECAG00000018657 | -12.1666 | 1.83E-19 | 1.42E-17 |
| ENSECAG00000009700 | 12.20373 | 1.89E-19 | 1.46E-17 |
| ENSECAG00000000423 | 12.13804 | 2.41E-19 | 1.85E-17 |
| ENSECAG00000004897 | -12.091 | 2.62E-19 | 2.01E-17 |
| ENSECAG00000021142 | 12.15536 | 2.74E-19 | 2.08E-17 |
| ENSECAG00000001400 | -12.0559 | 3.44E-19 | 2.6E-17 |
| ENSECAG00000023930 | -12.0354 | 3.47E-19 | 2.61E-17 |
| ENSECAG00000007229 | 12.0788 | 3.59E-19 | 2.69E-17 |
| ENSECAG00000010258 | -12.1519 | 3.65E-19 | 2.72E-17 |
| ENSECAG00000005030 | -12.0621 | 4.29E-19 | 3.18E-17 |
| ENSECAG00000010793 | 12.03536 | 5.54E-19 | 4.09E-17 |
| ENSECAG00000006892 | -12.0662 | 6.03E-19 | 4.42E-17 |
| ENSECAG00000007103 | 12.03488 | 6.08E-19 | 4.44E-17 |
| ENSECAG00000021891 | -11.9493 | 6.32E-19 | 4.59E-17 |
| ENSECAG00000016635 | 11.95589 | 9.72E-19 | 7.02E-17 |
| ENSECAG00000012795 | -11.982 | 1.05E-18 | 7.52E-17 |
| ENSECAG00000009202 | 11.9488 | 1.05E-18 | 7.52E-17 |
| ENSECAG00000025089 | 11.93354 | 1.18E-18 | 8.43E-17 |
| ENSECAG00000022159 | -11.8756 | 1.64E-18 | 1.16E-16 |
| ENSECAG00000003254 | 11.829 | 2.55E-18 | 1.8E-16 |
| ENSECAG00000018708 | -11.8111 | 2.85E-18 | 2E-16 |
| ENSECAG00000010436 | -11.7273 | 2.91E-18 | 2.03E-16 |
| ENSECAG00000016025 | 11.77778 | 3.11E-18 | 2.16E-16 |
| ENSECAG00000020631 | -11.7637 | 3.32E-18 | 2.27E-16 |
| ENSECAG00000001684 | -11.7571 | 3.28E-18 | 2.27E-16 |
| ENSECAG00000023280 | -11.6068 | 3.32E-18 | 2.27E-16 |
| ENSECAG00000009275 | 11.78919 | 3.32E-18 | 2.27E-16 |
| ENSECAG00000021619 | 11.7657 | 3.8E-18 | 2.58E-16 |
| ENSECAG00000023809 | 11.70264 | 4.61E-18 | 3.11E-16 |
| ENSECAG00000020367 | -11.719 | 5.29E-18 | 3.56E-16 |
| ENSECAG00000004063 | 11.64366 | 7.28E-18 | 4.87E-16 |
| ENSECAG00000001646 | 11.65647 | 7.64E-18 | 5.09E-16 |
| ENSECAG00000021138 | -11.6972 | 8.02E-18 | 5.32E-16 |
| ENSECAG00000000015 | -11.5504 | 1E-17 | 6.6E-16 |
| ENSECAG00000014169 | 11.60157 | 1.02E-17 | 6.74E-16 |
| ENSECAG00000015527 | 11.5724 | 1.44E-17 | 9.44E-16 |
| ENSECAG00000012397 | -11.5603 | 1.62E-17 | 1.06E-15 |
| ENSECAG00000010575 | -11.4647 | 2.14E-17 | 1.37E-15 |
| ENSECAG00000003001 | -11.4264 | 2.14E-17 | 1.37E-15 |
| ENSECAG00000014704 | 11.46339 | 2.88E-17 | 1.85E-15 |
| ENSECAG00000020436 | -11.3302 | 3.93E-17 | 2.5E-15 |
| ENSECAG00000014936 | -11.351 | 4.47E-17 | 2.83E-15 |
| ENSECAG00000002550 | -11.3396 | 7.28E-17 | 4.6E-15 |
| ENSECAG00000014402 | -11.3212 | 1.18E-16 | 7.41E-15 |
| ENSECAG00000014548 | -11.0821 | 1.63E-16 | 1.02E-14 |
| ENSECAG00000006575 | -11.1276 | 2.06E-16 | 1.28E-14 |
| ENSECAG00000010355 | -10.9736 | 3.11E-16 | 1.93E-14 |
| ENSECAG00000008477 | 11.02576 | 4.69E-16 | 2.9E-14 |
| ENSECAG00000022400 | -11.0596 | 5.44E-16 | 3.35E-14 |
| ENSECAG00000006564 | 11.02261 | 5.86E-16 | 3.59E-14 |
| ENSECAG00000016356 | 10.9703 | 6.57E-16 | 4.01E-14 |
| ENSECAG00000019712 | -10.9133 | 6.95E-16 | 4.23E-14 |
| ENSECAG00000010468 | 10.95978 | 8.43E-16 | 5.1E-14 |
| ENSECAG00000005113 | 6.752694 | 9.5E-16 | 5.71E-14 |
| ENSECAG00000003543 | 10.92558 | 9.48E-16 | 5.71E-14 |
| ENSECAG00000006306 | -10.9255 | 1.23E-15 | 7.34E-14 |
| ENSECAG00000020690 | -10.8232 | 1.36E-15 | 8.09E-14 |
| ENSECAG00000011563 | -10.9498 | 1.42E-15 | 8.4E-14 |
| ENSECAG00000019044 | 10.73469 | 3.19E-15 | 1.88E-13 |
| ENSECAG00000000800 | 10.74126 | 3.48E-15 | 2.05E-13 |
| ENSECAG00000012342 | -10.7777 | 4.37E-15 | 2.56E-13 |
| ENSECAG00000002385 | 10.71146 | 4.58E-15 | 2.67E-13 |
| ENSECAG00000022495 | 10.67762 | 5.02E-15 | 2.92E-13 |
| ENSECAG00000015456 | 10.63592 | 7.17E-15 | 4.15E-13 |
| ENSECAG00000005059 | -6.3604 | 1.31E-14 | 7.58E-13 |
| ENSECAG00000019870 | 10.48844 | 1.45E-14 | 8.3E-13 |
| ENSECAG00000016149 | 10.48754 | 1.6E-14 | 9.18E-13 |
| ENSECAG00000006035 | 10.46716 | 1.69E-14 | 9.63E-13 |
| ENSECAG00000007095 | 10.45194 | 2.4E-14 | 1.36E-12 |
| ENSECAG00000023551 | 10.42771 | 2.67E-14 | 1.51E-12 |
| ENSECAG00000003207 | 10.41133 | 2.75E-14 | 1.55E-12 |
| ENSECAG00000006686 | 10.41544 | 2.91E-14 | 1.63E-12 |
| ENSECAG00000017484 | 10.3864 | 3.44E-14 | 1.91E-12 |
| ENSECAG00000009531 | 6.211036 | 8.32E-14 | 4.61E-12 |
| ENSECAG00000015296 | 10.22325 | 8.9E-14 | 4.92E-12 |
| ENSECAG00000005400 | 10.18911 | 1.04E-13 | 5.73E-12 |
| ENSECAG00000024406 | 10.21289 | 1.07E-13 | 5.89E-12 |
| ENSECAG00000014723 | 10.17463 | 1.22E-13 | 6.65E-12 |
| ENSECAG00000005916 | -10.1488 | 1.39E-13 | 7.54E-12 |
| ENSECAG00000003000 | -10.0547 | 1.69E-13 | 9.15E-12 |
| ENSECAG00000012092 | 6.209201 | 1.9E-13 | 1.02E-11 |
| ENSECAG00000020242 | -10.0824 | 2.38E-13 | 1.27E-11 |
| ENSECAG00000004709 | 10.06893 | 2.73E-13 | 1.45E-11 |
| ENSECAG00000012744 | 10.07414 | 2.73E-13 | 1.45E-11 |
| ENSECAG00000007502 | 5.852909 | 3.46E-13 | 1.84E-11 |
| ENSECAG00000003973 | -5.92415 | 3.95E-13 | 2.09E-11 |
| ENSECAG00000018367 | 9.999387 | 4.04E-13 | 2.13E-11 |
| ENSECAG00000017365 | 5.916343 | 4.19E-13 | 2.2E-11 |
| ENSECAG00000010402 | 9.971722 | 5.04E-13 | 2.63E-11 |
| ENSECAG00000023441 | 9.960506 | 5.43E-13 | 2.82E-11 |
| ENSECAG00000005396 | -9.85748 | 5.64E-13 | 2.92E-11 |
| ENSECAG00000020774 | -9.88287 | 8.61E-13 | 4.42E-11 |
| ENSECAG00000015381 | 9.885396 | 8.95E-13 | 4.58E-11 |
| ENSECAG00000000760 | -5.84798 | 1.07E-12 | 5.44E-11 |
| ENSECAG00000020325 | -9.70208 | 1.23E-12 | 6.24E-11 |
| ENSECAG00000002892 | 9.80616 | 1.34E-12 | 6.75E-11 |
| ENSECAG00000023659 | 9.818637 | 1.39E-12 | 7.01E-11 |
| ENSECAG00000013414 | 9.80616 | 1.65E-12 | 8.25E-11 |
| ENSECAG00000023397 | 9.654336 | 4.14E-12 | 2.07E-10 |
| ENSECAG00000014263 | 9.640347 | 4.54E-12 | 2.26E-10 |
| ENSECAG00000024526 | -9.59375 | 4.76E-12 | 2.35E-10 |
| ENSECAG00000010666 | 9.633301 | 4.76E-12 | 2.35E-10 |
| ENSECAG00000018577 | 9.590287 | 5.23E-12 | 2.57E-10 |
| ENSECAG00000000728 | 9.619105 | 5.23E-12 | 2.57E-10 |
| ENSECAG00000003315 | -5.42343 | 5.97E-12 | 2.92E-10 |
| ENSECAG00000006358 | -9.61384 | 6.64E-12 | 3.22E-10 |
| ENSECAG00000019789 | 9.56829 | 6.64E-12 | 3.22E-10 |
| ENSECAG00000014995 | 9.568363 | 7.31E-12 | 3.54E-10 |
| ENSECAG00000004499 | -5.48283 | 7.71E-12 | 3.72E-10 |
| ENSECAG00000004119 | 9.523262 | 9.36E-12 | 4.5E-10 |
| ENSECAG00000003022 | -5.48896 | 1.11E-11 | 5.29E-10 |
| ENSECAG00000014202 | 5.351961 | 1.11E-11 | 5.29E-10 |
| ENSECAG00000014719 | 5.353003 | 1.1E-11 | 5.29E-10 |
| ENSECAG00000002309 | -5.48581 | 1.14E-11 | 5.42E-10 |
| ENSECAG00000001053 | -5.46382 | 1.32E-11 | 6.26E-10 |
| ENSECAG00000015041 | 9.46889 | 1.34E-11 | 6.3E-10 |
| ENSECAG00000005164 | -5.45491 | 1.38E-11 | 6.5E-10 |
| ENSECAG00000004347 | 5.203523 | 2E-11 | 9.35E-10 |
| ENSECAG00000016936 | -5.10106 | 2.02E-11 | 9.44E-10 |
| ENSECAG00000008801 | -5.37093 | 2.41E-11 | 1.12E-09 |
| ENSECAG00000002642 | -5.36804 | 2.47E-11 | 1.15E-09 |
| ENSECAG00000017411 | -5.37162 | 2.54E-11 | 1.18E-09 |
| ENSECAG00000016876 | 5.190308 | 2.66E-11 | 1.23E-09 |
| ENSECAG00000015859 | 9.31006 | 3.56E-11 | 1.64E-09 |
| ENSECAG00000007474 | -5.04499 | 4.18E-11 | 1.91E-09 |
| ENSECAG00000012626 | -5.18773 | 5.05E-11 | 2.31E-09 |
| ENSECAG00000023983 | -9.23436 | 5.69E-11 | 2.59E-09 |
| ENSECAG00000021522 | -9.1636 | 8.22E-11 | 3.71E-09 |
| ENSECAG00000020890 | 9.161252 | 8.22E-11 | 3.71E-09 |
| ENSECAG00000004993 | 9.171016 | 8.22E-11 | 3.71E-09 |
| ENSECAG00000024543 | 5.151317 | 8.7E-11 | 3.91E-09 |
| ENSECAG00000024479 | 9.180714 | 8.75E-11 | 3.92E-09 |
| ENSECAG00000022578 | 9.151421 | 9.32E-11 | 4.17E-09 |
| ENSECAG00000003839 | -5.16155 | 9.4E-11 | 4.19E-09 |
| ENSECAG00000015630 | 9.151421 | 9.93E-11 | 4.41E-09 |
| ENSECAG00000019241 | -9.05608 | 1.06E-10 | 4.69E-09 |
| ENSECAG00000006922 | -5.66556 | 1.21E-10 | 5.36E-09 |
| ENSECAG00000017174 | 5.230491 | 1.35E-10 | 5.96E-09 |
| ENSECAG00000011232 | -9.0672 | 1.37E-10 | 6.02E-09 |
| ENSECAG00000020209 | -5.04993 | 1.91E-10 | 8.35E-09 |
| ENSECAG00000019472 | 9.025117 | 1.91E-10 | 8.35E-09 |
| ENSECAG00000014048 | 9.027913 | 2.05E-10 | 8.91E-09 |
| ENSECAG00000012290 | 5.137674 | 2.11E-10 | 9.15E-09 |
| ENSECAG00000014332 | 8.99531 | 2.52E-10 | 1.09E-08 |
| ENSECAG00000003107 | -5.00029 | 2.64E-10 | 1.14E-08 |
| ENSECAG00000017624 | 8.950661 | 3.12E-10 | 1.34E-08 |
| ENSECAG00000001564 | -4.94174 | 3.79E-10 | 1.62E-08 |
| ENSECAG00000000222 | -4.94546 | 3.86E-10 | 1.65E-08 |
| ENSECAG00000003678 | -4.89135 | 5.25E-10 | 2.23E-08 |
| ENSECAG00000003686 | -4.89119 | 5.25E-10 | 2.23E-08 |
| ENSECAG00000001662 | -4.84745 | 6.87E-10 | 2.91E-08 |
| ENSECAG00000002068 | -4.84697 | 6.94E-10 | 2.93E-08 |
| ENSECAG00000012243 | -4.84308 | 7.06E-10 | 2.97E-08 |
| ENSECAG00000009398 | -8.78688 | 7.63E-10 | 3.2E-08 |
| ENSECAG00000008766 | -4.58687 | 9.67E-10 | 4.05E-08 |
| ENSECAG00000015241 | 4.736204 | 1.07E-09 | 4.47E-08 |
| ENSECAG00000016941 | -4.72203 | 1.5E-09 | 6.26E-08 |
| ENSECAG00000015756 | -4.69789 | 1.72E-09 | 7.15E-08 |
| ENSECAG00000003096 | -4.85002 | 1.86E-09 | 7.72E-08 |
| ENSECAG00000004559 | -4.67179 | 2.1E-09 | 8.66E-08 |
| ENSECAG00000016967 | 4.528965 | 2.25E-09 | 9.26E-08 |
| ENSECAG00000020806 | -4.32931 | 2.99E-09 | 1.23E-07 |
| ENSECAG00000022012 | 4.524319 | 3.51E-09 | 1.44E-07 |
| ENSECAG00000022316 | -4.57574 | 3.77E-09 | 1.54E-07 |
| ENSECAG00000022614 | 4.579722 | 3.96E-09 | 1.61E-07 |
| ENSECAG00000004138 | -4.57325 | 3.99E-09 | 1.62E-07 |
| ENSECAG00000005614 | 4.6637 | 4.12E-09 | 1.67E-07 |
| ENSECAG00000018765 | -4.38126 | 4.77E-09 | 1.92E-07 |
| ENSECAG00000003477 | -4.51713 | 5.61E-09 | 2.26E-07 |
| ENSECAG00000009257 | -4.6334 | 5.69E-09 | 2.28E-07 |
| ENSECAG00000001621 | 4.393469 | 5.83E-09 | 2.33E-07 |
| ENSECAG00000002532 | -4.50551 | 6.07E-09 | 2.42E-07 |
| ENSECAG00000005333 | -4.48361 | 6.79E-09 | 2.7E-07 |
| ENSECAG00000006391 | 4.539431 | 7.24E-09 | 2.87E-07 |
| ENSECAG00000010578 | 4.408706 | 7.48E-09 | 2.96E-07 |
| ENSECAG00000013568 | -4.44056 | 8.85E-09 | 3.5E-07 |
| ENSECAG00000001542 | 4.2592 | 9.03E-09 | 3.56E-07 |
| ENSECAG00000023198 | -4.44016 | 9.13E-09 | 3.58E-07 |
| ENSECAG00000013000 | 4.352772 | 9.55E-09 | 3.74E-07 |
| ENSECAG00000012001 | 4.409225 | 1.05E-08 | 4.12E-07 |
| ENSECAG00000007340 | -4.24364 | 2.1E-08 | 8.17E-07 |
| ENSECAG00000004923 | 4.173867 | 2.19E-08 | 8.52E-07 |
| ENSECAG00000001760 | -4.24417 | 2.92E-08 | 1.13E-06 |
| ENSECAG00000003340 | -4.16981 | 2.98E-08 | 1.15E-06 |
| ENSECAG00000004622 | -4.19615 | 4.05E-08 | 1.56E-06 |
| ENSECAG00000001696 | -4.17604 | 4.54E-08 | 1.74E-06 |
| ENSECAG00000011936 | 4.112997 | 4.91E-08 | 1.87E-06 |
| ENSECAG00000011957 | 4.112997 | 4.91E-08 | 1.87E-06 |
| ENSECAG00000015364 | 4.112997 | 4.91E-08 | 1.87E-06 |
| ENSECAG00000006122 | -3.97631 | 5.97E-08 | 2.27E-06 |
| ENSECAG00000022417 | -4.09343 | 6.76E-08 | 2.56E-06 |
| ENSECAG00000005102 | 4.351574 | 8.97E-08 | 3.38E-06 |
| ENSECAG00000011183 | 3.989017 | 9.27E-08 | 3.48E-06 |
| ENSECAG00000005009 | 4.016811 | 1.14E-07 | 4.26E-06 |
| ENSECAG00000025025 | -4.14156 | 1.44E-07 | 5.39E-06 |
| ENSECAG00000003453 | -3.66788 | 2E-07 | 7.46E-06 |
| ENSECAG00000011803 | 3.788083 | 2.06E-07 | 7.67E-06 |
| ENSECAG00000003065 | 3.864006 | 2.11E-07 | 7.82E-06 |
| ENSECAG00000015004 | -3.93722 | 2.93E-07 | 1.08E-05 |
| ENSECAG00000015601 | 3.71152 | 2.91E-07 | 1.08E-05 |
| ENSECAG00000022755 | -3.74659 | 3.14E-07 | 1.16E-05 |
| ENSECAG00000020951 | -3.81385 | 3.98E-07 | 1.46E-05 |
| ENSECAG00000008587 | 3.852942 | 3.99E-07 | 1.46E-05 |
| ENSECAG00000004508 | 3.732941 | 4.04E-07 | 1.48E-05 |
| ENSECAG00000012717 | -3.77542 | 4.32E-07 | 1.57E-05 |
| ENSECAG00000005687 | -3.77016 | 5.15E-07 | 1.87E-05 |
| ENSECAG00000013316 | 3.589856 | 5.14E-07 | 1.87E-05 |
| ENSECAG00000008886 | 3.711083 | 5.51E-07 | 1.99E-05 |
| ENSECAG00000002667 | -3.73797 | 6.53E-07 | 2.35E-05 |
| ENSECAG00000008925 | -3.70347 | 7.78E-07 | 0.000028 |
| ENSECAG00000002884 | 3.518817 | 8.82E-07 | 3.16E-05 |
| ENSECAG00000001837 | -3.67644 | 9.23E-07 | 0.000033 |
| ENSECAG00000014212 | 3.584504 | 1.05E-06 | 3.76E-05 |
| ENSECAG00000005089 | -3.63799 | 1.12E-06 | 3.96E-05 |
| ENSECAG00000019550 | 3.59586 | 1.11E-06 | 3.96E-05 |
| ENSECAG00000023402 | -3.6324 | 1.19E-06 | 4.23E-05 |
| ENSECAG00000024400 | -3.63459 | 1.22E-06 | 4.32E-05 |
| ENSECAG00000018047 | -3.59334 | 1.24E-06 | 4.37E-05 |
| ENSECAG00000017053 | 3.452871 | 1.24E-06 | 4.37E-05 |
| ENSECAG00000002718 | -3.61566 | 1.36E-06 | 4.78E-05 |
| ENSECAG00000011300 | -3.57401 | 1.39E-06 | 4.88E-05 |
| ENSECAG00000001992 | -3.47373 | 1.41E-06 | 4.93E-05 |
| ENSECAG00000006152 | -3.57703 | 1.55E-06 | 0.000054 |
| ENSECAG00000015086 | -3.39212 | 1.76E-06 | 6.13E-05 |
| ENSECAG00000022825 | -3.52428 | 2.19E-06 | 0.000076 |
| ENSECAG00000003198 | 3.412557 | 2.21E-06 | 7.64E-05 |
| ENSECAG00000005955 | -3.44739 | 2.29E-06 | 7.92E-05 |
| ENSECAG00000007076 | -3.51404 | 2.37E-06 | 8.16E-05 |
| ENSECAG00000002004 | 3.380427 | 2.77E-06 | 9.48E-05 |
| ENSECAG00000005364 | -3.48373 | 2.8E-06 | 9.58E-05 |
| ENSECAG00000003490 | -3.49598 | 3.16E-06 | 0.000108 |
| ENSECAG00000016311 | 3.356738 | 3.18E-06 | 0.000108 |
| ENSECAG00000001711 | -3.46684 | 3.21E-06 | 0.000109 |
| ENSECAG00000004193 | -3.43613 | 3.21E-06 | 0.000109 |
| ENSECAG00000002628 | -3.45173 | 3.29E-06 | 0.000111 |
| ENSECAG00000005429 | 3.334195 | 3.58E-06 | 0.000121 |
| ENSECAG00000020773 | -3.44175 | 3.97E-06 | 0.000134 |
| ENSECAG00000013705 | -3.39642 | 4.05E-06 | 0.000136 |
| ENSECAG00000003197 | -3.41606 | 4.08E-06 | 0.000137 |
| ENSECAG00000003286 | -3.40438 | 4.4E-06 | 0.000147 |
| ENSECAG00000005986 | -3.37062 | 4.51E-06 | 0.00015 |
| ENSECAG00000010096 | 3.216865 | 4.58E-06 | 0.000152 |
| ENSECAG00000002103 | -3.38892 | 4.72E-06 | 0.000156 |
| ENSECAG00000016944 | -3.35208 | 4.89E-06 | 0.000161 |
| ENSECAG00000024800 | -3.34876 | 5.1E-06 | 0.000168 |
| ENSECAG00000018556 | -3.3792 | 5.43E-06 | 0.000178 |
| ENSECAG00000005233 | -3.35496 | 5.76E-06 | 0.000188 |
| ENSECAG00000002119 | -3.28181 | 5.8E-06 | 0.000189 |
| ENSECAG00000023974 | -3.23025 | 6.02E-06 | 0.000196 |
| ENSECAG00000003764 | -3.32925 | 6.88E-06 | 0.000222 |
| ENSECAG00000014761 | -3.2097 | 7.02E-06 | 0.000227 |
| ENSECAG00000022657 | 3.171466 | 7.57E-06 | 0.000243 |
| ENSECAG00000023898 | 3.234936 | 7.89E-06 | 0.000253 |
| ENSECAG00000010277 | 3.059001 | 7.97E-06 | 0.000255 |
| ENSECAG00000004577 | -3.29684 | 8.08E-06 | 0.000258 |
| ENSECAG00000013665 | -3.26707 | 8.16E-06 | 0.00026 |
| ENSECAG00000022091 | -3.21463 | 9.32E-06 | 0.000296 |
| ENSECAG00000022457 | 3.129438 | 9.66E-06 | 0.000306 |
| ENSECAG00000000196 | -3.05282 | 1.08E-05 | 0.000343 |
| ENSECAG00000011712 | 3.117024 | 1.11E-05 | 0.000351 |
| ENSECAG00000000510 | -3.1726 | 1.14E-05 | 0.00036 |
| ENSECAG00000009601 | -3.22486 | 1.16E-05 | 0.000366 |
| ENSECAG00000009310 | -3.25159 | 1.23E-05 | 0.000387 |
| ENSECAG00000002191 | -3.21827 | 1.24E-05 | 0.000388 |
| ENSECAG00000004038 | 3.097635 | 0.000013 | 0.000407 |
| ENSECAG00000011161 | 3.046887 | 1.31E-05 | 0.000408 |
| ENSECAG00000002151 | -3.19999 | 1.36E-05 | 0.000423 |
| ENSECAG00000018940 | -3.13034 | 1.45E-05 | 0.000451 |
| ENSECAG00000001803 | -3.18534 | 1.47E-05 | 0.000454 |
| ENSECAG00000007312 | 3.087698 | 1.47E-05 | 0.000455 |
| ENSECAG00000014698 | -3.08009 | 0.000016 | 0.000494 |
| ENSECAG00000002540 | 3.182462 | 1.61E-05 | 0.000496 |
| ENSECAG00000020524 | -3.10351 | 1.62E-05 | 0.000496 |
| ENSECAG00000001543 | -3.06384 | 1.65E-05 | 0.000505 |
| ENSECAG00000017937 | 3.286507 | 1.76E-05 | 0.000539 |
| ENSECAG00000016970 | 3.001772 | 1.83E-05 | 0.000559 |
| ENSECAG00000006254 | 3.209826 | 1.91E-05 | 0.00058 |
| ENSECAG00000022769 | 2.9251 | 1.92E-05 | 0.000584 |
| ENSECAG00000024507 | -3.16922 | 1.97E-05 | 0.000597 |
| ENSECAG00000024650 | -2.89508 | 2.05E-05 | 0.00062 |
| ENSECAG00000015297 | -3.1177 | 0.000021 | 0.000635 |
| ENSECAG00000009597 | 2.964686 | 2.14E-05 | 0.000644 |
| ENSECAG00000012065 | -3.04873 | 2.21E-05 | 0.000664 |
| ENSECAG00000003059 | -3.11229 | 2.24E-05 | 0.000674 |
| ENSECAG00000018372 | -3.0962 | 2.36E-05 | 0.000706 |
| ENSECAG00000005553 | -2.94684 | 0.000024 | 0.000717 |
| ENSECAG00000002764 | 3.151059 | 2.51E-05 | 0.000747 |
| ENSECAG00000020570 | 2.893145 | 2.53E-05 | 0.000751 |
| ENSECAG00000013351 | -3.05125 | 2.59E-05 | 0.000767 |
| ENSECAG00000016255 | 2.875226 | 0.000028 | 0.000828 |
| ENSECAG00000003709 | 2.857172 | 3.05E-05 | 0.000901 |
| ENSECAG00000020941 | -3.01267 | 3.08E-05 | 0.000909 |
| ENSECAG00000015027 | -3.00176 | 0.000031 | 0.000911 |
| ENSECAG00000021635 | 2.890229 | 3.18E-05 | 0.000933 |
| ENSECAG00000019723 | -3.05353 | 3.24E-05 | 0.000948 |
| ENSECAG00000006221 | -3.00417 | 3.26E-05 | 0.000953 |
| ENSECAG00000014233 | -3.04007 | 3.37E-05 | 0.000981 |
| ENSECAG00000021217 | 3.223498 | 3.37E-05 | 0.000981 |
| ENSECAG00000008804 | -3.02868 | 3.48E-05 | 0.00101 |
| ENSECAG00000003755 | 3.136578 | 3.62E-05 | 0.00105 |
| ENSECAG00000001793 | 3.106545 | 3.87E-05 | 0.001118 |
| ENSECAG00000001838 | -3.00129 | 0.00004 | 0.001153 |
| ENSECAG00000023651 | 2.861757 | 4.05E-05 | 0.001165 |
| ENSECAG00000023848 | -3.02945 | 4.14E-05 | 0.00119 |
| ENSECAG00000008018 | 2.868052 | 0.000043 | 0.001233 |
| ENSECAG00000004140 | -2.95415 | 4.46E-05 | 0.001275 |
| ENSECAG00000022360 | -2.96235 | 4.71E-05 | 0.001343 |
| ENSECAG00000015279 | -2.81129 | 4.71E-05 | 0.001343 |
| ENSECAG00000011058 | -2.91179 | 4.91E-05 | 0.001395 |
| ENSECAG00000021878 | 2.842074 | 4.92E-05 | 0.001395 |
| ENSECAG00000011026 | 3.057705 | 4.95E-05 | 0.001401 |
| ENSECAG00000019754 | -2.78188 | 4.96E-05 | 0.001402 |
| ENSECAG00000009060 | 2.987841 | 5.32E-05 | 0.001501 |
| ENSECAG00000024153 | -2.84791 | 5.69E-05 | 0.001603 |
| ENSECAG00000023649 | -2.91836 | 6.06E-05 | 0.001702 |
| ENSECAG00000011802 | -2.89631 | 6.08E-05 | 0.001705 |
| ENSECAG00000012447 | 2.845443 | 6.23E-05 | 0.001743 |
| ENSECAG00000002233 | 2.903358 | 0.000064 | 0.001789 |
| ENSECAG00000005946 | -2.82554 | 6.59E-05 | 0.001838 |
| ENSECAG00000019129 | -2.66297 | 0.000066 | 0.001838 |
| ENSECAG00000007280 | -2.69923 | 6.72E-05 | 0.001867 |
| ENSECAG00000022053 | -2.81716 | 6.95E-05 | 0.001928 |
| ENSECAG00000002685 | -2.84391 | 0.00007 | 0.001938 |
| ENSECAG00000021539 | -2.7352 | 7.12E-05 | 0.001967 |
| ENSECAG00000018513 | 2.880607 | 7.19E-05 | 0.001983 |
| ENSECAG00000005284 | -2.84117 | 7.34E-05 | 0.002019 |
| ENSECAG00000002360 | 2.84303 | 7.77E-05 | 0.002133 |
| ENSECAG00000001744 | 2.734851 | 8.06E-05 | 0.00221 |
| ENSECAG00000012046 | -2.73804 | 8.14E-05 | 0.002227 |
| ENSECAG00000019537 | 2.798827 | 8.55E-05 | 0.002333 |
| ENSECAG00000018936 | 2.71356 | 8.81E-05 | 0.002402 |
| ENSECAG00000018389 | -2.88133 | 8.92E-05 | 0.002426 |
| ENSECAG00000008391 | 2.688509 | 9.36E-05 | 0.002543 |
| ENSECAG00000020926 | -2.70613 | 9.59E-05 | 0.002599 |
| ENSECAG00000004769 | -2.68161 | 0.0001 | 0.002711 |
| ENSECAG00000012201 | -2.66445 | 0.000103 | 0.002773 |
| ENSECAG00000020580 | -2.76992 | 0.000108 | 0.002905 |
| ENSECAG00000022947 | 2.615599 | 0.000113 | 0.003046 |
| ENSECAG00000009374 | -2.78523 | 0.000124 | 0.003323 |
| ENSECAG00000020542 | -2.66835 | 0.000125 | 0.003334 |
| ENSECAG00000004829 | 2.604484 | 0.000132 | 0.003527 |
| ENSECAG00000000628 | -2.72785 | 0.000133 | 0.00354 |
| ENSECAG00000008124 | -2.46207 | 0.00014 | 0.00372 |
| ENSECAG00000003878 | 2.629846 | 0.000148 | 0.003921 |
| ENSECAG00000007306 | -2.70647 | 0.000148 | 0.003924 |
| ENSECAG00000018602 | 2.655172 | 0.000161 | 0.004256 |
| ENSECAG00000008779 | -2.69094 | 0.000162 | 0.004277 |
| ENSECAG00000009938 | 2.724819 | 0.000163 | 0.004285 |
| ENSECAG00000012195 | 2.611085 | 0.000168 | 0.004403 |
| ENSECAG00000005920 | 2.580949 | 0.000169 | 0.00444 |
| ENSECAG00000025176 | 2.795258 | 0.000184 | 0.004805 |
| ENSECAG00000017472 | -2.80008 | 0.000187 | 0.004865 |
| ENSECAG00000002194 | 2.581006 | 0.000189 | 0.004921 |
| ENSECAG00000000741 | -2.63075 | 0.000212 | 0.005499 |
| ENSECAG00000004070 | -2.61491 | 0.000214 | 0.005554 |
| ENSECAG00000004026 | -2.58839 | 0.000217 | 0.005604 |
| ENSECAG00000008198 | 2.494534 | 0.00022 | 0.00568 |
| ENSECAG00000005462 | -2.55215 | 0.000234 | 0.006024 |
| ENSECAG00000014955 | 2.612684 | 0.000235 | 0.006038 |
| ENSECAG00000002114 | 2.516199 | 0.000238 | 0.006111 |
| ENSECAG00000014013 | -2.63389 | 0.000245 | 0.006269 |
| ENSECAG00000006322 | 2.582251 | 0.000245 | 0.00627 |
| ENSECAG00000023646 | -2.62196 | 0.000248 | 0.006336 |
| ENSECAG00000001460 | 2.627781 | 0.000249 | 0.00634 |
| ENSECAG00000006167 | -2.63861 | 0.000253 | 0.006435 |
| ENSECAG00000009397 | -2.60385 | 0.000259 | 0.006573 |
| ENSECAG00000006551 | 2.538527 | 0.000259 | 0.006579 |
| ENSECAG00000013329 | 2.523268 | 0.000265 | 0.006702 |
| ENSECAG00000012740 | 2.544962 | 0.000271 | 0.006855 |
| ENSECAG00000002965 | -2.62286 | 0.000273 | 0.006882 |
| ENSECAG00000008230 | 2.552724 | 0.000275 | 0.00691 |
| ENSECAG00000016723 | -2.59293 | 0.000277 | 0.006945 |
| ENSECAG00000016309 | -2.5381 | 0.000278 | 0.006959 |
| ENSECAG00000006489 | 2.449093 | 0.000285 | 0.007122 |
| ENSECAG00000010101 | -2.58103 | 0.000288 | 0.007191 |
| ENSECAG00000007930 | -2.62139 | 0.000295 | 0.007339 |
| ENSECAG00000019846 | -2.51815 | 0.000299 | 0.007428 |
| ENSECAG00000019142 | 2.507084 | 0.000306 | 0.007593 |
| ENSECAG00000018200 | 2.551938 | 0.000311 | 0.007703 |
| ENSECAG00000001957 | -2.66453 | 0.00033 | 0.008161 |
| ENSECAG00000003683 | -2.56059 | 0.000353 | 0.00872 |
| ENSECAG00000008449 | -2.54627 | 0.000355 | 0.008757 |
| ENSECAG00000010570 | 2.865059 | 0.000363 | 0.008934 |
| ENSECAG00000016618 | 2.664433 | 0.000364 | 0.008939 |
| ENSECAG00000000454 | 2.571661 | 0.000387 | 0.009497 |
| ENSECAG00000012996 | -2.49201 | 0.00039 | 0.009563 |
| ENSECAG00000014206 | -2.52121 | 0.0004 | 0.009772 |
| ENSECAG00000000660 | -2.54337 | 0.000402 | 0.009827 |

**Table S2.** the number and rate of SNPs from different next-generation sequencing method (DNA and RNA sequencing) and different reference genome assembly in each Thoroughbred horse sample (F1, F2 and F3 = male, S3 = female)

| SNP detection | | F1 | F2 | F3 | S3 |
| --- | --- | --- | --- | --- | --- |
| Sequence | Reference |  |  |  |  |
| WGS | DNA | 3,797,464  (0.153%) | 3,716,018  (0.150%) | 3,759,757  (0.152%) | 3,628,882  (0.147%) |
|  | cDNA | 35,591  (0.08%) | 38,275  (0.082%) | 36,058  (0.077%) | 36,121  (0.078%) |
| RNA-seq | DNA | 284,859  (0.012%) | 287,286  (0.012%) | 276,241  (0.011%) | 265,729  (0.011) |
|  | cDNA | 29,507  (0.063%) | 30,570  (0.066%) | 30,145  (0.065%) | 29,518  (0.063%) |
| RNA-seq | Trinity(*de novo*) | 108,158  (0.031%) | 110,502  (0.031%) | 105,920  (0.030%) | 101,887  (0.029%) |

**Table S3** GO terms of cellular components and molecular function of two tissues specific DEGs between before the exercise and after exercise in horses

1. **Up-regulated DEGs**

| **Tissue** | **Categories** | **GO ID** | **Term** | **Count** | **P-value** |
| --- | --- | --- | --- | --- | --- |
| Blood | Molecular Function | GO:0030554 | adenyl nucleotide binding | 14 | 4.666E-02 |
|  |  | GO:0032559 | adenyl ribonucleotide binding | 14 | 3.248E-02 |
|  |  | GO:0005524 | ATP binding | 14 | 2.950E-02 |
|  |  | GO:0016887 | ATPase activity | 5 | 8.603E-02 |
|  |  | GO:0042623 | ATPase activity, coupled | 5 | 4.720E-02 |
|  |  | GO:0003677 | DNA binding | 18 | 6.359E-02 |
|  |  | GO:0008047 | enzyme activator activity | 6 | 2.598E-02 |
|  |  | GO:0005096 | GTPase activator activity | 4 | 9.785E-02 |
|  |  | GO:0051427 | hormone receptor binding | 3 | 7.181E-02 |
|  |  | GO:0005506 | iron ion binding | 5 | 6.825E-02 |
|  |  | GO:0004715 | non-membrane spanning protein tyrosine kinase activity | 3 | 1.785E-02 |
|  |  | GO:0035257 | nuclear hormone receptor binding | 3 | 5.682E-02 |
|  |  | GO:0001882 | nucleoside binding | 14 | 5.408E-02 |
|  |  | GO:0000166 | nucleotide binding | 20 | 1.188E-02 |
|  |  | GO:0016717 | oxidoreductase activity, acting on paired donors, with oxidation of a pair of donors resulting in the reduction of molecular oxygen to two molecules of water | 3 | 3.655E-04 |
|  |  | GO:0010843 | promoter binding | 3 | 3.302E-02 |
|  |  | GO:0046983 | protein dimerization activity | 7 | 5.341E-02 |
|  |  | GO:0001883 | purine nucleoside binding | 14 | 5.166E-02 |
|  |  | GO:0017076 | purine nucleotide binding | 16 | 4.899E-02 |
|  |  | GO:0032555 | purine ribonucleotide binding | 16 | 3.512E-02 |
|  |  | GO:0032553 | ribonucleotide binding | 16 | 3.512E-02 |
|  |  | GO:0003723 | RNA binding | 11 | 2.841E-03 |
|  |  | GO:0043565 | sequence-specific DNA binding | 8 | 3.148E-02 |
|  |  | GO:0004768 | stearoyl-CoA 9-desaturase activity | 2 | 1.495E-02 |
|  | Cellular Component | GO:0005783 | endoplasmic reticulum | 15 | 3.199E-04 |
|  |  | GO:0005788 | endoplasmic reticulum lumen | 3 | 6.417E-02 |
|  |  | GO:0005794 | Golgi apparatus | 10 | 3.437E-02 |
|  |  | GO:0070013 | intracellular organelle lumen | 21 | 4.498E-04 |
|  |  | GO:0031974 | membrane-enclosed lumen | 21 | 7.824E-04 |
|  |  | GO:0031981 | nuclear lumen | 17 | 2.384E-03 |
|  |  | GO:0016607 | nuclear speck | 3 | 9.903E-02 |
|  |  | GO:0005730 | nucleolus | 10 | 9.315E-03 |
|  |  | GO:0005654 | nucleoplasm | 9 | 8.314E-02 |
|  |  | GO:0044451 | nucleoplasm part | 7 | 6.614E-02 |
|  |  | GO:0043233 | organelle lumen | 21 | 6.068E-04 |
| Muscle | Molecular Function | GO:0030554 | adenyl nucleotide binding | 47 | 2.629E-02 |
|  |  | GO:0032559 | adenyl ribonucleotide binding | 44 | 3.900E-02 |
|  |  | GO:0005524 | ATP binding | 43 | 4.712E-02 |
|  |  | GO:0005516 | calmodulin binding | 8 | 3.475E-02 |
|  |  | GO:0030246 | carbohydrate binding | 16 | 1.202E-02 |
|  |  | GO:0019956 | chemokine binding | 4 | 2.075E-02 |
|  |  | GO:0005125 | cytokine activity | 12 | 3.917E-03 |
|  |  | GO:0019955 | cytokine binding | 15 | 1.087E-07 |
|  |  | GO:0004896 | cytokine receptor activity | 10 | 2.738E-06 |
|  |  | GO:0008047 | enzyme activator activity | 17 | 3.013E-03 |
|  |  | GO:0019899 | enzyme binding | 21 | 1.151E-02 |
|  |  | GO:0004857 | enzyme inhibitor activity | 12 | 3.666E-02 |
|  |  | GO:0043499 | eukaryotic cell surface binding | 3 | 5.258E-02 |
|  |  | GO:0004370 | glycerol kinase activity | 2 | 6.443E-02 |
|  |  | GO:0001948 | glycoprotein binding | 4 | 4.701E-02 |
|  |  | GO:0005539 | glycosaminoglycan binding | 10 | 3.732E-03 |
|  |  | GO:0043208 | glycosphingolipid binding | 2 | 8.497E-02 |
|  |  | GO:0008083 | growth factor activity | 9 | 2.573E-02 |
|  |  | GO:0019838 | growth factor binding | 9 | 2.166E-03 |
|  |  | GO:0005096 | GTPase activator activity | 13 | 3.458E-03 |
|  |  | GO:0030695 | GTPase regulator activity | 16 | 3.318E-02 |
|  |  | GO:0008201 | heparin binding | 7 | 2.584E-02 |
|  |  | GO:0005149 | interleukin-1 receptor binding | 3 | 4.706E-02 |
|  |  | GO:0019979 | interleukin-4 binding | 2 | 4.342E-02 |
|  |  | GO:0004913 | interleukin-4 receptor activity | 2 | 4.342E-02 |
|  |  | GO:0019982 | interleukin-7 binding | 2 | 4.342E-02 |
|  |  | GO:0004917 | interleukin-7 receptor activity | 2 | 4.342E-02 |
|  |  | GO:0019210 | kinase inhibitor activity | 5 | 8.495E-03 |
|  |  | GO:0019207 | kinase regulator activity | 7 | 1.648E-02 |
|  |  | GO:0001530 | lipopolysaccharide binding | 5 | 9.794E-05 |
|  |  | GO:0001875 | lipopolysaccharide receptor activity | 3 | 4.596E-03 |
|  |  | GO:0008034 | lipoprotein binding | 4 | 4.087E-02 |
|  |  | GO:0030169 | low-density lipoprotein binding | 3 | 7.671E-02 |
|  |  | GO:0000287 | magnesium ion binding | 18 | 2.188E-02 |
|  |  | GO:0001882 | nucleoside binding | 49 | 1.664E-02 |
|  |  | GO:0060589 | nucleoside-triphosphatase regulator activity | 16 | 3.911E-02 |
|  |  | GO:0001871 | pattern binding | 11 | 2.120E-03 |
|  |  | GO:0008329 | pattern recognition receptor activity | 4 | 3.184E-03 |
|  |  | GO:0030247 | polysaccharide binding | 11 | 2.120E-03 |
|  |  | GO:0032403 | protein complex binding | 9 | 6.825E-02 |
|  |  | GO:0046983 | protein dimerization activity | 25 | 8.808E-04 |
|  |  | GO:0042803 | protein homodimerization activity | 13 | 6.519E-02 |
|  |  | GO:0004672 | protein kinase activity | 22 | 2.615E-02 |
|  |  | GO:0004860 | protein kinase inhibitor activity | 5 | 7.707E-03 |
|  |  | GO:0019887 | protein kinase regulator activity | 7 | 8.708E-03 |
|  |  | GO:0004725 | protein tyrosine phosphatase activity | 6 | 7.877E-02 |
|  |  | GO:0001883 | purine nucleoside binding | 49 | 1.482E-02 |
|  |  | GO:0017076 | purine nucleotide binding | 53 | 5.929E-02 |
|  |  | GO:0032555 | purine ribonucleotide binding | 50 | 8.197E-02 |
|  |  | GO:0032553 | ribonucleotide binding | 50 | 8.197E-02 |
|  |  | GO:0043565 | sequence-specific DNA binding | 21 | 4.691E-02 |
|  |  | GO:0046332 | SMAD binding | 6 | 3.232E-03 |
|  |  | GO:0003700 | transcription factor activity | 30 | 5.794E-02 |
|  |  | GO:0008134 | transcription factor binding | 17 | 9.876E-02 |
|  | Cellular Component | GO:0000267 | cell fraction | 31 | 9.387E-02 |
|  |  | GO:0009986 | cell surface | 20 | 2.020E-04 |
|  |  | GO:0005829 | cytosol | 44 | 4.508E-03 |
|  |  | GO:0009897 | external side of plasma membrane | 13 | 3.203E-04 |
|  |  | GO:0005576 | extracellular region | 59 | 1.116E-02 |
|  |  | GO:0044421 | extracellular region part | 37 | 7.990E-04 |
|  |  | GO:0005615 | extracellular space | 30 | 4.070E-04 |
|  |  | GO:0005626 | insoluble fraction | 25 | 9.844E-02 |
|  |  | GO:0005887 | integral to plasma membrane | 55 | 9.406E-08 |
|  |  | GO:0031226 | intrinsic to plasma membrane | 55 | 1.963E-07 |
|  |  | GO:0005900 | oncostatin-M receptor complex | 2 | 6.339E-02 |
|  |  | GO:0005886 | plasma membrane | 114 | 2.970E-05 |
|  |  | GO:0044459 | plasma membrane part | 77 | 1.093E-05 |
|  |  | GO:0043235 | receptor complex | 12 | 4.120E-05 |

1. **Down-regulated DEGs**

| **Tissue** | **Categories** | **GO ID** | **Term** | **Count** | **P-value** |
| --- | --- | --- | --- | --- | --- |
| Blood | Molecular Function | GO:0004175 | endopeptidase activity | 7 | 2.953E-02 |
|  |  | GO:0008233 | peptidase activity | 9 | 2.641E-02 |
|  |  | GO:0070011 | peptidase activity, acting on L-amino acid peptides | 9 | 2.089E-02 |
|  |  | GO:0019787 | small conjugating protein ligase activity | 4 | 8.521E-02 |
|  |  | GO:0046914 | transition metal ion binding | 24 | 8.506E-02 |
|  |  | GO:0008270 | zinc ion binding | 22 | 4.283E-02 |
|  | Cellular Component | GO:0005814 | centriole | 3 | 1.241E-02 |
|  |  | GO:0012505 | endomembrane system | 10 | 2.623E-02 |
|  |  | GO:0005783 | endoplasmic reticulum | 10 | 7.756E-02 |
|  |  | GO:0005789 | endoplasmic reticulum membrane | 5 | 5.982E-02 |
|  |  | GO:0005794 | Golgi apparatus | 11 | 1.978E-02 |
|  |  | GO:0044431 | Golgi apparatus part | 7 | 5.377E-03 |
|  |  | GO:0000139 | Golgi membrane | 5 | 1.890E-02 |
|  |  | GO:0005871 | kinesin complex | 2 | 9.418E-02 |
|  |  | GO:0044450 | microtubule organizing center part | 3 | 3.766E-02 |
|  |  | GO:0042175 | nuclear envelope-endoplasmic reticulum network | 5 | 7.009E-02 |
|  |  | GO:0031090 | organelle membrane | 12 | 3.510E-02 |
|  |  | GO:0005625 | soluble fraction | 5 | 9.235E-02 |
|  |  | GO:0000151 | ubiquitin ligase complex | 3 | 8.708E-02 |
| Muscle | Molecular Function | GO:0030554 | adenyl nucleotide binding | 71 | 9.912E-03 |
|  |  | GO:0032559 | adenyl ribonucleotide binding | 68 | 9.585E-03 |
|  |  | GO:0015297 | antiporter activity | 6 | 7.971E-02 |
|  |  | GO:0005524 | ATP binding | 66 | 1.542E-02 |
|  |  | GO:0016887 | ATPase activity | 20 | 1.904E-02 |
|  |  | GO:0042623 | ATPase activity, coupled | 16 | 4.351E-02 |
|  |  | GO:0043492 | ATPase activity, coupled to movement of substances | 8 | 7.917E-02 |
|  |  | GO:0042626 | ATPase activity, coupled to transmembrane movement of substances | 8 | 7.622E-02 |
|  |  | GO:0043169 | cation binding | 177 | 2.354E-04 |
|  |  | GO:0015491 | cation:cation antiporter activity | 4 | 1.834E-02 |
|  |  | GO:0003677 | DNA binding | 102 | 3.811E-03 |
|  |  | GO:0003887 | DNA-directed DNA polymerase activity | 4 | 9.258E-02 |
|  |  | GO:0030695 | GTPase regulator activity | 22 | 3.460E-02 |
|  |  | GO:0005085 | guanyl-nucleotide exchange factor activity | 12 | 1.417E-02 |
|  |  | GO:0051864 | histone demethylase activity (H3-K36 specific) | 2 | 9.806E-02 |
|  |  | GO:0051879 | Hsp90 protein binding | 3 | 4.289E-02 |
|  |  | GO:0016820 | hydrolase activity, acting on acid anhydrides, catalyzing transmembrane movement of substances | 8 | 8.218E-02 |
|  |  | GO:0043167 | ion binding | 179 | 2.546E-04 |
|  |  | GO:0005319 | lipid transporter activity | 6 | 6.142E-02 |
|  |  | GO:0030145 | manganese ion binding | 11 | 3.632E-02 |
|  |  | GO:0046872 | metal ion binding | 174 | 4.270E-04 |
|  |  | GO:0008017 | microtubule binding | 6 | 9.621E-02 |
|  |  | GO:0001882 | nucleoside binding | 71 | 1.595E-02 |
|  |  | GO:0060589 | nucleoside-triphosphatase regulator activity | 22 | 4.222E-02 |
|  |  | GO:0000166 | nucleotide binding | 88 | 8.898E-02 |
|  |  | GO:0034593 | phosphatidylinositol bisphosphate phosphatase activity | 3 | 3.508E-02 |
|  |  | GO:0005548 | phospholipid transporter activity | 5 | 1.568E-02 |
|  |  | GO:0032947 | protein complex scaffold | 4 | 5.091E-02 |
|  |  | GO:0008022 | protein C-terminus binding | 11 | 2.139E-02 |
|  |  | GO:0004697 | protein kinase C activity | 3 | 8.964E-02 |
|  |  | GO:0001883 | purine nucleoside binding | 71 | 1.362E-02 |
|  |  | GO:0017076 | purine nucleotide binding | 79 | 4.399E-02 |
|  |  | GO:0032555 | purine ribonucleotide binding | 76 | 4.479E-02 |
|  |  | GO:0017112 | Rab guanyl-nucleotide exchange factor activity | 2 | 9.806E-02 |
|  |  | GO:0005088 | Ras guanyl-nucleotide exchange factor activity | 9 | 9.725E-03 |
|  |  | GO:0005089 | Rho guanyl-nucleotide exchange factor activity | 7 | 3.885E-02 |
|  |  | GO:0032553 | ribonucleotide binding | 76 | 4.479E-02 |
|  |  | GO:0046332 | SMAD binding | 5 | 6.914E-02 |
|  |  | GO:0005083 | small GTPase regulator activity | 17 | 2.412E-02 |
|  |  | GO:0015298 | solute:cation antiporter activity | 4 | 7.954E-02 |
|  |  | GO:0046914 | transition metal ion binding | 128 | 1.068E-04 |
|  |  | GO:0015631 | tubulin binding | 8 | 5.266E-02 |
|  |  | GO:0008270 | zinc ion binding | 107 | 3.944E-04 |
|  | Cellular Component | GO:0005680 | anaphase-promoting complex | 4 | 8.214E-03 |
|  |  | GO:0016323 | basolateral plasma membrane | 11 | 9.760E-02 |
|  |  | GO:0005938 | cell cortex | 9 | 8.221E-02 |
|  |  | GO:0031253 | cell projection membrane | 6 | 4.970E-02 |
|  |  | GO:0005813 | centrosome | 14 | 2.176E-02 |
|  |  | GO:0005712 | chiasma | 2 | 6.070E-02 |
|  |  | GO:0005694 | chromosome | 21 | 7.672E-02 |
|  |  | GO:0000793 | condensed chromosome | 9 | 4.611E-02 |
|  |  | GO:0000794 | condensed nuclear chromosome | 5 | 6.362E-02 |
|  |  | GO:0012505 | endomembrane system | 36 | 1.670E-02 |
|  |  | GO:0019898 | extrinsic to membrane | 23 | 5.383E-02 |
|  |  | GO:0005794 | Golgi apparatus | 49 | 6.111E-05 |
|  |  | GO:0044431 | Golgi apparatus part | 22 | 3.113E-04 |
|  |  | GO:0000139 | Golgi membrane | 18 | 6.259E-05 |
|  |  | GO:0005798 | Golgi-associated vesicle | 5 | 7.160E-02 |
|  |  | GO:0005779 | integral to peroxisomal membrane | 3 | 5.908E-02 |
|  |  | GO:0043232 | intracellular non-membrane-bounded organelle | 92 | 9.265E-02 |
|  |  | GO:0031231 | intrinsic to peroxisomal membrane | 3 | 5.908E-02 |
|  |  | GO:0043259 | laminin-10 complex | 2 | 8.966E-02 |
|  |  | GO:0005764 | lysosome | 13 | 3.050E-02 |
|  |  | GO:0000323 | lytic vacuole | 13 | 3.050E-02 |
|  |  | GO:0001673 | male germ cell nucleus | 3 | 9.509E-02 |
|  |  | GO:0031974 | membrane-enclosed lumen | 68 | 8.860E-02 |
|  |  | GO:0042579 | microbody | 8 | 3.984E-02 |
|  |  | GO:0031903 | microbody membrane | 4 | 6.918E-02 |
|  |  | GO:0005932 | microtubule basal body | 5 | 2.406E-02 |
|  |  | GO:0015630 | microtubule cytoskeleton | 25 | 5.410E-02 |
|  |  | GO:0005815 | microtubule organizing center | 16 | 1.189E-02 |
|  |  | GO:0044450 | microtubule organizing center part | 6 | 2.867E-02 |
|  |  | GO:0043228 | non-membrane-bounded organelle | 92 | 9.265E-02 |
|  |  | GO:0000152 | nuclear ubiquitin ligase complex | 4 | 1.681E-02 |
|  |  | GO:0043233 | organelle lumen | 68 | 6.610E-02 |
|  |  | GO:0031090 | organelle membrane | 45 | 4.179E-02 |
|  |  | GO:0005778 | peroxisomal membrane | 4 | 6.918E-02 |
|  |  | GO:0005777 | peroxisome | 8 | 3.984E-02 |
|  |  | GO:0005773 | vacuole | 15 | 2.418E-02 |

**Table S4.** Common genes between DEGs and selected genes associated with F_ST_ (F_ST_ cut-off value top 5% with empirical p-value < 0.05)

| **Sample**  **Tissue** | **Ens ID** | **CHR** | **Start** | **End** | **DEG**  **logFC** | **DEG**  **P-value** | **DEG**  **FDR** | **Gene**  **symbol** | **Reynolds** | **Fst** |
| --- | --- | --- | --- | --- | --- | --- | --- | --- | --- | --- |
| Blood | ENSECAG00000016283 | 1 | 92397477 | 92416004 | -14.5988 | 2.23E-26 | 5.06E-24 | ZNF592  (zinc finger protein 592) | 0.67059 | 0.48859 |
|  | ENSECAG00000024391 | 5 | 52474368 | 52488930 | -12.7633 | 6.63E-21 | 5.66E-19 | CD58  (CD58 molecule) | 0.63486 | 0.46999 |
|  | ENSECAG00000018585 | 5 | 56885158 | 56885977 | 13.73641 | 5.74E-24 | 7.51E-22 | C1orf162  (chromosome 1 open reading frame 162) | 0.66702 | 0.48676 |
|  | ENSECAG00000018200 | 6 | 8097875 | 8184891 | 2.551938 | 3.11E-04 | 7.70E-03 | USP37  (ubiquitin specific peptidase 37) | 0.68323 | 0.49502 |
|  | ENSECAG00000019129 | 6 | 30817902 | 30826537 | -2.66297 | 6.60E-05 | 1.84E-03 | FOXM1  (forkhead box M1) | 1.04819 | 0.64943 |
|  | ENSECAG00000002892 | 6 | 74016170 | 74027809 | 9.80616 | 1.34E-12 | 6.75E-11 | TIMELESS  (timeless circadian clock) | 0.67349 | 0.49007 |
|  | ENSECAG00000016149 | 7 | 45346016 | 45351774 | 10.48754 | 1.60E-14 | 9.18E-13 | TRMT1  (tRNA methyltransferase 1 homolog) | 1.11214 | 0.67114 |
|  | ENSECAG00000008164 | 7 | 45483000 | 45486353 | -16.153 | 4.48E-31 | 2.71E-28 | CALR  (calreticulin ) | 0.76759 | 0.53587 |
|  | ENSECAG00000007103 | 7 | 45610152 | 45615940 | 12.03488 | 6.08E-19 | 4.44E-17 | ASNA1  (arsA arsenite transporter, ATP-binding, homolog 1) | 0.82578 | 0.56211 |
|  | ENSECAG00000015925 | 11 | 2814986 | 2828951 | -17.23 | 1.81E-34 | 2.94E-31 | EIF4A3  (eukaryotic translation initiation factor 4A3) | 0.83481 | 0.56604 |
|  | ENSECAG00000008489 | 11 | 36666971 | 36750948 | 13.48276 | 3.21E-23 | 3.65E-21 | SYNRG  (synergin, gamma) | 0.69484 | 0.50084 |
|  | ENSECAG00000023280 | 12 | 21722550 | 21731721 | -11.6068 | 3.32E-18 | 2.27E-16 | FADS1  (fatty acid desaturase 1) | 0.6729 | 0.48977 |
| Muscle | ENSECAG00000017677 | 1 | 1.14E+08 | 1.14E+08 | 13.61197 | 1.19E-23 | 2.08E-21 | HERC2  (HECT and RLD domain containing E3 ubiquitin protein ligase 2) | 0.6413 | 0.47339 |
|  | ENSECAG00000011505 | 3 | 5744217 | 5890102 | 17.52678 | 1.84E-35 | 2.96E-32 | CHD9  (chromodomain helicase DNA binding protein 9) | 0.7025 | 0.50466 |
|  | ENSECAG00000006451 | 3 | 18334353 | 18335975 | 9.902519 | 5.43E-13 | 1.49E-11 | DDX28  (DEAD (Asp-Glu-Ala-Asp) box polypeptide 28) | 0.7841 | 0.54347 |
|  | ENSECAG00000016995 | 5 | 55853345 | 55897158 | 15.38217 | 1.21E-28 | 6.53E-26 | CAPZA1  (capping protein (actin filament) muscle Z-line, alpha 1) | 0.92616 | 0.60393 |
|  | ENSECAG00000003249 | 6 | 35501724 | 35501933 | 3.254749 | 7.49E-06 | 1.06E-04 | TSEN15  (TSEN15 tRNA splicing endonuclease subunit) | 0.72986 | 0.51802 |
|  | ENSECAG00000022647 | 9 | 6229159 | 6251198 | 12.97629 | 5.96E-22 | 7.12E-20 | CHMP4C  (charged multivesicular body protein 4C) | 1.29183 | 0.72523 |
|  | ENSECAG00000024499 | 10 | 58506453 | 58523225 | 1.941151 | 1.09E-03 | 8.74E-03 | FOXO3  (forkhead box O3) | 0.91477 | 0.59939 |
|  | ENSECAG00000014508 | 11 | 49675720 | 49688013 | 9.17594 | 6.05E-11 | 1.33E-09 | PLD2  (phospholipase D2) | 0.66424 | 0.48534 |
|  | ENSECAG00000007911 | 12 | 27072526 | 27083029 | 10.73278 | 5.26E-15 | 1.91E-13 | ANKRD13D  (ankyrin repeat domain 13 family, member D) | 0.66014 | 0.48322 |
|  | ENSECAG00000010641 | 13 | 41334685 | 41377271 | 9.883918 | 4.35E-13 | 1.23E-11 | UNKL  (unkempt family zinc finger-like) | 0.66723 | 0.48687 |
|  | ENSECAG00000025052 | 22 | 24529033 | 24564706 | 10.39155 | 4.33E-14 | 1.38E-12 | CBFA2T2  (core-binding factor, runt domain, alpha subunit 2; translocated to, 2) | 0.79744 | 0.54952 |
|  | ENSECAG00000012086 | 22 | 24576179 | 24592061 | 2.743281 | 7.61E-05 | 8.68E-04 | NECAB3  (N-terminal EF-hand calcium binding protein 3) | 0.72823 | 0.51724 |
|  | ENSECAG00000006302 | 24 | 42214122 | 42226036 | 2.730814 | 3.77E-05 | 4.67E-04 | SLC25A29  (solute carrier family, member 29) | 0.7676 | 0.53587 |
|  | ENSECAG00000018101 | 28 | 41466402 | 41532567 | 12.14179 | 3.38E-19 | 2.45E-17 | FBLN1  (fibulin 1) | 0.71941 | 0.51296 |

**Table S5.** Common genes between DEGs and selected genes associated with XP-EHH : XP-EHH cut-off value empirical p-value < 0.01 and XP-EHH value < -3.51551 significant SNPs in Thoroughbred were selected and > 1.73481 significant SNPs in Jeju domestic pony were selected

| **Sample Tissue** | **Ensembl ID** | **CHR** | **Start** | **End** | **DEG**  **logFC** | **DEG**  **P-value** | **DEG**  **FDR** | **Gene**  **symbol** | **# SNP** | **Mean of**  **XP-WHH** | **Mean of**  **XP-EHH P-value** |
| --- | --- | --- | --- | --- | --- | --- | --- | --- | --- | --- | --- |
| Blood | ENSECAG00000009700 | 1 | 49551464 | 49778390 | 12.20373 | 1.89E-19 | 1.46E-17 | ANK3  (ankyrin 3) | 387 | -3.86713 | 0.004582 |
|  | ENSECAG00000016283 | 1 | 92397477 | 92416004 | -14.5988 | 2.23E-26 | 5.06E-24 | ZNF592  (zinc finger protein 592) | 14 | -3.6937 | 0.006521 |
|  | ENSECAG00000000872 | 5 | 17363920 | 17397208 | -14.2221 | 1.38E-25 | 2.57E-23 | TOR1AIP1  (torsin A interacting protein 1) | 5 | -4.12532 | 0.00158 |
|  | ENSECAG00000002892 | 6 | 74016170 | 74027809 | 9.80616 | 1.34E-12 | 6.75E-11 | TIMELESS  (timeless circadian clock) | 8 | -4.44565 | 0.000802 |
|  | ENSECAG00000010127 | 7 | 4751960 | 4988487 | 12.37929 | 6.12E-20 | 4.99E-18 | INSR  (insulin receptor) | 5 | -3.80987 | 0.004652 |
|  | ENSECAG00000016841 | 8 | 178527 | 243772 | -13.9264 | 2.75E-24 | 3.77E-22 | MED15  (mediator complex subunit 15) | 39 | -3.6645 | 0.006726 |
|  | ENSECAG00000017599 | 10 | 7457036 | 7478256 | 16.37556 | 9.76E-32 | 6.44E-29 | ZNF567  (zinc finger protein 567) | 46 | -3.6854 | 0.006615 |
|  | ENSECAG00000015925 | 11 | 2814986 | 2828951 | -17.23 | 1.81E-34 | 2.94E-31 | EIF4A3  (eukaryotic translation initiation factor 4A3) | 1 | -3.52526 | 0.009726 |
|  | ENSECAG00000019424 | 15 | 30121373 | 30696569 | 13.13288 | 3.3E-22 | 3.3E-20 | EXOC6B  (exocyst complex component 6B) | 9 | -3.94019 | 0.003525 |
|  | ENSECAG00000000689 | 16 | 17404324 | 17450056 | -12.9826 | 8.86E-22 | 8.53E-20 | PPP4R2  (protein phosphatase 4, regulatory subunit 2) | 23 | -3.89151 | 0.003803 |
|  | ENSECAG00000024965 | 26 | 33662424 | 33750590 | -15.2732 | 8.89E-29 | 2.81E-26 | DYRK1A  (dual-specificity tyrosine-(Y)-phosphorylation regulated kinase 1A) | 7 | -3.63808 | 0.007152 |
| Muscle | ENSECAG00000012338 | 1 | 68213418 | 68243651 | -2.78093 | 9.74E-05 | 0.001084 | URB2  (URB2 ribosome biogenesis 2 homolog) | 46 | -3.70844 | 0.006394 |
|  | ENSECAG00000024743 | 1 | 1.2E+08 | 1.2E+08 | 2.216988 | 0.000535 | 0.004777 | NEO1  (neogenin 1) | 9 | -3.81447 | 0.00455 |
|  | ENSECAG00000014020 | 1 | 1.24E+08 | 1.24E+08 | 3.265104 | 3.08E-06 | 4.58E-05 | CORO2B  (coronin, actin binding protein, 2B) | 37 | -3.66469 | 0.006936 |
|  | ENSECAG00000019874 | 2 | 19784715 | 19831890 | 9.284013 | 3.77E-11 | 8.48E-10 | INPP5B  (inositol polyphosphate-5-phosphatase, 75kDa) | 3 | 1.760273 | 0.009301 |
|  | ENSECAG00000020498 | 2 | 46247154 | 46251383 | 3.038198 | 6.32E-06 | 9E-05 | C1orf174  (chromosome 1 open reading frame 174) | 10 | 1.825307 | 0.007927 |
|  | ENSECAG00000009256 | 2 | 69289564 | 69318502 | 2.829719 | 0.000587 | 0.005185 | CPE  (carboxypeptidase E) | 11 | -3.75014 | 0.00526 |
|  | ENSECAG00000000244 | 3 | 2577934 | 2838642 | 3.18674 | 3.53E-06 | 5.19E-05 | ZNF423  (zinc finger protein 423) | 3 | 1.746867 | 0.009664 |
|  | ENSECAG00000012351 | 3 | 23181694 | 23195306 | 2.432645 | 0.000194 | 0.001993 | FUK  (fucokinase) | 2 | -3.54777 | 0.009122 |
|  | ENSECAG00000026996 | 3 | 25675996 | 25681658 | 8.656818 | 1.14E-09 | 2.24E-08 | SYCE1L  (synaptonemal complex central element protein 1 like) | 43 | -3.71331 | 0.006133 |
|  | ENSECAG00000024389 | 3 | 80719113 | 80802247 | -2.49741 | 0.000297 | 0.002887 | TEC  (tec protein tyrosine kinase) | 11 | -3.66016 | 0.006871 |
|  | ENSECAG00000016126 | 3 | 89324004 | 89367130 | -2.84043 | 7.72E-05 | 0.000878 | RELL1  (RELT-like 1) | 22 | -4.35804 | 0.003112 |
|  | ENSECAG00000016405 | 4 | 1.02E+08 | 1.02E+08 | 14.80328 | 1.98E-27 | 8.79E-25 | ZNF775  (zinc finger protein 775) | 6 | -3.61461 | 0.007576 |
|  | ENSECAG00000017203 | 5 | 74951 | 78919 | -2.60874 | 0.000161 | 0.001709 | BTG2  (BTG family, member 2) | 3 | 1.796523 | 0.00838 |
|  | ENSECAG00000018261 | 5 | 33996082 | 34163712 | -3.01813 | 1.43E-05 | 0.000195 | RGS4  (regulator of G-protein signaling 4) | 3 | -3.68571 | 0.006294 |
|  | ENSECAG00000019626 | 5 | 51494441 | 51512333 | -2.16498 | 0.001265 | 0.009912 | FAM46C  (family with sequence similarity 46, member C) | 5 | -3.54222 | 0.009277 |
|  | ENSECAG00000017101 | 5 | 52109720 | 52148091 | 10.72769 | 4.91E-15 | 1.79E-13 | PTGFRN  (prostaglandin F2 receptor inhibitor) | 190 | -3.83333 | 0.004681 |
|  | ENSECAG00000019594 | 5 | 54125841 | 54157241 | -2.43055 | 0.000744 | 0.006297 | DENND2C  (DENN/MADD domain containing 2C) | 1 | -3.70886 | 0.005683 |
|  | ENSECAG00000010817 | 5 | 56038131 | 56074031 | -3.01296 | 2.79E-05 | 0.000356 | CTTNBP2NL  (CTTNBP2 N-terminal like) | 63 | -3.81149 | 0.005189 |
|  | ENSECAG00000014896 | 7 | 4329680 | 4385853 | -10.3104 | 9.78E-14 | 2.96E-12 | VAV1  (vav 1 guanine nucleotide exchange factor) | 30 | -3.81222 | 0.005233 |
|  | ENSECAG00000000616 | 7 | 38020741 | 38088004 | 10.37942 | 4.58E-14 | 1.46E-12 | BARX2  (BARX homeobox 2) | 21 | -3.69688 | 0.006732 |
|  | ENSECAG00000006886 | 8 | 34550671 | 34667158 | -2.28078 | 0.001102 | 0.008807 | RAB31  (RAB31, member RAS oncogene family) | 331 | -3.9238 | 0.004322 |
|  | ENSECAG00000022158 | 8 | 79696766 | 79722287 | -13.8808 | 5.79E-25 | 1.36E-22 | VPS4B  (vacuolar protein sorting 4 homolog B) | 3 | -3.85375 | 0.003645 |
|  | ENSECAG00000022647 | 9 | 6229159 | 6251198 | 12.97629 | 5.96E-22 | 7.12E-20 | CHMP4C  (charged multivesicular body protein 4C) | 1 | -3.56699 | 0.008631 |
|  | ENSECAG00000021363 | 9 | 62858588 | 62998122 | 4.499673 | 2.56E-09 | 4.89E-08 | DEPTOR  (DEP domain containing MTOR-interacting protein) | 12 | -3.81331 | 0.005082 |
|  | ENSECAG00000013971 | 10 | 56408907 | 56520676 | 10.3044 | 8.11E-14 | 2.47E-12 | ATG5  (autophagy related 5) | 110 | -3.82991 | 0.005157 |
|  | ENSECAG00000024499 | 10 | 58506453 | 58523225 | 1.941151 | 0.001093 | 0.008744 | FOXO3  (forkhead box O3) | 2 | -3.62995 | 0.007238 |
|  | ENSECAG00000019067 | 11 | 23985788 | 23999212 | 11.86848 | 1.25E-18 | 8.11E-17 | OSBPL7  (oxysterol binding protein-like 7) | 1 | -3.55282 | 0.00899 |
|  | ENSECAG00000015368 | 11 | 37580509 | 37583009 | 8.465334 | 1E-08 | 1.8E-07 | PEX12  (peroxisomal biogenesis factor 12) | 1 | -3.577 | 0.008385 |
|  | ENSECAG00000017425 | 11 | 51672958 | 51741179 | -8.73255 | 2.9E-09 | 5.47E-08 | PIK3R5  (phosphoinositide-3-kinase, regulatory subunit 5) | 26 | -3.75586 | 0.005367 |
|  | ENSECAG00000014725 | 14 | 40760991 | 40922273 | 12.63797 | 9E-21 | 8.86E-19 | PITX1  (paired-like homeodomain 1) | 2 | -3.57615 | 0.008405 |
|  | ENSECAG00000007518 | 14 | 47635117 | 47670086 | -2.68765 | 0.000318 | 0.003068 | MARCH3  (membrane-associated ring finger (C3HC4) 3, E3 ubiquitin protein ligase) | 124 | -3.71976 | 0.006431 |
|  | ENSECAG00000007942 | 14 | 47692226 | 47739561 | -2.28296 | 0.000966 | 0.007839 | LMNB1  (lamin B1) | 116 | -4.46256 | 0.000852 |
|  | ENSECAG00000011406 | 14 | 68853182 | 68942841 | 8.651716 | 5.05E-09 | 9.27E-08 | ST8SIA4  (ST8 alpha-N-acetyl-neuraminide alpha-2,8-sialyltransferase 4) | 65 | -3.80435 | 0.005286 |
|  | ENSECAG00000000903 | 14 | 84754693 | 84910772 | 2.862874 | 0.000133 | 0.001434 | RASGRF2  (Ras protein-specific guanine nucleotide-releasing factor 2) | 23 | -3.80045 | 0.005269 |
|  | ENSECAG00000020847 | 14 | 86477973 | 86642232 | 2.519297 | 0.000189 | 0.001948 | ARSB  (arylsulfatase B) | 30 | -3.72394 | 0.005929 |
|  | ENSECAG00000004289 | 15 | 10737295 | 10797292 | 9.128406 | 4.49E-11 | 9.99E-10 | INPP4A  (inositol polyphosphate-4-phosphatase, type I, 107kDa) | 7 | -3.64432 | 0.007787 |
|  | ENSECAG00000009610 | 15 | 85914791 | 85956240 | 12.27764 | 1.26E-19 | 1.03E-17 | RNF144A  (ring finger protein 144A) | 89 | -3.89837 | 0.004025 |
|  | ENSECAG00000000689 | 16 | 17404324 | 17450056 | 10.9353 | 8.94E-16 | 3.59E-14 | PPP4R2  (protein phosphatase 4, regulatory subunit 2) | 23 | -3.89151 | 0.003803 |
|  | ENSECAG00000016131 | 16 | 20621610 | 20787495 | -5.07897 | 1.36E-10 | 2.88E-09 | FRMD4B  (FERM domain containing 4B) | 3 | -3.53354 | 0.009504 |
|  | ENSECAG00000016372 | 17 | 4573937 | 4629149 | 10.45843 | 2.27E-14 | 7.56E-13 | SPATA13  (spermatogenesis associated 13) | 1 | -3.60592 | 0.007709 |
|  | ENSECAG00000015413 | 17 | 8971379 | 8991975 | -3.26977 | 5.65E-06 | 8.1E-05 | SLC7A1  (solute carrier family 7 member 1) | 5 | -3.61543 | 0.007614 |
|  | ENSECAG00000000879 | 17 | 21719935 | 21817311 | 14.50011 | 2.9E-26 | 1.01E-23 | CAB39L  (calcium binding protein 39-like) | 731 | -4.17556 | 0.002171 |
|  | ENSECAG00000002755 | 18 | 47662359 | 47663466 | 9.868204 | 8.61E-13 | 2.33E-11 | B3GALT1  (UDP-Gal:betaGlcNAc beta 1,3-galactosyltransferase, polypeptide 1) | 4 | -3.64494 | 0.007823 |
|  | ENSECAG00000020788 | 19 | 9611359 | 9672717 | 10.36718 | 3.75E-14 | 1.21E-12 | MECOM  (MDS1 and EVI1 complex locus) | 12 | -4.02371 | 0.003176 |
|  | ENSECAG00000001441 | 19 | 33563993 | 33586434 | 2.255299 | 0.000312 | 0.003015 | TNK2  (tyrosine kinase, non-receptor, 2) | 12 | 1.819854 | 0.007972 |
|  | ENSECAG00000024988 | 19 | 36754148 | 36795593 | -3.1029 | 1.98E-05 | 0.000262 | PARP14  (poly (ADP-ribose) polymerase family, member 14) | 188 | -4.43196 | 0.001377 |
|  | ENSECAG00000020093 | 21 | 31604725 | 31669820 | 3.353725 | 8.86E-05 | 0.000995 | NPR3  (natriuretic peptide receptor C/guanylate cyclase C) | 6 | -3.60641 | 0.008081 |
|  | ENSECAG00000023860 | 22 | 20308216 | 20347901 | -11.9472 | 2.17E-18 | 1.35E-16 | TGM3  (transglutaminase 3) | 8 | -3.84544 | 0.004061 |
|  | ENSECAG00000013881 | 22 | 29084472 | 29249586 | 2.514713 | 0.000179 | 0.001853 | DHX35  (DEAH (Asp-Glu-Ala-His) box polypeptide 35 | 81 | -3.87529 | 0.004104 |
|  | ENSECAG00000014676 | 22 | 36998574 | 37106417 | -10.6894 | 7.17E-15 | 2.57E-13 | PREX1  (phosphatidylinositol-3,4,5-trisphosphate-dependent Rac exchange factor 1) | 4 | 1.750548 | 0.009563 |
|  | ENSECAG00000008989 | 23 | 51439380 | 51584439 | -9.40571 | 4.76E-12 | 1.16E-10 | AUH  (AU RNA binding protein/enoyl-CoA hydratase) | 5 | -3.74096 | 0.005355 |
|  | ENSECAG00000003862 | 24 | 33675191 | 33720018 | 9.35181 | 3E-11 | 6.79E-10 | C14orf102  (UPF0614 protein C14orf102-like) | 3 | -3.66206 | 0.006643 |
|  | ENSECAG00000024297 | 25 | 18941561 | 19101286 | -2.7057 | 0.000203 | 0.002075 | COL27A1  (collagen, type XXVII, alpha 1) | 5 | -3.56944 | 0.008613 |
|  | ENSECAG00000012050 | 26 | 32991276 | 33237308 | 10.65909 | 5.26E-15 | 1.91E-13 | HLCS  (holocarboxylase synthetase) | 5 | -3.82705 | 0.004542 |
|  | ENSECAG00000015894 | 27 | 34212375 | 34265602 | -2.93484 | 2.76E-05 | 0.000353 | ANGPT2  (angiopoietin 2) | 97 | 2.22192 | 0.002753 |
|  | ENSECAG00000003554 | 29 | 29291814 | 29303208 | 14.40568 | 7.89E-26 | 2.42E-23 | AKR1E2  (aldo-keto reductase family 1, member E2) | 1 | 1.76432 | 0.009193 |
|  | ENSECAG00000021968 | X | 18103355 | 18106014 | -2.69849 | 7.55E-05 | 0.000861 | SAT1  (spermidine/spermine N1-acetyltransferase 1) | 13 | 1.790818 | 0.008567 |

**Table S6.** List of basic stats such as the number of transcripts, components, and contig N50 value in RNA-seq whole reads and unmapped reads by trinity *de novo* assembly**.**

| Sample ID | Reads | transcripts | components | Contig N50 |
| --- | --- | --- | --- | --- |
| BF1B | Whole | 215506 | 159877 | 1814 |
|  | Unmapped | 5597 | 4914 | 421 |
| BF1P | Whole | 200291 | 169168 | 1358 |
|  | Unmapped | 775 | 717 | 373 |
| BF2B | Whole | 219397 | 186218 | 1114 |
|  | Unmapped | 6197 | 5545 | 388 |
| BF2P | Whole | 201725 | 167243 | 1418 |
|  | Unmapped | 634 | 603 | 370 |
| BF3B | Whole | 194689 | 160340 | 1584 |
|  | Unmapped | 1070 | 999 | 351 |
| BF3P | Whole | 197659 | 165630 | 1389 |
|  | Unmapped | 1111 | 1034 | 354 |
| BS1B | Whole | 206071 | 173251 | 1294 |
|  | Unmapped | 5277 | 4624 | 431 |
| BS1P | Whole | 221676 | 184356 | 1375 |
|  | Unmapped | 6285 | 5478 | 454 |
| BS2B | Whole | 213508 | 174922 | 2051 |
|  | Unmapped | 3210 | 2881 | 411 |
| BS2P | Whole | 235880 | 193273 | 1106 |
|  | Unmapped | 697 | 663 | 352 |
| BS3B | Whole | 189580 | 155230 | 1673 |
|  | Unmapped | 623 | 577 | 374 |
| BS3P | Whole | 176267 | 146704 | 1430 |
|  | Unmapped | 5440 | 4772 | 458 |
| MF1B | Whole | 108250 | 95928 | 826 |
|  | Unmapped | 241 | 259 | 361 |
| MF1P | Whole | 96293 | 86030 | 833 |
|  | Unmapped | 246 | 237 | 342 |
| MF2B | Whole | 84468 | 75565 | 813 |
|  | Unmapped | 4478 | 4136 | 384 |
| MF2P | Whole | 86727 | 78188 | 725 |
|  | Unmapped | 432 | 414 | 376 |
| MF3B | Whole | 98665 | 88042 | 964 |
|  | Unmapped | 621 | 599 | 372 |
| MF3P | Whole | 74551 | 68106 | 674 |
|  | Unmapped | 2403 | 2268 | 354 |
| MS1B | Whole | 100646 | 89752 | 774 |
|  | Unmapped | 165 | 158 | 413 |
| MS1P | Whole | 93819 | 84093 | 858 |
|  | Unmapped | 164 | 160 | 369 |
| MS2B | Whole | 85543 | 76894 | 977 |
|  | Unmapped | 682 | 661 | 369 |
| MS2P | Whole | 92927 | 82949 | 992 |
|  | Unmapped | 710 | 688 | 362 |
| MS3B | Whole | 76011 | 68916 | 862 |
|  | Unmapped | 191 | 182 | 401 |
| MS3P | Whole | 81533 | 73773 | 841 |
|  | Unmapped | 131 | 125 | 480 |

**Table S7.** Number of annotated transcripts from RNA-seq unmapped reads by trinity *de novo* assembly. The number in the parentheses is the number of transcripts that were not included in the results of the reference-based analysis.

| Sample ID | BF1B | BF1P | BF2B | BF2P | BF3B | BF3P |
| --- | --- | --- | --- | --- | --- | --- |
| Annotated transcripts | 53(3) | 5(1) | 55(4) | 7(0) | 12(1) | 12(1) |
| Sample ID | **BS1B** | **BS1P** | **BS2B** | **BS2P** | **BS3B** | **BS3P** |
| Annotated transcripts | 49(3) | 83(5) | 44(3) | 14(0) | 10(1) | 118(5) |
| Sample ID | **MF1B** | **MF1P** | **MF2B** | **MF2P** | **MF3B** | **MF3P** |
| Annotated transcripts | 8(1) | 9(2) | 147(21) | 14(3) | 18(4) | 59(8) |
| Sample ID | **MS1B** | **MS1P** | **MS2B** | **MS2P** | **MS3B** | **MS3P** |
| Annotated transcripts | 6(2) | 6(1) | 25(6) | 28(15) | 14(4) | 6(1) |

**Table S8.** Basic information of 4 horses re-sequencing data

| Individual | HORSE1(F2) | HORSE2(F1) | HORSE3(F3) | HORSE4(S3) |
| --- | --- | --- | --- | --- |
| Gender | Male | Male | Male | Female |
| Sample | Blood | Blood | Blood | Blood |
| Location | BGI | BGI | BGI | BGI |
| Sequencing depth | 10X | 10X | 10X | 10X |
| Read Length | 90 | 90 | 90 | 90 |
| Encoding | illumina 1.5 | illumina 1.5 | illumina 1.5 | illumina 1.5 |
| No. Raw Read | 314,274,368 | 302,024,230 | 296,743,324 | 282,784,938 |
| No. Read | 285,815,336 | 273,659,950 | 270,715,242 | 256,521,664 |
| Trimming | 5bp | 5bp | 5bp | 5bp |
| No. mapped read | 261,682,398 | 248,053,944 | 247,267,550 | 235,131,946 |
| Mapping rate (%) | 91.56 | 90.64 | 91.34 | 91.66 |

**Table S9.** RT-PCR primer information such as the gene symbol, direction and sequence

| **Gene Symbol** | **Direction** | **Sequence** |
| --- | --- | --- |
| ***TIMELESS*** | Forward | TAG TGC CCT TGG GTA CTT GG |
|  | Reverse | TGC TGG ATA AGG ATG GGA AG |
| ***EIF4A3*** | Forward | GCT GAT TTG ATT TGC CTT |
|  | Reverse | GTT GTT GGG CAG GTC GTA GT |
| ***PIGW*** | Forward | GGG GCA GGA ATG TTC TAT CA |
|  | Reverse | AAA GTC CAC AGC CAA AAT GG |
| ***ANK3*** | Forward | TGG CAG AAC GAG ACA TCA AG |
|  | Reverse | ACA TGG CTT CCA TTT GCT TC |
| ***MSH3*** | Forward | AGC AGC AGA AAG ATG CCA TT |
|  | Reverse | GCC TTT AAC GCT GCT GTT TC |
| ***SYNRG*** | Forward | TGT CTC AAC TCG GAC AGC AC |
|  | Reverse | GAG TCA TCC AGG GTT CCT GA |
| **ASGR2** | Forward | ATC TGC GCA TCC TAG CTT GT |
|  | Reverse | ATA TGA AAG GGG CTC GTG TG |

***Supplementary Figures***

**
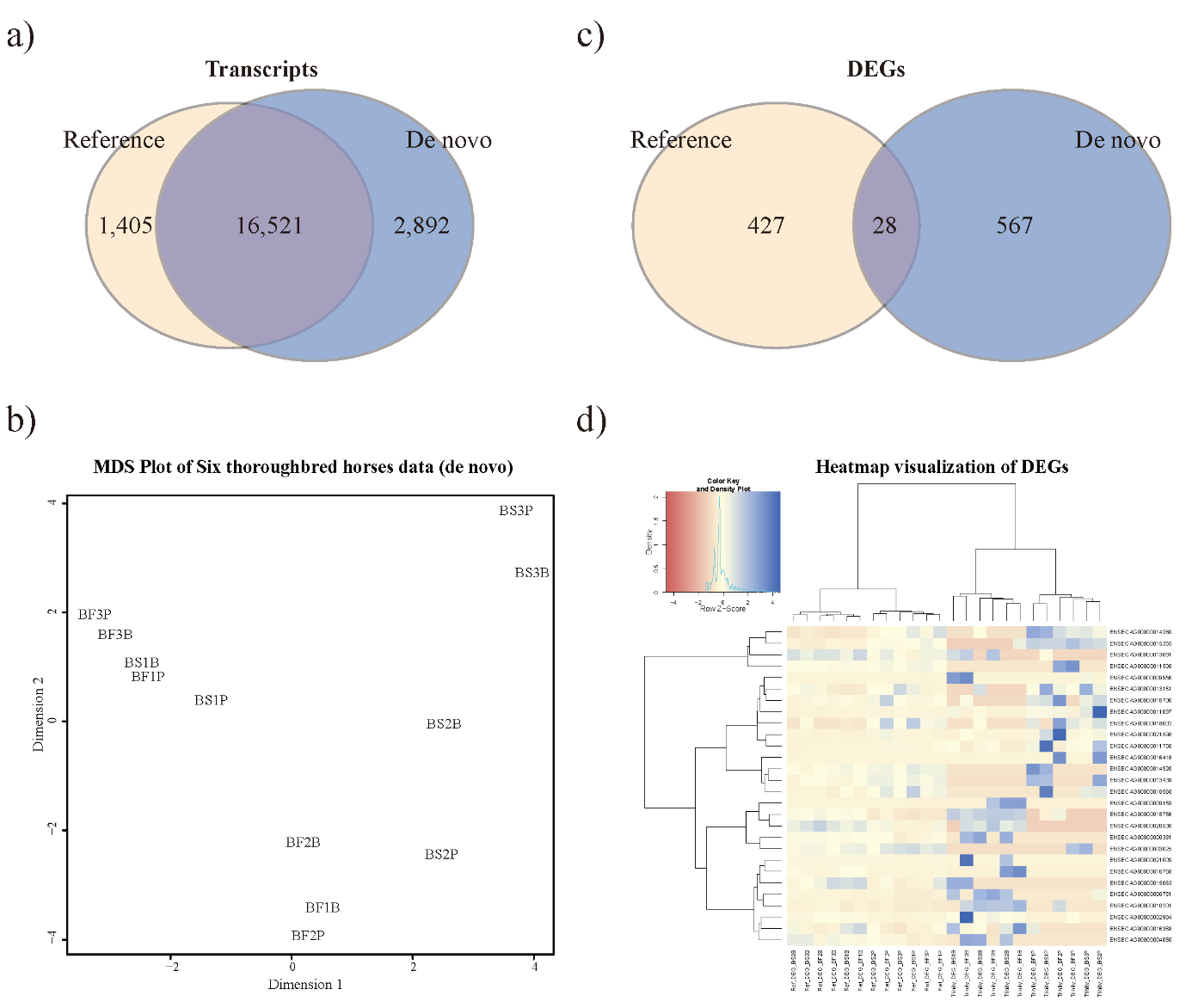
**

**Figure S1**. **Summary of comparative analysis between *de novo* assemble and reference genome assemble from blood in six Thoroughbred horses before and after exercise RNA-seq data (Total 12 samples).** a) The number of common transcripts of 12 samples between *de novo* assemble and reference genome assemble b) MDS plot of six Thoroughbred horses before and after exercise using *de novo* assemble. c) The number of DEGs between *de novo* assemble and reference genome assemble. d) Heat-map visualization of common DEGs between *de novo* assemble and reference genome assemble: rows represent DEGs from blood and columns represent assemble method from 6 horse samples (*First ‘B’ is for Blood and ‘M’ is for muscle. ‘F1’, ‘F2’, ‘F3’ and ‘S3’ are horse samples. Last ‘B’ is for ‘before exercise’ and ‘P’ is for ‘after exercise’)


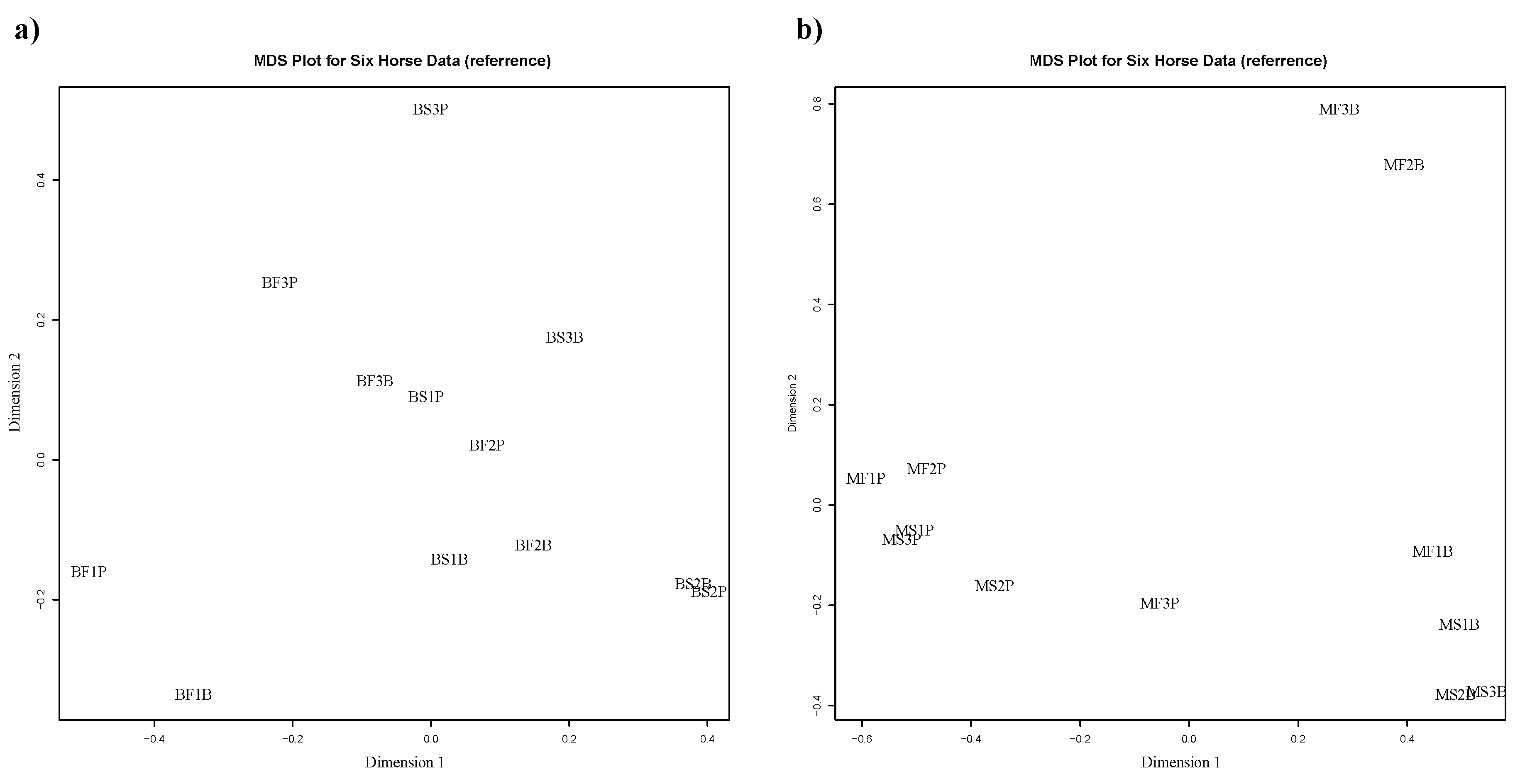


**Figure S2**. **MDS plot of six Thoroughbred horses before and after exercise using reference genome assemble in RNA-seq.** a) MDS plot of blood tissue in six Thoroughbred horse before and after exercise. b) MDS plot of skeletal muscle tissue in six Thoroughbred horse before and after exercise. (*First ‘B’ is for Blood and ‘M’ is for muscle. ‘F1’, ‘F2’, ‘F3’ and ‘S3’ are horse samples. Last ‘B’ is for ‘before exercise’ and ‘P’ is for ‘after exercise’)


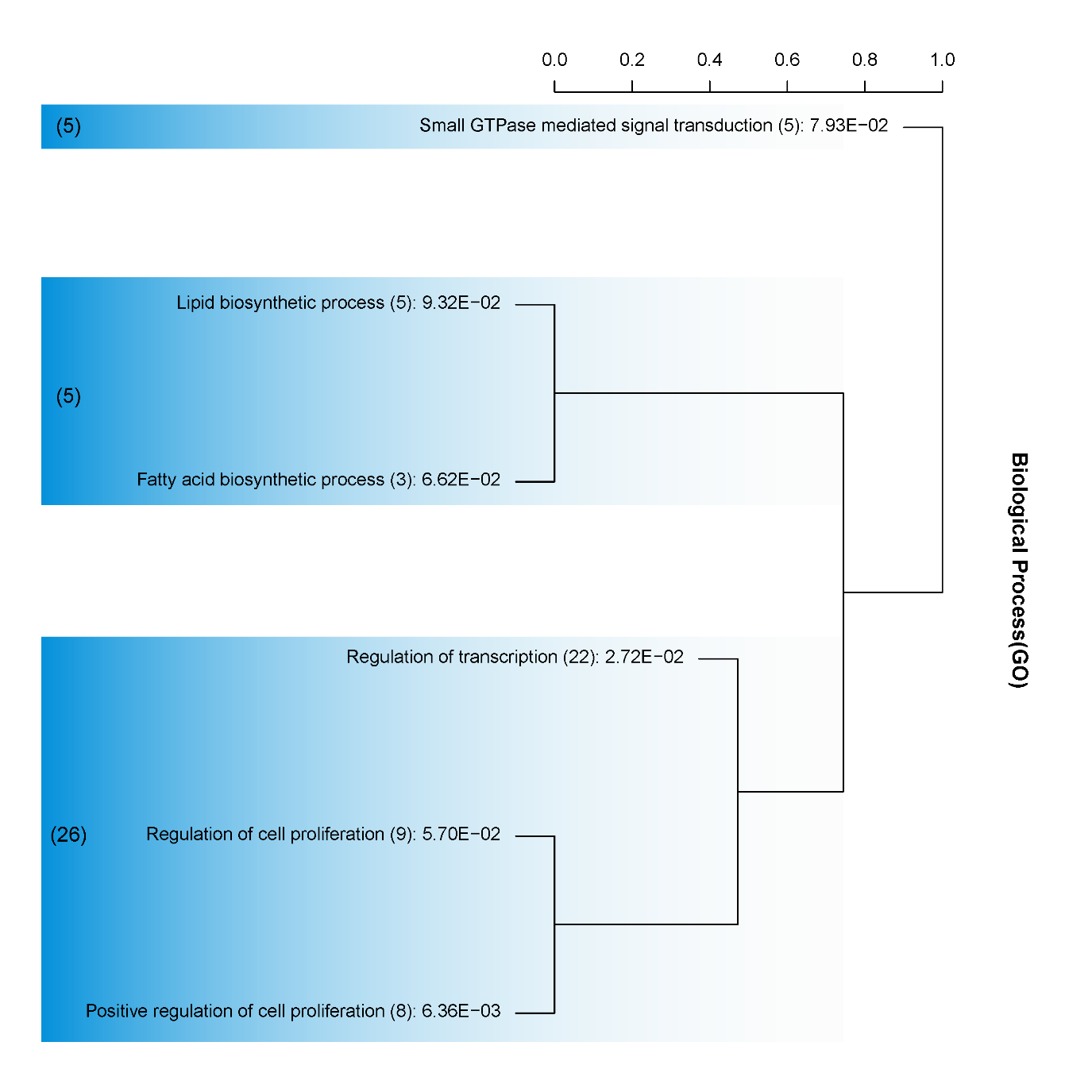


**Figure S3. Hierarchical clustering of biological process GO terms associated with up-regulated DEGs in blood.** The gene list of each GO term clustered using DAVID was compared to calculate the distance between the GO terms. For a distance value >0.5, GO terms were re-clustered, and GO term groups are shown as light-blue graduated blocks. The number of genes associated with the re-clustered GO term group is shown on the left side of the block.


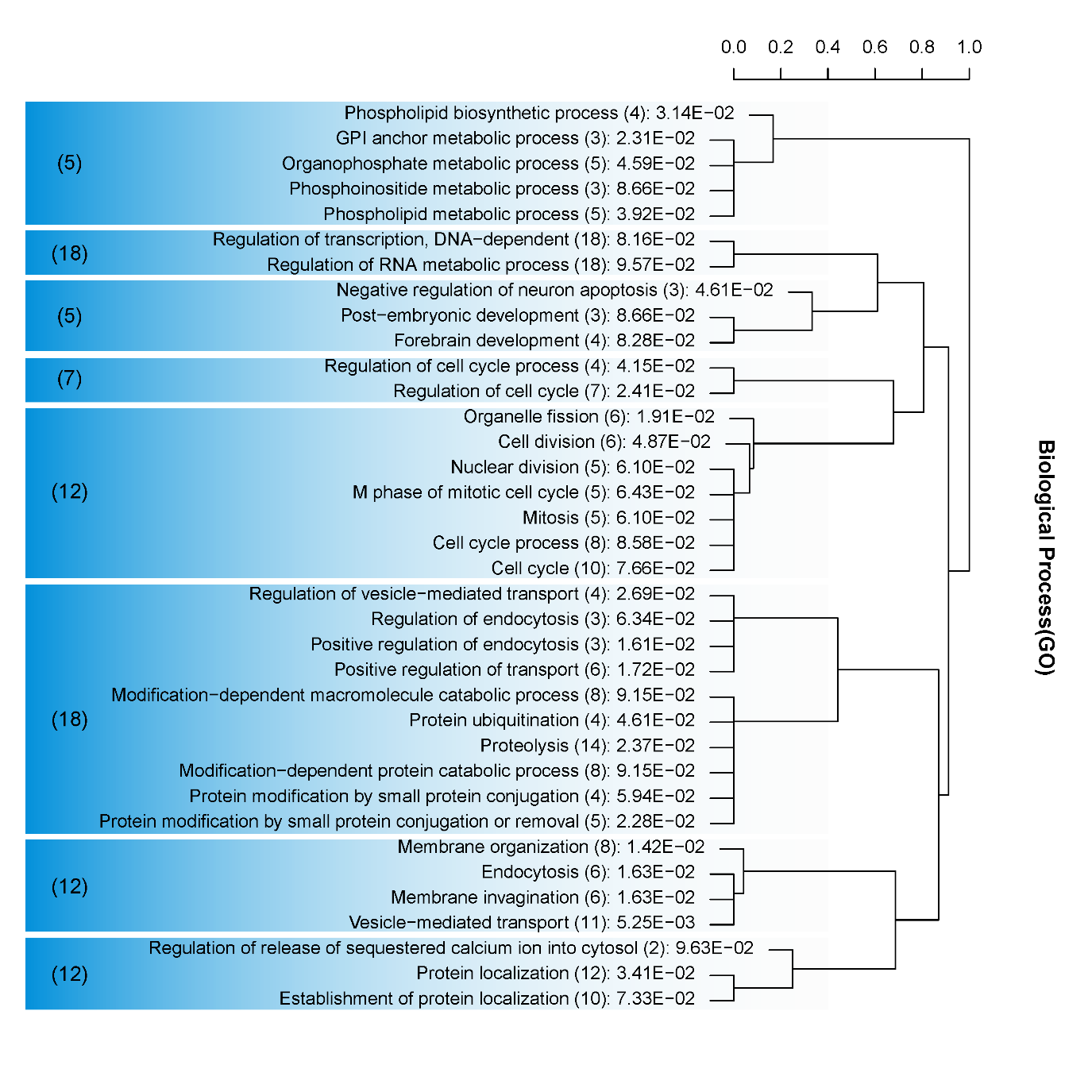
**Figure S4. Hierarchical clustering of biological process GO terms associated with down-regulated DEGs in blood.** The gene list of each GO term clustered using DAVID was compared to calculate the distance between the GO terms. For a distance value >0.5, GO terms were re-clustered, and GO term groups are shown as light-blue graduated blocks. The number of genes associated with the re-clustered GO term group is shown on the left side of the block.


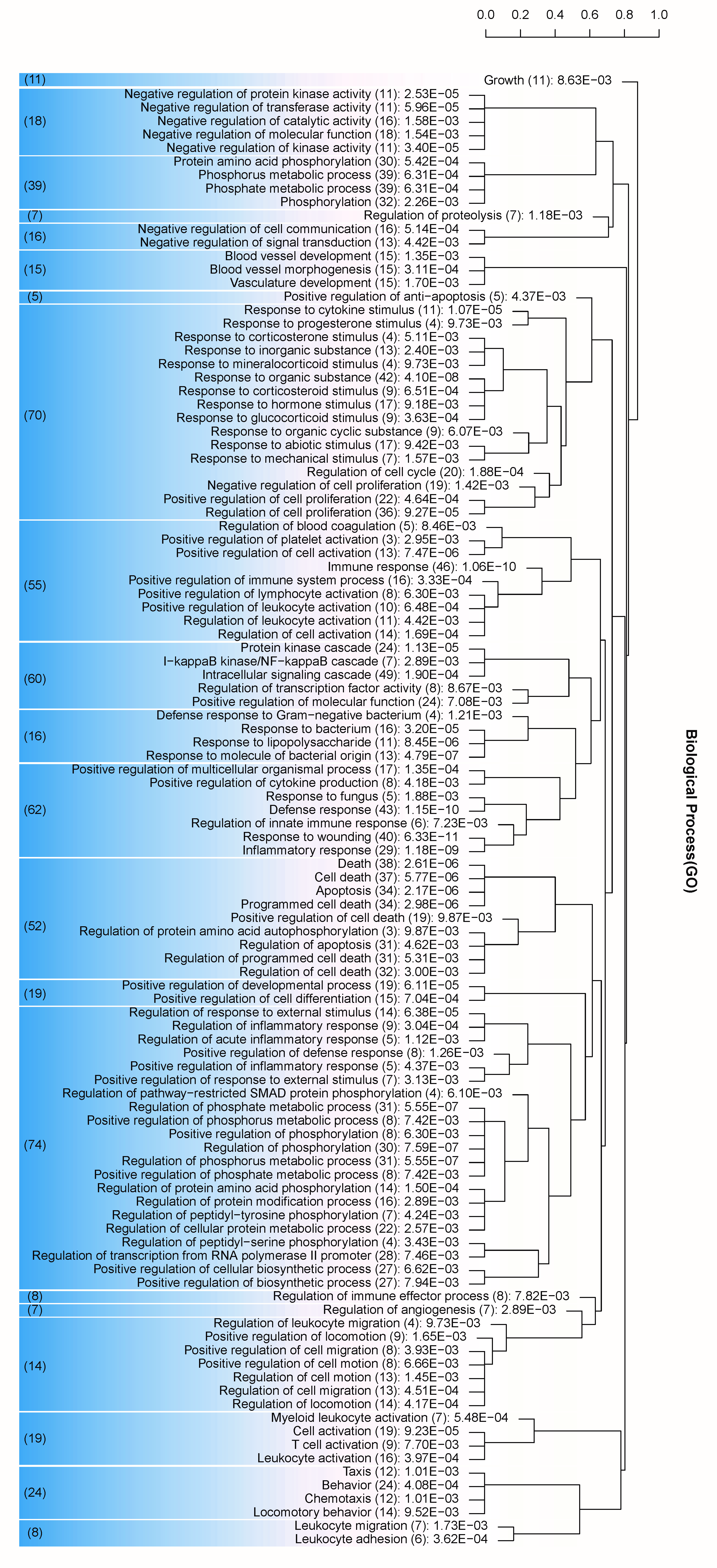


**Figure S5. Hierarchical clustering of biological process GO terms associated with up-regulated DEGs in muscle.** The gene list of each GO term clustered using DAVID was compared to calculate the distance between the GO terms. For a distance value >0.5, GO terms were re-clustered, and GO term groups are shown as light-blue graduated blocks. The number of genes associated with the re-clustered GO term group is shown on the left side of the block.


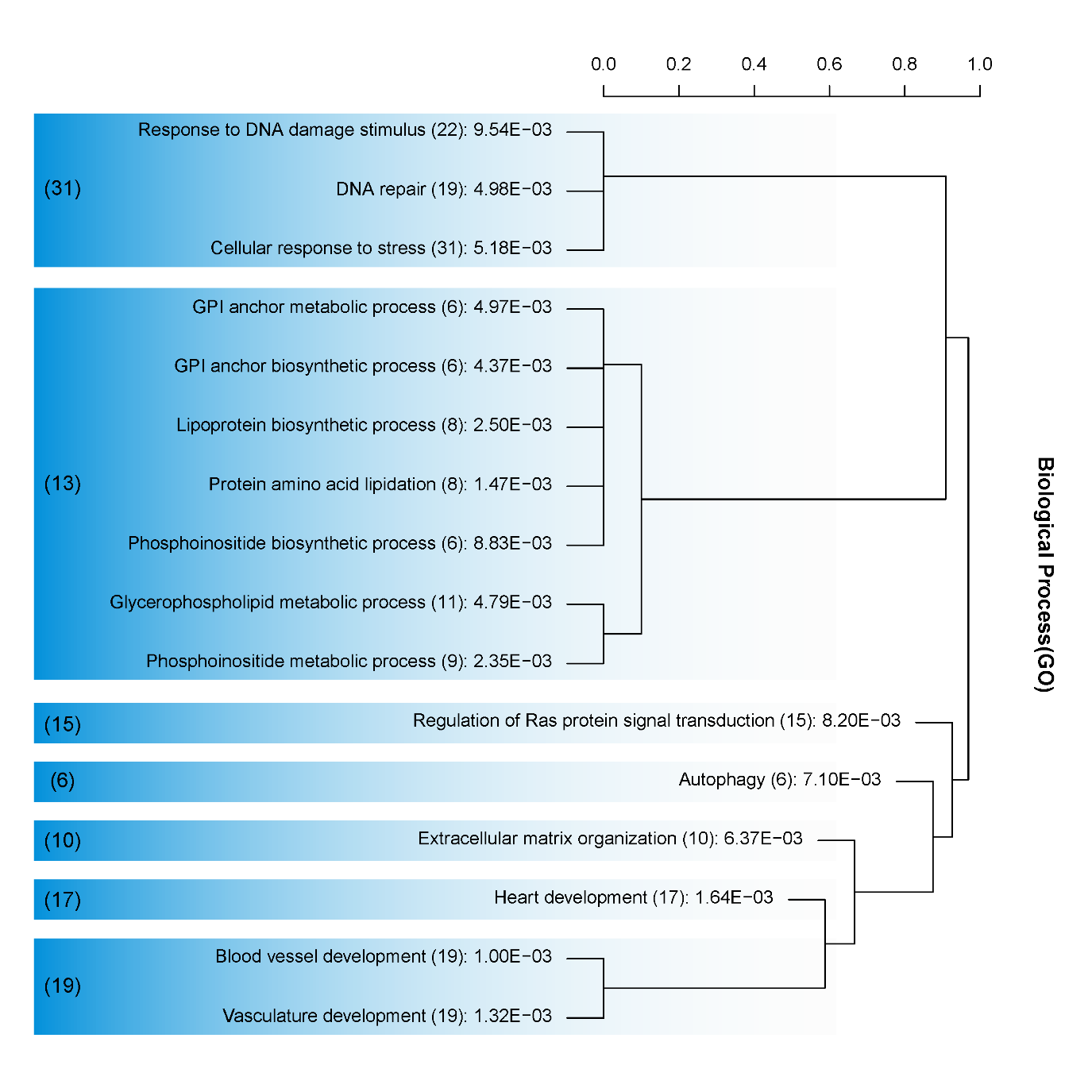
**Figure S6. Hierarchical clustering of biological process GO terms associated with down-regulated DEGs in muscle.** The gene list of each GO term clustered using DAVID was compared to calculate the distance between the GO terms. For a distance value >0.5, GO terms were re-clustered, and GO term groups are shown as light-blue graduated blocks. The number of genes associated with the re-clustered GO term group is shown on the left side of the block.


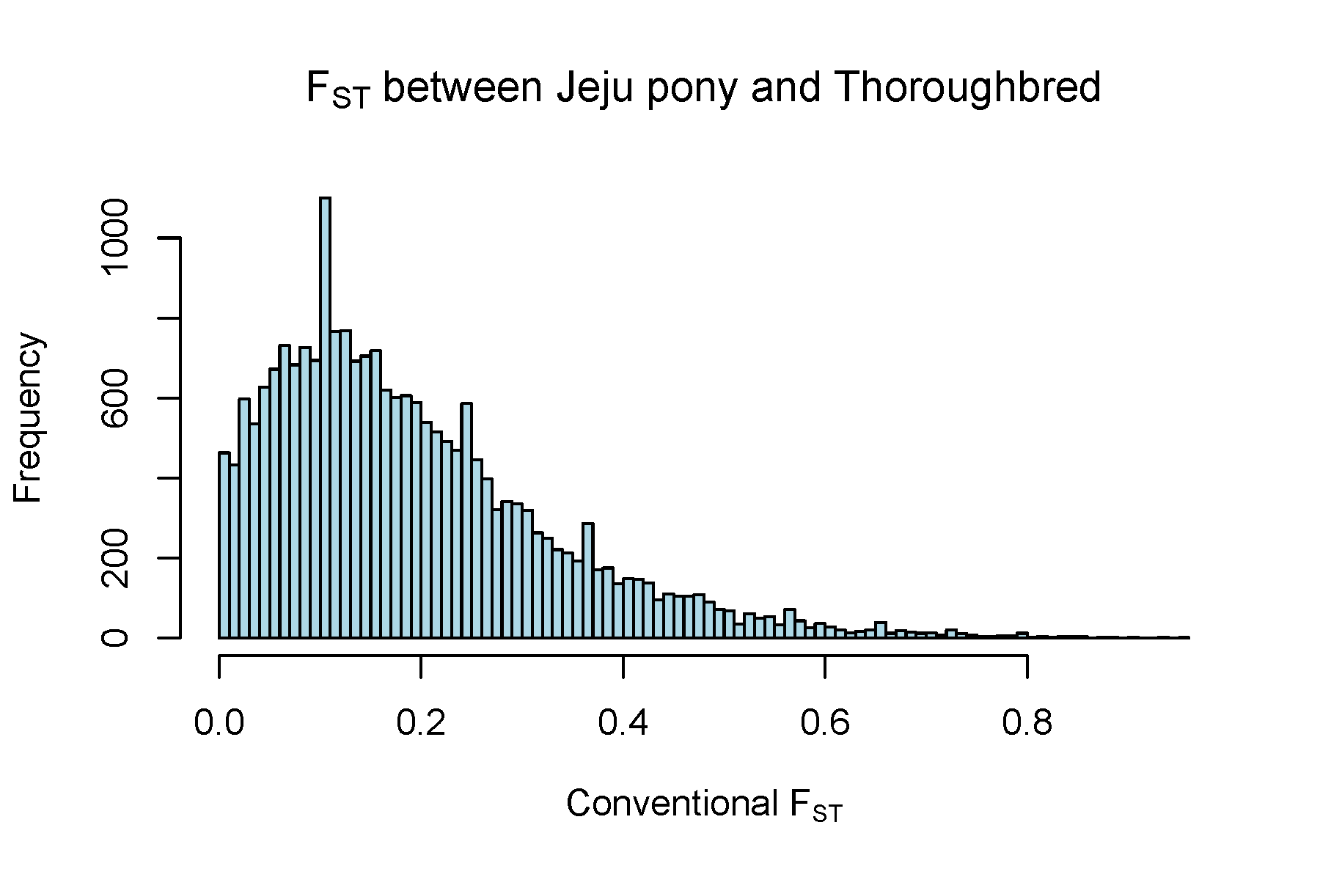


**Figure S7**. **Histogram of conventional F_ST_ frequency between Thoroughbred and jeju pony.** x-axis is conventional F_ST_ value, y-axis is gene frequency


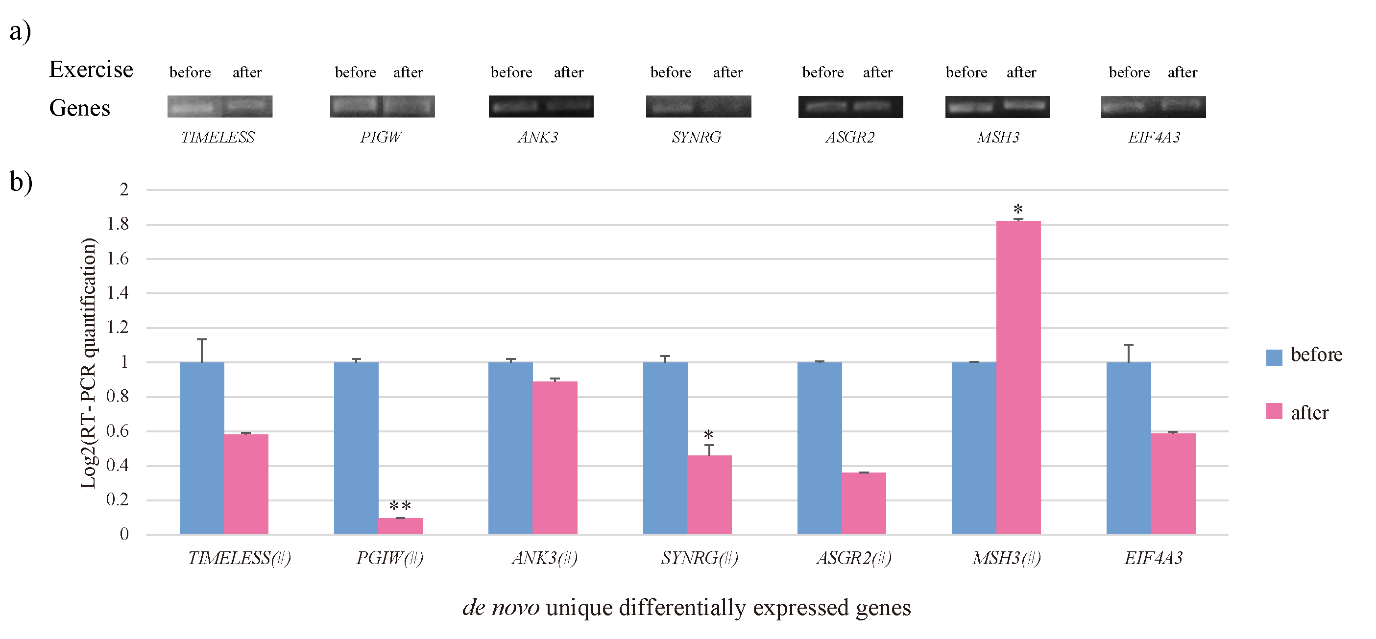


**Figure S8. qRT-PCR validation of *de novo* unique differentially expressed genes (DUDEGs) identified from the RNA-seq data set of Thoroughbred horses before and after exercise:** a) RT-PCR of six DUDEG in horses before exercise and after exercise. b) qRT-PCR results depicted as C_t_ value was calculated using 2^-ΔΔCt^ method. *: p-value < 0.05. **: p-value < 0.01. #: The expression patters of genes supported the result of our analysis
